# The Cytochalasins and Polyketides from a Mangrove Endophytic Fungus *Xylaria arbuscula* QYF

**DOI:** 10.3390/md22090407

**Published:** 2024-09-05

**Authors:** Qi Tan, Xinyu Ye, Siqi Fu, Yihao Yin, Yufeng Liu, Jianying Wu, Fei Cao, Bo Wang, Tingshun Zhu, Wencong Yang, Zhigang She

**Affiliations:** 1 School of Chemistry, Sun Yat-sen University, Guangzhou 510006, China; tanq27@mail2.sysu.edu.cn (Q.T.); yexy55@mail3.sysu.edu.cn (X.Y.); yinyh6@mail2.sysu.edu.cn (Y.Y.); liuyf76@mail2.sysu.edu.cn (Y.L.); wujy89@mail2.sysu.edu.cn (J.W.); ceswb@mail.sysu.edu.cn (B.W.); zhutshun@mail.sysu.edu.cn (T.Z.); 2 College of Pharmaceutical Sciences, Hebei University, Baoding 071002, China; 15703382700@163.com (S.F.); caofei542927001@163.com (F.C.); 3 School of Pharmaceutical Sciences, Sun Yat-sen University, Guangzhou 510006, China

**Keywords:** mangrove endophytic fungus, *Xylaria arbuscula*, cytochalasin, polyketide, antimicrobial activity, cytotoxic activity

## Abstract

Twelve compounds, including four undescribed cytochalasins, xylariachalasins A–D (**1**–**4**), four undescribed polyketides (**5**–**8**), and four known cytochalasins (**9**–**12**), were isolated from the mangrove endophytic fungus *Xylaria arbuscula* QYF. Their structures and absolute configurations were established by extensive spectroscopic analyses (1D and 2D NMR, HRESIMS), electronic circular dichroism (ECD) calculations, ^13^C NMR calculation and DP4+ analysis, single-crystal X-ray diffraction, and the modified Mosher ester method. Compounds **1** and **2** are rare cytochalasin hydroperoxides. In bioactivity assays, Compound **2** exhibited moderate antimicrobial activities against *Staphylococcus aureus* and *Candida albicans* with MIC values of 12.5 μM for both Compound **10** exhibited significant cytotoxic activity against MDA-MB-435 with an IC_50_ value of 3.61 ± 1.60 μM.

## 1. Introduction

The mangrove forest ecosystem, located in tropical and subtropical intertidal estuarine zones, is one of the most productive ecosystems at the junction of land and sea [1,2]. In order to adapt to this special marine environment, mangrove endophytic fungi have evolved unique biological metabolic pathways to produce abundant structurally novel and diverse bioactive secondary metabolites [3,4], which have attracted significant attention from organic chemists and pharmacologists [5]. The *Xylaria* genus, belonging to the family of Xylariaceae, is generally found among saprophytic and endophytic fungi [6]. Previous studies have proved that the genus *Xylaria* can produce various kinds of secondary metabolites including cytochalasins [7], polyketides [8], terpenes [9], alkaloids [10], etc. Moreover, most of them possess promising pharmacological activities associated with drug discovery, such as cytotoxic [11], antimalarial [12], antimicrobial [13], and *α*-glucosidase inhibitory activities [14].

**Figure 1:**
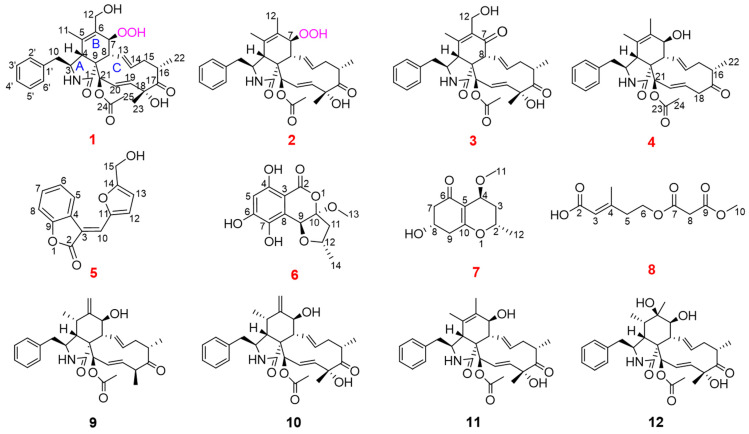
Structures of Compounds **1**–**12**.

During our ongoing exploration of new bioactive natural products from mangrove endophytic fungi [3,15,16,17,18,19], the chemical investigation of the fungus *Xylaria arbuscula* QYF collected from the mangrove plant *Kandelia candel*, led to the isolation of four undescribed cytochalasins, xylariachalasins A–D (**1**–**4**), four undescribed polyketides (**5**–**8**), and four known cytochalasins (**9**–**12**) (Figure 1). The known cytochalasins were identified as zygosporin E (**9**) [20], cytochalasin D (**10**) [21], cytochalasin C (**11**) [22], and cytochalasin O (**12**) [23]. Antimicrobial and cytotoxic activities of all isolated compounds were tested. Herein, the details of isolation, structure elucidation, and bioactivities of these compounds are reported.

## 2. Results and Discussion

### 2.1. Structure Identification

Xylariachalasin A (**1**) was gained as a white powder. Its molecular formula C_30_H_37_NO_8_ with thirteen degrees of unsaturation was deduced by the ion peak of HR-ESI-MS *m*/*z* [M + Na]^+^ 562.2435 (calcd. for 562.2411). The ^1^H and ^13^C NMR data (Table 1) as well as the HSQC spectrum of **1** showed one methyl doublet at *δ*_H_ 1.15 (d, *J* = 6.8 Hz, H_3_-22), three methyl singlets at *δ*_H_ 1.19 (s, H_3_-11), *δ*_H_ 1.50 (s, H_3_-23), and *δ*_H_ 2.33 (s, H_3_-25), one oxygenated methylene at *δ*_H_ 4.04 (d, *J* = 11.5 Hz, H-12*α*), and *δ*_H_ 4.22 (d, *J* = 11.5 Hz, H-12*β*), two pairs of *trans* carbon–carbon double bonds at *δ*_H_ 5.29 (dd, *J* = 15.4, 2.0 Hz, H-19), *δ*_H_ 5.96 (overlapped, H-20), and *δ*_H_ 5.34 (ddd, *J* = 15.4, 10.7, 5.0 Hz, H-14), *δ*_H_ 5.69 (dd, *J* = 15.5, 10.0 Hz, H-13), one ketone carbonyl (*δ*_C_ 211.6), one amide group (*δ*_C_ 176.7), one ester group (*δ*_C_ 172.1), and a mono substituted phenyl group (*δ*_C_ 128.0, 129.8, 130.7, and 138.9), which suggested it was a typical 10-phenyl cytochalasin.

**Figure 2:**
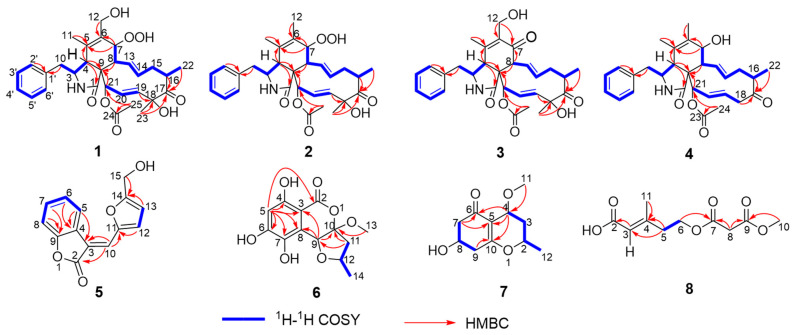
^1^H-^1^H COSY and HMBC correlation signals of Compounds **1**–**8**.

The ^1^H-^1^H COSY experiment of **1** displayed four proton spin–spin systems in the structure, namely H-2′/H-3′/H-4′/H-5′/H-6′, H-10/H-3/H-4, H-7/H-8/H-13/H-14/H-15/H-16/H_3_-22, and H-19/H-20/H-21 (Figure 2). The HMBC correlations from H-10 and H-2′ to C-1′ indicated that the phenyl group substitutes at C-10 (Figure 2). According to the HMBC correlations from H-4 to C-1, C-5, and C-9, H-7 to C-5 and C-6, H-8 to C-9, H_3_-11 to C-5, and H-12 to C-6, the fusing pattern of rings A and B can be determined. According to the HMBC correlations from H-16, H_3_-22, and H_3_-23 to C-17, H-19 and H_3_-23 to C-18, H_3_-25 to C-21 and C-24, and H-21 to C-9, ring C fused with ring B was elucidated. Therefore, the planar structure of Compound **1** was established. The rare hydroperoxyl at C-7 of Compound **1** was affirmed by the ^13^C NMR data of the oxygenated methine C-7 at *δ*_C_ 83.0 and the ion peak of HR-ESI-MS *m*/*z* [M + H − H_2_O]^+^ 522.2493 (calcd. for 522.2486) of **1** [24]. To the best of our knowledge, this is the second report of typical 10-phenyl cytochalasin possessing a hydroperoxyl group.

**Figure 3:**
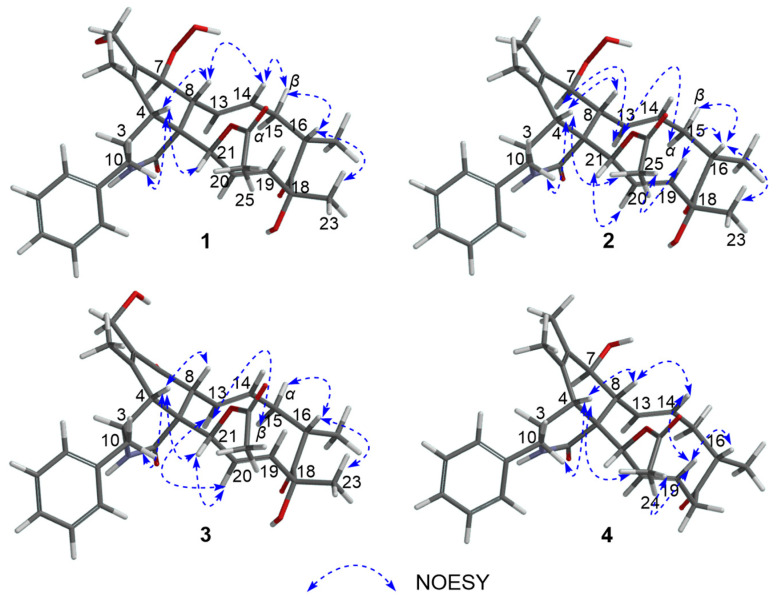
NOESY correlation signals of Compounds **1**–**4**.

**Figure 4:**
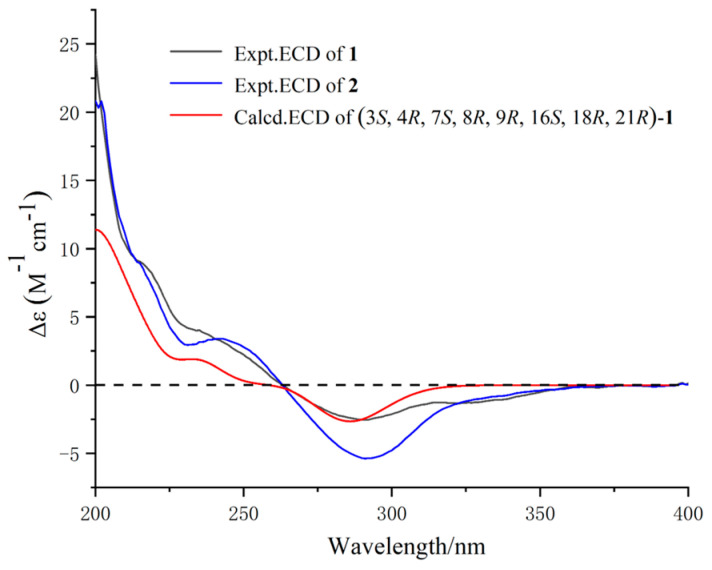
ECD spectra of Compounds **1** and **2**.

In the NOESY experiment, the NOE correlations (Figure 3) from H-4 to H-8, H-10, and H-21, from H-14 to H-8 and H-15*β*, and from H-16 to H-15*β* and H_3_-23, combined with the coupling constant of H-7/H-8 (^3^*J*_7,8_ = 10.0 Hz), H-14/H-15*α* (^3^*J*_14,15*α*_ = 10.7 Hz), and H-14/H-15*β* (^3^*J*_14,15*β*_ = 5.0 Hz) suggested the H*α*-3, H*β*-4, H*α*-7, H*β*-8, H*β*-16, H_3_*β*-23 orientations as well as the α-position for the 1-amide group. The ^13^C NMR calculation and DP4+ analysis were used to support the absolute configuration of C-21 of Compound **1** (21*R*-**1**, or 21*S*-**1**). The calculated result of 21*R*-**1** (*R^2^* = 0.9983) was a better match with the experimental data than that of 21*S*-**1** (*R^2^* = 0.9960) (Figure S78). Moreover, according to the DP4+ probability analysis, 21*R*-**1** was assigned with a 100.00% (Figure S79) probability. Combined with the comparison of the experimental ECD spectrum (Figure 4) of **1** with the calculated one, the absolute configuration of **1** was determined as 3*S,* 4*R*, 7S, 8*R*, 9*R*, 16*S*, 18*R*, and 21*R*.

**Figure 5:**
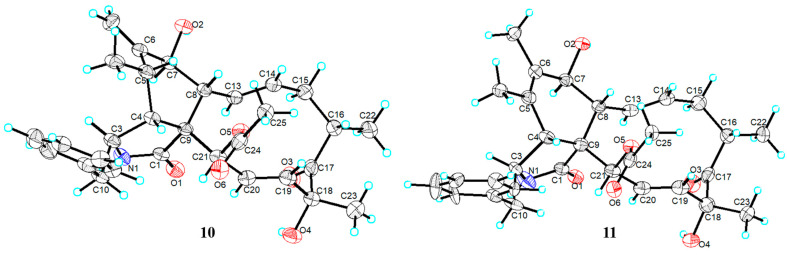
Single-crystal X-ray structures of Compounds **10** and **11**.

Fortunately, we got the qualified single-crystal of the known Compound **11** [Flack parameter of −0.1(2)] (Figure 5). The ^13^C NMR data in CD_3_OD of Compound **11** (Figure S80) was similar to that of Compound **1**. The main difference was that the methyl (*δ*_C_ 14.4) at C-6 and the hydroxyl at C-7 (*δ*_C_ 69.8) in Compound **11** was replaced by a hydroxymethyl (*δ*_C_ 58.7) and a hydroperoxyl (C-7, *δ*_C_ 83.0) in **1**, respectively**.** The single crystal of Compound **11** further proved the absolute configuration of Compound **1**.

From the biogenetic and structural point of view, the single-crystal of Compounds **10** and **11** (Figure 5) could be used as model compounds to support the assignment of the absolute configuration of new Compounds **1**–**4**.

Xylariachalasin B (**2**) was isolated as a white powder. Its molecular formula C_30_H_37_NO_7_ with thirteen degrees of unsaturation was deduced by the ion peak of HR-ESI-MS *m*/*z* [M + Na]^+^ 546.2456 (calcd. for 546.2462). The ^1^H and ^13^C NMR data (Table 1) displayed one methyl doublet [*δ*_H_ 1.21 (d, *J* = 6.8 Hz, H_3_-22)], four methyl singlets [*δ*_H_ 1.46 (s, H_3_-11), *δ*_H_ 1.52 (s, H_3_-23), *δ*_H_ 1.75 (s, H_3_-12), and *δ*_H_ 2.32 (s, H_3_-25)], two pairs of *trans* carbon-carbon double bonds [*δ*_H_ 5.16 (dd, *J* = 15.7, 2.4 Hz, H-19), *δ*_H_ 5.34 (m, H-20), and *δ*_H_ 5.95 (overlapped, H-14), *δ*_H_ 6.01 (dd, *J* = 15.7, 10.4 Hz, H-13)], one ketone carbonyl (*δ*_C_ 210.2), one amide group (*δ*_C_ 174.6), one ester group (*δ*_C_ 170.2), and a mono-substituted phenyl group (*δ*_C_ 127.2, 129.0, 129.2, and 137.6), which suggested that Compound **2** was also a typical 10-phenyl cytochalasin.

The ^1^H-^1^H COSY signals of H-2′/H-3′/H-4′/H-5′/H-6′ and the HMBC correlations from H-10 and H-2′ to C-1′ indicated that the phenyl group substitutes at C-10 (Figure 2). The ^1^H-^1^H COSY correlations of H-10/H-3/H-4, H-7/H-8/H-13/H-14/H-15/H-16/H_3_-22, and H-19/H-20/H-21, combined with the HMBC correlations from H-4 to C-1, C-5, and C-9, H-7 to C-5 and C-6, H-8 and H-21 to C-9, H_3_-11 to C-5, H_3_-12 to C-6, H-16, H_3_-22 and H_3_-23 to C-17, H-19 and H_3_-23 to C-18, H_3_-25 to C-21 and C-24 manifested the three rings A/B/C fused system. According to the ^13^C NMR data of C-7 (*δ*_C_ 83.2) and the ion peak of HR-ESI-MS *m*/*z* [M + H − H_2_O]^+^ 506.2536 (calcd. for 506.2537) of **2**, a rare hydroperoxyl at C-7 of Compound **2** was also confirmed. Thus, Compounds **2** and **1** were structurally similar; they are both rare cytochalasin hydroperoxides, with the main difference between them being that the hydroxymethyl at C-6 in **1** was replaced by methyl in **2**.

Though the overlapped chemical shifts [such as H-4 (*δ*_H_ 2.50) and H-15*β* (*δ*_H_ 2.52), H-8 (*δ*_H_ 3.00) and H-10 (*δ*_H_ 2.98)] influenced the determination of the relative configuration through NOE correlation, we could rule out the impossible case by the spatial structure of the Compound **2**. For example, the NOESY spectrum showed correlations of *δ*_H_ 2.50 (H-4, H-15*β*) and *δ*_H_ 3.00 (H-8, H-10). According to the space structure of Compound **2**, the NOE correlation was between H-4 and H-8 (H-10), not between H-15*β* and H-8 (H-10). Combined with the biosynthesis of cytochalasins [25], the relative configuration of H-4, H-8, and H-10 can be confirmed as H*α*-3, H*β*-4, and H*β*-8 orientations. Excluding the impact of overlapped chemical shifts through the spatial structure of Compound **2**, the NOE correlations from H-4 to H_3_-25, from H-13 to H-7, and H-15*α*, from H-16 to H-15*β,* H-19, and H_3_-23, from H-20 to H-21, and from H-19 to H_3_-25, combined with the large coupling constant of H-7/H-8 (^3^*J*_7,8_ = 10.2 Hz), suggested the H*α*-7, H*β*-16, H_3_*β*-23, H*α*-21 orientations and *α*-position for 1-amide group. Hence, Compounds **1** and **2** had the same relative configuration. The absolute configuration of **2** was elucidated by comparing the experimental ECD spectrum with **1** (Figure 4). So, the absolute configuration of **2** was defined as 3*S,* 4*R*, 7*S*, 8*R*, 9*R*, 16*S*, 18*R*, and 21*R*. 

Xylariachalasin C (**3**) was obtained as a white powder. Its molecular formula C_30_H_35_NO_7_ with fourteen degrees of unsaturation was deduced by the ion peak of HR-ESI-MS *m*/*z* [M + Na]^+^ 544.2309 (calcd. for 544.2306). The ^1^H and ^13^C NMR data of compound **3** (Table 2) were similar to that of **1**, which indicated that **3** was also a typical 10-phenyl cytochalasin. Further analysis of the ^1^H-^1^H COSY and the HMBC correlations (Figure 2) of **3** showed that Compound **3** had a ketone carbonyl (*δ*_C_ 199.7) in Position 7 instead of an oxygenated methine compared to Compound **1**, which was verified by the HMBC correlations from H-12 to C-6, and from H-8 and H-12 to C-7. Therefore, the planar structure of **3** was established, as shown in Figure 2.

**Figure 6:**
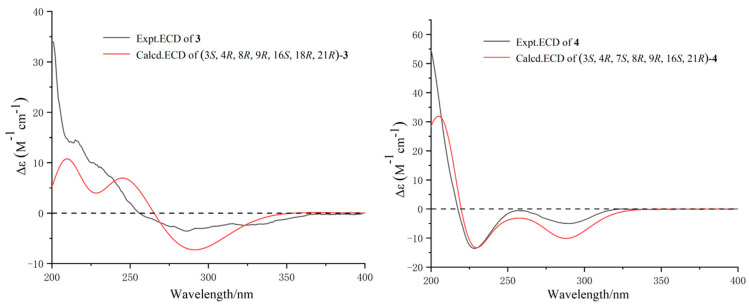
Experimental and calculated ECD spectra of **3** and **4**.

The relative configuration of **3** was established by the coupling constant of H-8/H-13 (^3^*J*_8,13_ = 9.7 Hz) and H-20/H-21 (^3^*J*_20,21_ = 2.4 Hz) as well as the key NOESY correlations (Figure 3) between H-4/H-8, H-10, H-21, H-13/H-15*β*, H-20, H-16/H-15*α*, H_3_-23, H-20/H-21. Hence, Compounds **3** and **1** had similar relative configurations. The absolute configuration of **3** was determined as 3*S,* 4*R*, 8*R*, 9*R*, 16*S*, 18*R*, and 21*R* by comparing the experimental ECD spectrum (Figure 6) with the calculated one and the ECD spectra of 7-oxo-cytochalasin C [26].

Xylariachalasin D (**4**) was isolated as a white powder. Its molecular formula C_29_H_35_NO_5_ with thirteen degrees of unsaturation was deduced by the ion peak of HR-ESI-MS *m*/*z* [M + Na]^+^ 500.2400 (calcd. for 500.2407). Comparing the 1D and 2D NMR data (Table 2, Figure 2) of **4** with **1** indicated the absence of a methyl and a hydroxyl at C-18 in **4**. The hydroperoxyl [*δ*_H_ 4.07 (d, *J* = 10.0 Hz), *δ*_C_ 83.0] at C-7 in **1** was replaced by a hydroxyl [*δ*_H_ 3.72 (d, *J* = 10.4 Hz), *δ*_C_ 70.1] in **4**, and the hydroxymethyl [*δ*_H_ 4.04 (d, *J* = 11.5 Hz), *δ*_H_ 4.22 (d, *J* = 11.5 Hz), *δ*_C_ 58.7] at C-6 in **1** was replaced by a methyl [*δ*_H_ 1.61, *δ*_C_ 14.4] in **4**. Therefore, the planar structure of **4** was established. Excluding the impact of overlapped chemical shifts through the spatial structure of Compound **4,** combined with the biosynthesis of cytochalasins [25], the relative configuration of **4** was established by the large coupling constant of H-7/H-8 (^3^*J*_7,8_ = 10.4 Hz) and the NOE correlations (Figure 3) of H-4/H-8, H-10, H_3_-24, H-14/H-8, H-19, and H-19/H-16, H_3_-24. The calculated ECD curve of 3*S,* 4*R*, 7*S*, 8*R*, 9*R*, 16*S*, and 21*R* was well matched with the experimental data of **4** (Figure 6), combined with the common biosynthetic pathway of cytochalasins in *Xylaria arbuscula* QYF, the absolute configuration of **4** was defined.

Compound **5** was obtained as a yellow oil. Its molecular formula C_14_H_10_O_4_ with ten degrees of unsaturation was deduced by the ion peak of HR-ESI-MS *m*/*z* [M + Na]^+^ 265.0467 (calcd. for 265.0471). The ^1^H NMR spectrum (Table 3) showed one oxygenated methylene [*δ*_H_ 4.74 (s, H-15)], and seven unsaturated protons [*δ*_H_ 6.65 (d, *J* = 3.4 Hz, H-13), 7.17 (overlapped, H-8 and H-12), 7.26 (t, *J* = 8.0 Hz, H-6), 7.40 (t, *J* = 8.0 Hz, H-7), 7.47 (s, H-10), and 8.58 (d, *J* = 8.0 Hz, H-5)]. The ^13^C NMR and HSQC spectrum displayed a total of 14 carbon signals, including one ester carbonyl (*δ*_C_ 171.4), five non-protonated sp^2^ carbons [*δ*_C_ 117.3, 123.6, and three oxygenated (*δ*_C_ 152.0, 155.4, and 162.0)], seven sp^2^ methine carbons (*δ*_C_ 111.3, 112.2, 124.1, 124.4, 125.3, 126.4, and 131.4), and one oxygenated methylene (*δ*_C_ 57.8).

**Figure 7:**
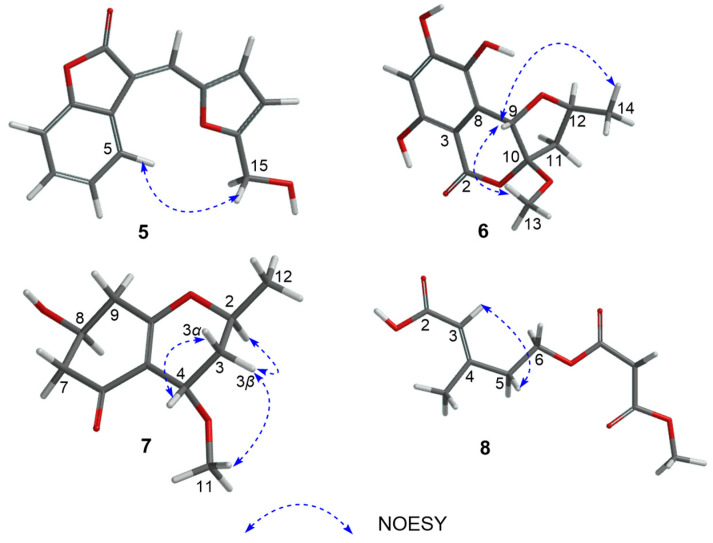
NOESY correlation signals of Compounds **5**–**8**.

The ^1^H-^1^H COSY correlations (Figure 2) of H-5/H-6/H-7/H-8, together with the HMBC from H-5 and H-6 to C-4, from H-7 and H-8 to C-9, from H-5 and H-10 to C-3, and from H-10 to C-2 indicated the existence of a 3-methylenebenzofuran-2(3*H*)-one moiety. Under the assistance of degrees of unsaturation, the ^1^H-^1^H COSY signals of H-12/H-13 together with HMBC correlations from H-12 to C-11, and from H-13 and H-15 to C-14 led to the identification of a 2-hydroxymethylfuran moiety. And the above two moieties were connected through C-10, according to HMBC correlations from H-10 to C-11. Thus, the planar structure of **5** was established. The stereochemistry of **5** was determined by NOESY correlations (Figure 7) of H-5/H-15, and the double bond at 3(10) was *E* isomer [27].

Compound **6** was isolated as a light-yellow oil. Its molecular formula C_13_H_14_O_7_ with seven degrees of unsaturation was deduced by the ion peak of HR-ESI-MS *m*/*z* [M − H]^−^ 281.0665 (calcd. for 281.0667). The ^1^H NMR spectrum (Table 3) revealed one methyl [*δ*_H_ 1.38 (d, *J* = 6.2 Hz, H_3_-14)], one methylene [*δ*_H_ 2.08 (dd, *J* = 12.8, 9.5 Hz, H-11*α*), and 2.73 (dd, *J* = 12.8, 6.2 Hz, H-11*β*)], one singlet methoxyl [*δ*_H_ 3.50 (s, H_3_-13)], two oxygenated methines [*δ*_H_ 4.39 (m, H-12), and 5.12 (s, H-9)], and one aromatic proton [*δ*_H_ 6.41 (s, H-5)]. The ^13^C NMR and HSQC spectrum indicated an ester carbonyl at *δ*_C_ 169.7, three oxygenated olefinic carbons at *δ*_C_ 139.4, 156.1, and 159.1, two non-protonated sp^2^ carbons at *δ*_C_ 98.0 and 121.4, a protonated olefinic carbon at *δ*_C_ 104.0, a ketal carbon at *δ*_C_ 113.8, two oxymethines at *δ*_C_ 70.8 and 76.3, a methoxyl at *δ*_C_ 53.5, a methylene at *δ*_C_ 43.5, and a methyl at *δ*_C_ 22.1. 

The planar structure of **6** was determined by comprehensive analysis of its 2D NMR data as follows (Figure 2). The ^1^H-^1^H COSY correlations of H-11/H-12/H_3_-14, combined with the HMBC correlations from H-9, H-11, and H_3_-13 to C-10, and from H-11 to C-9, established a 4-methoxy-2-methyltetrahydrofuran fragment. The HMBC correlations from H-5 to C-2, C-3, C-4, C-6, C-7, and C-8, and from H-9 to C-3 and C-8 revealed the presence of a 5,6,8-trihydroxyisochroman-1-one moiety, sharing C-9 and C-10 with the tetrahydrofuran fragment. Therefore, the planar structure of **6** was confirmed.

**Figure 8:**
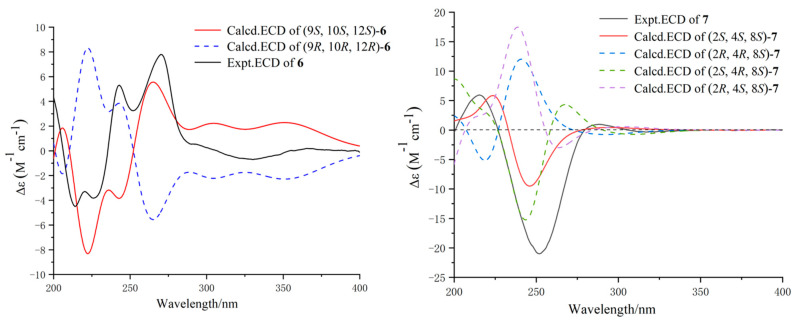
Experimental and calculated ECD spectra of **6** and **7**.

The relative configuration of **6** was identified by key NOESY correlations (Figure 7) of H-9/H_3_-13 and H_3_-14, indicating these protons were co-facial. Therefore, the relative configuration of **6** was assigned to be (9S*,10S*,12S*). Further, ECD calculations were used to clarify the absolute configuration of **6**. As shown in Figure 8, the calculated ECD curve of (9*S*, 10*S*, 12*S*)-**6** was similar to that of the experimental one, indicating that the absolute configuration of **6** was 9*S*, 10*S*, and 12*S*. 

Compound **7** was acquired as a brown oil. Its molecular formula C_11_H_16_O_4_ with four degrees of unsaturation was deduced by the ion peak of HR-ESI-MS *m*/*z* [M + Na]^+^ 235.0936 (calcd. for 235.0941). The ^1^H NMR spectrum (Table 4) showed one doublet methyl [*δ*_H_ 1.40 (d, *J* = 6.4 Hz, H_3_-12)], three methylenes [*δ*_H_ 1.38 (m, H-3*α*), *δ*_H_ 2.11 (m, H-3*β*), *δ*_H_ 2.45 (dd, *J* = 15.8, 9.0 Hz, H-7*α*), *δ*_H_ 2.60 (dd, *J* = 15.8, 4.4 Hz, H-7*β*), *δ*_H_ 2.48 (dd, *J* = 17.4, 8.4 Hz, H-9*α*), and 2.72 (dd, *J* = 17.4, 5.0 Hz, H-9*β*)], one singlet methoxyl [*δ*_H_ 3.35 (s, H_3_-11)], and three oxygenated methines [*δ*_H_ 4.15 (m, H-8), *δ*_H_ 4.19 (br.s, H-4), and 4.31 (m, H-2)]. The ^13^C NMR and HSQC spectrum revealed a ketone carbonyl (*δ*_C_ 197.9), two non-protonated sp^2^ carbons [*δ*_C_ 113.3, and one oxygenated (*δ*_C_ 174.0)], three oxygenated methines (*δ*_C_ 65.5, 67.1, and 72.0), a methoxyl (*δ*_C_ 56.7), three methylenes (*δ*_C_ 34.1, 38.7, and 46.7), and a methyl (*δ*_C_ 20.8). 

The ^1^H-^1^H COSY correlations of H-7/H-8/H-9 combined with the HMBC correlations from H-7 to C-5, C-6, from H-9 to C-10 established a cyclohexenone fragment. The ^1^H-^1^H COSY correlations of H_3_-12/H-2/H-3/H-4 combined with the HMBC correlations from H_3_-11 to C-4, from H-4 to C-5, C-6, C-10, indicated a 4-methoxy-2-methyltetrahydro-2*H*-pyran moiety was connected with the cyclohexenone fragment. Thus, the planar structure of **7** was confirmed, as shown in Figure 2. 

**Figure 9:**
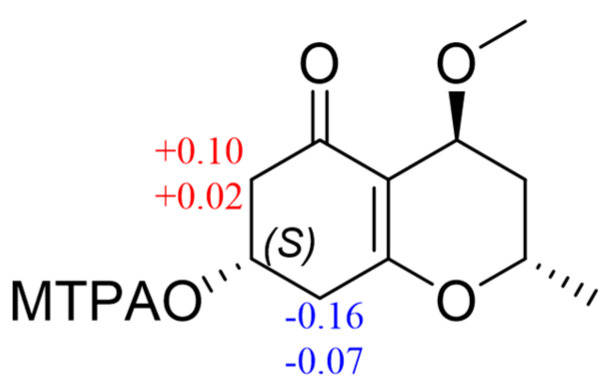
Δ*δ* (= *δS* − *δR*) values for (*S*)- and (*R*)-MTPA esters of **7**.

The absolute stereochemistry on C-8 of **7** was determined by the modified Mosher’s method [28]. The differences in ^1^H NMR chemical shifts between (*S*)- and (*R*)-MTPA esters (Δ*δ* = *δS* − *δR*) (Figure 9) were calculated, which proved that the configuration of C-8 was *S*. The NOE correlations of H-3*α*/H-4, and H-3*β*/H-2, H_3_-11 revealed that H-4 and H_3_-12 were co-facial (Figure 7). We calculated all the possible absolute configurations of **7** (Figure 8). The computed ECD curve of (2*S*, 4*S*, 8*S*)-**7** was well matched with the experimental one, so the absolute configuration of **7** was defined as 2*S*, 4*S*, and 8*S*.

Compound **8** was isolated as a brown oil. Its molecular formula C_10_H_14_O_6_ with four degrees of unsaturation was deduced by the ion peak of HR-ESI-MS *m*/*z* [M + Na]^+^ 253.0682 (calcd. for 253.0683). The ^1^H NMR spectrum (Table 4) showed one singlet methyl [*δ*_H_ 2.20 (s, H_3_-11)], three methylenes [*δ*_H_ 2.51 (t, *J* = 6.5 Hz, H-5), *δ*_H_ 3.38 (s, H-8), *δ*_H_ 4.31 (t, *J* = 6.5 Hz, H-6)], one singlet methoxyl [*δ*_H_ 3.74 (s, H_3_-10)], and one unsaturated proton [*δ*_H_ 5.73 (s, H-3). The ^13^C NMR and HSQC spectrum indicated three ester carbonyls (*δ*_C_ 166.5, 167.0, and 169.8), two olefinic carbons (*δ*_C_ 117.1, and 157.8), three methylenes [*δ*_C_ 39.7, 41.4, and one oxygenated (*δ*_C_ 62.7)], one methoxyl (*δ*_C_ 52.7), and one methyl (*δ*_C_ 19.0).

The ^1^H-^1^H COSY signal of H-5/H-6 with HMBC correlations from H-5 to C-3 and C-4, from H-3 to C-2, from H_3_-11 to C-4, from H-6 and H-8 to C-7, and from H-8 and H_3_-10 to C-9 led to the determination of the planar structure of **8** (Figure 2). The NOESY correlation (Figure 7) of H-3/H-5 manifested that the double bond at 3(4) was an *E* isomer. Therefore, the structure of **8** was established as (*E*)-5-((3-methoxy-3-oxopropanoyl)oxy)-3-methylpent-2-enoic acid.

### 2.2. Antimicrobial Activity Assays

The isolated Compounds **1**–**12** were evaluated for antibacterial activities against methicillin-resistant *Staphylococcus aureus* (MRSA), *Staphylococcus aureus*, *Salmonella typhimurium*, and *Pseudomonas aeruginosa*, and antifungal activities against *Candida albicans*. The antimicrobial activity assays were carried out in 96-well plates by a serial dilution test in the range of 0.1–100 μM. Results showed that the cytochalasins (**1**–**3**, and **10**–**12**) exhibited promising inhibitory activities against the fungi, with a minimal inhibition concentration (MIC) value in the range of 12.5 to 50 μM (Table 5). Wherein, Compound **2** showed moderate antimicrobial activities against *S. aureus* and *C. albicans* with MIC both for 12.5 μM. And Compound **12** displayed moderate inhibitory activity against *P. aeruginosa* with an MIC value of 12.5 μM. No obvious antimicrobial effect was observed under the concentration of 100 μM for Compounds **4**–**9**.

### 2.3. Cytotoxic Activity Assays

The isolated Compounds **1**–**12** were also evaluated for cytotoxic activities against six human cancer cell lines: MDA-MB-435, MDA-MB-231, HCT116, A549, SNB19, and PC-3. Results showed that Compound **10** displayed significant inhibitory activity against MDA-MB-435 with an IC_50_ value of 3.61 ± 1.60 μM. Moreover, the new Compound **1** exhibited moderate cytotoxic activity against SNB19 with an IC_50_ value of 39.18 ± 0.71 μM (Table 6). No obvious cytotoxic effect was observed under the concentration of 50 μM for Compounds **2**–**9**, and **12**. Moreover, no obvious cytotoxic effect was observed under the concentration of 50 μM for Compounds **1** and **10**–**11** toward normal human cell line HLF.

## 3. Materials and Methods

### 3.1. General Experimental Procedures

HRESIMS spectral data were obtained on a ThermoFisher LTQ–Orbitrap–LC–MS spectrometer (Palo Alto, CA, USA). The 1D and 2D NMR spectra were obtained on Bruker Advance 400 MHz and 600 MHZ spectrometer at room temperature. Optical rotations were measured with an MCP300 (Graz, Austria). UV–Vis and ECD curves were acquired using an Applied Photophysics Chirascan spectropolarimeter (Surrey, UK). Silica gel (200–300 mesh, Qingdao Marine Chemical Factory, Qingdao, China) and Sephadex LH-20 (Amersham Pharmacia, Stockholm, Sweden) were used for column chromatography. Semi-preparative HPLC was performed on a Hitachi Primaide 1430 HPLC system combined with a DAD detector (HITACHI, Tokyo, Japan), and a Phenomenex Cellulose-2 column (10 × 250 mm, 5 μm) was applied for separation.

### 3.2. Fungal Material

The endophytic fungus QYF was isolated from the leaf of the mangrove plant *Kandelia candel*, which was collected from Hainan Dongzhai Harbor Mangrove Reserve in 2014. The fungus was identified as *Xylaria arbuscula*. (GenBank accession No. MT123074) based on the analysis of ITS sequence data of the rDNA gene. The strain has been preserved in our laboratory at Sun Yat-sen University, China.

### 3.3. Fermentation, Extraction, and Isolation

The fungus was cultivated on solid cultured medium (sixty 1 L Erlenmeyer flasks, each containing 50 g of rice and 50 mL of 2% saline water) for 30 days at 25 °C. After that, the fermented materials were extracted with EtOAc to yield 60 g of residue. Then, the crude extracts were subjected to silica gel column chromatography using step gradient elution with petroleum ether/ethyl acetate from 9:1 to 0:1 (*v*/*v*) to get seven fractions (Frs.1–Frs.7).

Frs. 6 (20 g) was subjected to Sephadex LH-20 (CH_2_Cl_2_/MeOH, *v*/*v*, 1:1) to yield six subfractions (Frs. 6.1–6.6). Frs. 6.1 (10 g) was applied to silica gel column chromatography (CC) (CH_2_Cl_2_/MeOH, *v*/*v*, 40:1) to give a mixture of **1**, **2**, and **3** (60 mg). The mixture was isolated using reverse phase C18 silica gel column (40–60 μm, MeOH/H_2_O, *v*/*v*, 8:2) to yield **1** (5 mg), **2** (6 mg), and **3** (7 mg). Frs. 4 (7 g) was subjected to Sephadex LH-20 (CH_2_Cl_2_/MeOH, *v*/*v*, 1:1) to yield four subfractions (Frs. 4.1–4.4). Frs. 4.2 (1.2 g) was applied to silica gel CC (CH_2_Cl_2_/MeOH, *v*/*v*, 100:1) to give **5** (2 mg) and **6** (4 mg). Frs. 4.3 (1 g) was applied to silica gel CC (CH_2_Cl_2_/MeOH, *v*/*v*, 50:1) to give **7** (8 mg). Frs. 5 (10 g) was subjected to Sephadex LH-20 (CH_2_Cl_2_/MeOH, *v*/*v*, 1:1) to yield three subfractions (SFrs. 5.1–5.3). Frs. 5.1 (4 g) was applied to silica gel CC (CH_2_Cl_2_/MeOH, *v*/*v*, 100:1) to give a mixture of **4** and **8** (50 mg). The mixture was isolated utilizing HPLC with the Phenomenex Cellulose-2 column, respectively, at t_R_ = 15.6 and 10.2 min (the gradient was MeOH/H_2_O, *v*/*v*, 8:2; flow rate: 1.5 mL/min) to give **4** (4 mg) and **8** (2 mg).

Xylariachalasin A (**1**): C_30_H_37_NO_8_; white powder; ${\left[\alpha \right]}_{D}^{25}$ = +16.4 (c 0.1, MeOH); UV (MeOH) λ_max_ (log ε): 230 (2.90), 270 (2.52) nm; ECD (MeOH) λ_max_ (Δ ε) 230 (+2.86), 290 (−1.67) nm; HRESIMS: *m*/*z* [M + Na]^+^ 562.2435 (calcd. for 562.2411), *m*/*z* [M + H − H_2_O]^+^ 522.2493 (calcd. for 522.2486); ^1^H and ^13^C NMR data (Table 1).

Xylariachalasin B (**2**): C_30_H_37_NO_7_; white powder; ${\left[\alpha \right]}_{D}^{25}$ = +20.5 (c 0.1, MeOH); UV (MeOH) λ_max_ (log ε): 230 (2.88), 270 (2.52) nm; ECD (MeOH) λ_max_ (Δ ε) 230 (+1.89), 290 (−3.38) nm; HRESIMS: *m*/*z* [M + Na]^+^ 546.2456 (calcd. for 546.2462), *m*/*z* [M + H − H_2_O]^+^ 506.2536 (calcd. for 506.2537); ^1^H and ^13^C NMR data (Table 1).

Xylariachalasin C (**3**): C_30_H_35_NO_7_; white powder; ${\left[\alpha \right]}_{D}^{25}$ = +40.0 (c 0.1, MeOH); UV (MeOH) λ_max_ (log ε): 224 (2.11), 270 (1.31) nm; ECD (MeOH) λ_max_ (Δ ε) 226 (+1.32), 287 (−0.47) nm; HRESIMS: *m*/*z* [M + Na]^+^ 544.2309 (calcd. for 544.2306); ^1^H and ^13^C NMR data (Table 2).

Xylariachalasin D (**4**): C_29_H_35_NO_5_; white powder; ${\left[\alpha \right]}_{D}^{25}$ = +13.5 (c 0.1, MeOH); UV (MeOH) λ_max_ (log ε): 225 (2.07), 270 (1.37) nm; ECD (MeOH) λ_max_ (Δ ε) 228 (−1.71), 289 (−0.63) nm; HR-ESI-MS: *m*/*z* [M + Na]^+^ 500.2400 (calcd. for 500.2407); ^1^H and ^13^C NMR data (Table 2).

(*E*)-3-((5-(hydroxymethyl)furan-2-yl)methylene)benzofuran-2(3H)-one (**5**): C_14_H_10_O_4_; yellow oil; HR-ESI-MS: *m*/*z* [M + Na]^+^ 265.0467 (calcd. for 265.0471); ^1^H and ^13^C NMR data (Table 3).

Xylariachromanone A (**6**): C_13_H_14_O_7_; light-yellow oil; ${\left[\alpha \right]}_{D}^{25}$ = +21.4 (c 0.1, MeOH); UV (MeOH) λ_max_ (log ε): 210 (2.31), 222 (2.00), 232 (2.07), 249 (1.66), 269 (1.87), 289 (1.29), 332 (1.86) nm; ECD (MeOH) λ_max_ (Δ ε) 226 (−0.26), 243 (+0.37), 270 (+0.54) nm; HR-ESI-MS: *m*/*z* [M − H]^−^ 281.0665 (calcd. for 281.0667); ^1^H and ^13^C NMR data (Table 3).

Xylariaone A (**7**): C_11_H_16_O_4_; brown oil; ${\left[\alpha \right]}_{D}^{25}$ = −109.5 (c 0.1, MeOH); UV (MeOH) λ_max_ (log ε): 216 (1.58), 254 (2.38) nm; ECD (MeOH) λ_max_ (Δ ε) 215 (+0.36), 252 (−1.27) nm; HR-ESI-MS: *m*/*z* [M + Na]^+^ 235.0936 (calcd. for 235.0941); ^1^H and ^13^C NMR data (Table 4).

(*E*)-5-((3-methoxy-3-oxopropanoyl)oxy)-3-methylpent-2-enoic acid. (**8**): C_10_H_14_O_6_; brown oil; HR-ESI-MS: *m*/*z* [M + Na]^+^ 253.0682 (calcd. for 253.0683); ^1^H and ^13^C NMR data (Table 4).

### 3.4. ECD and Optical Rotation Computation Methods

Initial conformational analysis was carried out by using Spartan 14′ software (Wavefunction Inc., Irvine, CA, USA). The conformers with a Boltzmann distribution larger than 1% were selected for optimization and calculation at B3LYP/6-31 + G(d,p) level in methanol with the density functional theory (DFT) executed by Gaussian 09 [29]. The calculated ECD spectra were extracted and generated by SpecDis 1.6 software (University of Würzburg, Würzburg, Germany).

### 3.5. Antimicrobial Assays

Antimicrobial tests for Compounds **1**–**12** were carried out against pathogenetic strains, including methicillin-resistant *Staphylococcus aureus* (A7983, clinical isolate donated by Zhijun Yu in Dalian Friendship Hospital, Dalian, China), *Staphylococcus aureus* (ATCC 6538), *Salmonella typhimurium* (ATCC 6539), *Pseudomonas aeruginosa* (ATCC 9027), and *Candida albicans* (ATCC 10231). Muller Hinton agar (MHA) and Sabouraud agar (SA) were used for antibacterial and antifungal tests, respectively.

The compounds tested were dissolved individually in dimethyl sulfoxide (DMSO), and the antimicrobial activity assays were carried out in 96-well plates by a serial dilution test in the range of 0.1–100 μM [30,31]. Ampicillin and ketoconazole were used as positive controls for antibacterial and antifungal assays, respectively, and DMSO was utilized as a blank control. The experiments were repeated three times.

### 3.6. Cytotoxic Assays

The cytotoxicities of the isolated Compounds **1**–**8** against six human cancer cell lines— MDA-MB-231 (breast cancer cells), MDA-MB-435 (breast cancer cells), HCT116 (colon cancer cells), A549 (lung cancer cells), SNB19 (glioma cells), PC3 (prostate cancer cells), and normal human cell line HLF (human lung fibroblasts)—were evaluated using the MTT method as previous described. Cisplatin was used as the positive control. 

Human cancer cell lines MDA-MB-231, MDA-MB-435, HCT116, A549, SNB19, PC3 cells, and normal human cell line HLF were obtained from the Shanghai Institute of Biological Sciences (Shanghai, China). Cells were cultured in Dulbecco’s modified Eagle’s medium (DMEM) (Invitrogen, Carls-bad, CA, USA) supplemented with 5% fetal bovine serum (Hyclone, Logan, UT, USA), 2 mM L-glutamine, 100 mg⋅mL^−1^ streptomycin, and 100 units⋅mL^−1^ penicillin (Invitrogen, Carlsbad, CA, USA). The cultures were maintained at 37 °C in a humidified atmosphere of 5% CO_2_. The time of cell cultivation with tested compounds was 36 h.

### 3.7. Preparation of MTPA Esters of 7 by the Modified Mosher Ester Method

Compound **7** (2.0 mg) was reacted with (*S*)-*α*-methoxy-*α*-(trifluoromethyl)-phenylacetyl chloride [(*S*)-MTPA-Cl, 50 μL] in pyridine-*d*_5_ (500 μL) for 12 h at room temperature. Through the same procedure, (*R*)-MTPA-Cl was used for the reaction. The ^1^H NMR and HSQC spectra of the (*R*)- and (*S*)-MTPA esters were recorded. The chemical shifts were assigned based on the HSQC spectrum in order to calculate the Δ*δ_S−R_* values.

(*S*)-MTPA ester for 7a: ^1^ H NMR (pyridine-*d*_5_, 600 MHz) *δ*_H_ 2.92 (H*α*-7), *δ*_H_ 2.95 (H*β*-7), *δ*_H_ 2.68 (H*α*-9), *δ*_H_ 2.98 (H*β*-9).

(*R*)-MTPA ester for 7b: ^1^ H NMR (pyridine-*d*_5_, 600 MHz) *δ*_H_ 2.82 (H*α*-7), *δ*_H_ 2.93 (H*β*-7), *δ*_H_ 2.84 (H*α*-9), *δ*_H_ 3.05 (H*β*-9).

### 3.8. X-ray Crystallographic Analyses

Compounds **10** and **11** were obtained as colorless crystals from a methanol solution evaporated at room temperature. Crystal X-ray diffraction data for **10** and **11** were measured on an Agilent Gemini Ultra diffractometer using graphite-monochromated Cu K*α* radiation (*λ* = 1.54178 Å). The crystallographic data of **10** and **11** have been deposited in the Cambridge Crystallographic Data Centre (CCDC number: 2379563, and 2379564, respectively).

Crystal data of **10**: C_30_H_37_NO_6_, *M_r_* = 507.60, monoclinic, space group: *C2*, *Z* = 4, *a* = 28.9239(5) Å, *b* = 7.38262(13) Å, *c* = 13.1203(3) Å, *α* = *γ* = 90°, *β* = 98.9524(17)°, *V* = 2767.50(9) Å^3^, *D_c_* = 1.218 g/cm^3^, μ = 0.682 mm^−1^, and *F*(000) = 1088.0. Independent reflections: 5592 (*R_int_* = 0.0655, *R_sigma_* = 0.0416). The final *R_1_* value was 0.0476, *wR_2_* = 0.1213 [*I* ≥ 2*σ* (*I*)]. The goodness of fit on *F^2^* was 1.044. The Flack parameter value was 0.14(15).

Crystal data of **11**: C_30_H_37_NO_6_, *M_r_* = 507.60, monoclinic, space group: *C2*, *Z* = 4, *a* = 29.0003(9) Å, *b* = 7.2996(2) Å, *c* = 13.5642(5) *α* = *γ* = 90°, *β* = 98.693(3)°, *V* = 2838.45(16) Å^3^, *D_c_* = 1.188 g/cm^3^, μ = 0.665 mm^−1^, and *F*(000) = 1088.0. Independent reflections: 5538 (*R_int_* = 0.0857, *R_sigma_* = 0.0672). The final *R_1_* value was 0.0571, *wR_2_* = 0.1349 [*I* ≥ 2*σ* (*I*)]. The goodness of fit on *F^2^* was 1.039. The Flack parameter value was −0.1(2).

## 4. Conclusions

In conclusion, twelve compounds, including four undescribed cytochalasins, xylariachalasins A–D (**1**–**4**), four undescribed polyketides (**5**–**8**), and four known cytochalasins (**9**–**12**), were isolated from the mangrove endophytic fungus *Xylaria arbuscula* QYF. The planar structure of compounds was elucidated through detailed HRESIMS and 1D and 2D NMR analysis. The absolute configurations of the new compounds were confirmed by ECD calculation, ^13^C NMR calculation and DP4+ analysis, single-crystal X-ray diffraction, and the modified Mosher ester method. Compounds **1** and **2** are rare cytochalasin hydroperoxides. In bioassays, antimicrobial and cytotoxic activities were carried out for compounds **1**–**12**. Compound **2** showed moderate antimicrobial activities against MRSA, *S. aureus*, *P. aeruginosa*, and *C. albicans*, with MIC values in the range of 12.5–25 μM. Compound **10** exhibited significant cytotoxic activity against MDA-MB-435 with an IC_50_ value of 3.61 ± 1.60 μM.

## Figures and Tables

**Table 1:** ^1^H and ^13^C NMR data of **1** and **2** (*δ* ppm).

Position	1 ^a^		2 ^b^	
	*δ*_C_, Type	*δ*_H_ (*J* in Hz)	*δ*_C_, Type	*δ*_H_ (*J* in Hz)
1	176.7, C		174.6, C	
3	61.8, CH	3.36, m	60.6, CH	3.35, m
4	50.3, CH	2.52, br.s	50.3, CH	2.50, overlapped
5	134.9, C		128.9, C	
6	137.0, C		137.6, C	
7	83.0, CH	4.07, d (10.0)	83.2, CH	4.11, d (10.2)
8	44.8, CH	3.09, t (10.2)	44.6, CH	3.00, overlapped
9	54.4, C		53.3, C	
10	44.7, CH_2_	*α*: 2.85, dd (13.2, 9.9) *β:* 3.06, dd (13.2, 5.3)	44.4, CH_2_	2.98, overlapped
11	16.7, CH_3_	1.19, s	17.4, CH_3_	1.46, s
12	58.7, CH_2_	*α*: 4.04, d (11.5) *β:* 4.22, d (11.5)	14.2, CH_3_	1.75, s
13	131.0, CH	5.69, dd (15.5, 10.0)	132.3, CH	6.01, dd (15.7, 10.4)
14	134.1, CH	5.34, ddd (15.4, 10.7, 5.0)	131.3, CH	5.95, overlapped
15	39.6, CH_2_	*α*: 2.04, m *β:* 2.40, m	38.3, CH_2_	*α*: 2.02, m *β:* 2.52, overlapped
16	43.4, CH	2.85, m	42.3, CH	2.72, m
17	211.6, C		210.2, C	
18	79.4, C		77.8, C	
19	129.4, CH	5.29, dd (15.4, 2.0)	127.9, CH	5.16, dd (15.7, 2.4)
20	133.0, CH	5.96, overlapped	131.9, CH	5.34, m
21	76.5, CH	5.91, overlapped	75.2, CH	5.94, overlapped
22	19.8, CH_3_	1.15, d (6.8)	19.5, CH_3_	1.21, d (6.8)
23	24.6, CH_3_	1.50, s	24.4, CH_3_	1.52, s
24	172.1, C		170.2, C	
25	20.7, CH_3_	2.33, s	21.1, CH_3_	2.32, s
1′	138.9, C		137.6, C	
2′/6′	129.8, CH	7.32, d (7.0)	129.0, CH	7.32, d (7.7)
3′/5′	130.7, CH	7.25, m	129.2, CH	7.19, m
4′	128.0, CH	7.26, m	127.2, CH	7.25, m

^a 1^H (400 MHz) and ^13^C (100 MHz) NMR data in CD_3_OD. ^b 1^H (400 MHz) and ^13^C (100 MHz) NMR data in CDCl_3_.

**Table 2:** ^1^H and ^13^C NMR data of **3** and **4** (*δ* ppm).

Position	3 ^a^		4 ^b^	
	*δ*_C_, Type	*δ*_H_ (*J* in Hz)	*δ*_C_, Type	*δ*_H_ (*J* in Hz)
1	175.1, C		177.1, C	
3	61.2, CH	3.49, m	62.1, CH	3.29, m
4	50.7, CH	2.91, m	50.2, CH	2.45, m
5	154.5, C		129.6, C	
6	136.3, C		131.2, C	
7	199.7, C		70.1, CH	3.72, d (10.4)
8	53.2, CH	3.58, d (9.7)	43.3, CH	2.84, overlapped
9	54.2, C		50.6, C	
10	44.9, CH_2_	*α*: 2.98, dd (13.2, 9.9) *β:* 3.14, dd (13.2, 5.2)	45.0, CH_2_	*α*: 2.85, overlapped *β:* 3.00, dd (13.2, 5.4)
11	18.0, CH_3_	1.40, s	17.2, CH_3_	1.04, s
12	56.0, CH_2_	*α*: 4.04, d (11.5) *β:* 4.25, d (11.5)	14.4, CH_3_	1.61, s
13	130.4, CH	5.35, dd (15.7, 10.4)	132.1, CH	5.82, overlapped
14	128.2, CH	5.86, overlapped	134.8, CH	5.19, m
15	39.4, CH_2_	*α*: 2.02, m *β:* 2.42, m	38.7, CH_2_	*α*: 1.97, m *β:* 2.32, m
16	43.3, CH	2.87, m	47.8, CH	2.65, m
17	211.9, C		210.4, C	
18	79.5, C		45.9, CH_2_	*α*: 2.88, overlapped *β:* 3.26, m
19	131.7, CH	5.81, overlapped	117.9, CH	5.25, m
20	135.1, CH	5.21, m	135.3, CH	5.85, m
21	75.9, CH	5.98, t (2.4)	76.8, CH	5.81, overlapped
22	19.6, CH_3_	1.16, d (6.8)	18.9, CH_3_	1.08, d (6.9)
23	24.6, CH_3_	1.49, s	172.2, C	
24	171.9, C		20.7, CH_3_	2.27, s
25	20.7, CH_3_	2.36, s		
1′	138.5, C		139.0, C	
2′/6′	130.0, CH	7.35, d (7.5)	129.7, CH	7.30, d (7.6)
3′/5′	130.7, CH	7.27, m	130.7, CH	7.23, m
4′	128.3, CH	7.29, m	127.9, CH	7.22, m

^a 1^H (400 MHz) and ^13^C (100 MHz) NMR data in CD_3_OD. ^b 1^H (600 MHz) and ^13^C (150 MHz) NMR data in CD_3_OD.

**Table 3:** ^1^H (600 MHz) and ^13^C (150 MHz) NMR data of **5** and **6** (*δ* ppm) in CD_3_OD.

Position	5		6	
	*δ*_C_, Type	*δ*_H_ (*J* in Hz)	*δ*_C_, Type	*δ*_H_ (*J* in Hz)
2	171.4, C		169.7, C	
3	117.3, C		98.0, C	
4	123.6, C		159.1, C	
5	126.4, CH	8.58, d (8.0)	104.0, CH	6.41, s
6	125.3, CH	7.26, t (8.0)	156.1, C	
7	131.4, CH	7.40, t (8.0)	139.4, C	
8	111.3, CH	7.17, overlapped	121.4, C	
9	155.4, C		70.8, CH	5.12, s
10	124.4, CH	7.47, s	113.8, C	
11	152.0, C		43.5, CH_2_	*α*: 2.08, dd (12.8, 9.5) *β:* 2.73, dd (12.8, 6.2)
12	124.1, CH	7.17, overlapped	76.3, CH	4.39, m
13	112.2, CH	6.65, d (3.4)	53.5, CH_3_	3.50, s
14	162.0, C		22.1, CH_3_	1.38, d (6.2)
15	57.8, CH_2_	4.74, s		

**Table 4:** ^1^H and ^13^C NMR data of **7** and **8** (*δ* ppm).

Position	7 ^a^		8 ^b^	
	*δ*_C_, Type	*δ*_H_ (*J* in Hz)	*δ*_C_, Type	*δ*_H_ (*J* in Hz)
2	72.0, CH	4.31, m	169.8, C	
3	34.1, CH_2_	*α*: 1.38, m *β:* 2.11, m	117.1, CH	5.73, s
4	67.1, CH	4.19, br.s	157.8, C	
5	113.3, C		39.7, CH_2_	2.51, t (6.5)
6	197.9, C		62.7, CH_2_	4.31, t (6.5)
7	46.7, CH_2_	*α*: 2.45, dd (15.8, 9.0) *β:* 2.60, dd (15.8, 4.4)	167.0, C	
8	65.5, CH	4.15, m	41.4, CH_2_	3.38, s
9	38.7, CH_2_	*α*: 2.48, dd (17.4, 8.4) *β:* 2.72, dd (17.4, 5.0)	166.5, C	
10	174.0, C		52.7, CH_3_	3.74, s
11	56.7, CH_3_	3.35, s	19.0, CH_3_	2.20, s
12	20.8, CH_3_	1.40, d (6.4)		

^a 1^H (600 MHz) and ^13^C (150 MHz) NMR data in CD_3_OD. ^b 1^H (600 MHz) and ^13^C (150 MHz) NMR data in CDCl_3_.

**Table 5:** MIC for antibacterial and antifungal activities of compounds.

	MIC of Compounds/μM							
	1	2	3	10	11	12	Amp. ^1^	Ket. ^2^
MRSA	>100	25	50	>100	50	50	0.25	NT
*S. aureus*	>100	12.5	>100	>100	>100	>100	0.25	NT
*S. typhimurium*	>100	>100	25	>100	25	>100	0.25	NT
*P. aeruginosa*	>100	25	>100	>100	>100	12.5	0.13	NT
*C. albicans*	25	12.5	>100	25	50	50	NT	0.13

^1^ Ampicillin, positive control toward bacteria. ^2^ Ketoconazole, positive control toward fungi.

**Table 6:** Cytotoxic activities of compounds (IC_5_ ± SD, μM).

Compound	MDA-MB-435	MDA-MB-231	HCT116	A549	SNB19	PC3
**1**	>50	>50	>50	>50	39.18 ± 0.71	>50
**10**	3.61 ± 1.60	>50	>50	>50	23.76 ± 0.54	>50
**11**	>50	>50	>50	20.11 ± 2.71	>50	22.92 ± 0.43
Cisplatin ^1^	44.43 ± 3.25	38.16 ± 5.93	14.68 ± 1.34	26.42 ± 7.58	26.54 ± 1.53	33.00 ± 0.19

^1^ Cisplatin, positive control.

## Data Availability

Data are contained within this article and Supplementary Materials.

## Supplementary Materials

The following supporting information can be downloaded at https://www.mdpi.com/article/10.3390/md22090407/s1, Figure S1. HRESIMS spectrum of **1** (*m*/*z* [M + Na]^+^). Figure S2. HRESIMS spectrum of **1** (*m*/*z* [M + H - H_2_O]^+^). Figure S3. ^1^H NMR spectrum of **1** in CD_3_OD. Figure S4. ^13^C NMR spectrum of **1** in CD_3_OD. Figure S5. HSQC spectrum of **1** in CD_3_OD. Figure S6. COSY spectrum of **1** in CD_3_OD. Figure S7. HMBC spectrum of **1** in CD_3_OD. Figure S8. NOESY spectrum of **1** in CD_3_OD. Figure S9. UV spectrum of **1**. Figure S10. CD spectrum of **1**. Figure S11. HRESIMS spectrum of **2** (*m*/*z* [M + Na]^+^). Figure S12. HRESIMS spectrum of **2** (*m*/*z* [M + H - H_2_O]^+^). Figure S13. ^1^H NMR spectrum of **2** in CDCl_3_. Figure S14. ^13^C NMR spectrum of **2** in CDCl_3_. Figure S15. HSQC spectrum of **2** in CDCl_3_. Figure S16. COSY spectrum of **2** in CDCl_3_. Figure S17. HMBC spectrum of **2** in CDCl_3_. Figure S18. NOESY spectrum of **2** in CDCl_3_**.** Figure S19. UV spectrum of **2**. Figure S20. CD spectrum of **2**. Figure S21. HRESIMS spectrum of **3**. Figure S22. ^1^H NMR spectrum of **3** in CD_3_OD. Figure S23. ^13^C NMR spectrum of **3** in CD_3_OD. Figure S24. HSQC spectrum of **3** in CD_3_OD. Figure S25. COSY spectrum of **3** in CD_3_OD. Figure S26. HMBC spectrum of **3** in CD_3_OD. Figure S27. NOESY spectrum of **3** in CD_3_OD. Figure S28. UV spectrum of **3**. Figure S29. CD spectrum of **3**. Figure S30. HRESIMS spectrum of **4**. Figure S31. ^1^H NMR spectrum of **4** in CD_3_OD. Figure S32. ^13^C NMR spectrum of **4** in CD_3_OD. Figure S33. HSQC spectrum of **4** in CD_3_OD. Figure S34. COSY spectrum of **4** in CD_3_OD. Figure S35. HMBC spectrum of **4** in CD_3_OD. Figure S36. NOESY spectrum of **4** in CD_3_OD. Figure S37. UV spectrum of **4**. Figure S38. CD spectrum of **4**. Figure S39. HRESIMS spectrum of **5**. Figure S40. ^1^H NMR spectrum of **5** in CD_3_OD. Figure S41. ^13^C NMR spectrum of **5** in CD_3_OD. Figure S42. HSQC spectrum of **5** in CD_3_OD. Figure S43. COSY spectrum of **5** in CD_3_OD. Figure S44. HMBC spectrum of **5** in CD_3_OD. Figure S45. NOESY spectrum of **5** in CD_3_OD. Figure S46. HRESIMS spectrum of **6**. Figure S47. ^1^H NMR spectrum of **6** in CD_3_OD. Figure S48. ^13^C NMR spectrum of **6** in CD_3_OD. Figure S49. HSQC spectrum of **6** in CD_3_OD. Figure S50. COSY spectrum of **6** in CD_3_OD. Figure S51. HMBC spectrum of **6** in CD_3_OD. Figure S52. NOESY spectrum of **6** in CD_3_OD. Figure S53. UV spectrum of **6**. Figure S54. CD spectrum of **6**. Figure S55. HRESIMS spectrum of **7**. Figure S56. ^1^H NMR spectrum of **7** in CD_3_OD. Figure S57. ^13^C NMR spectrum of **7** in CD_3_OD. Figure S58. HSQC spectrum of **7** in CD_3_OD. Figure S59. COSY spectrum of **7** in CD_3_OD. Figure S60. HMBC spectrum of **7** in CD_3_OD. Figure S61. NOESY spectrum of **7** in CD_3_OD. Figure S62. UV spectrum of **7**. Figure S63. CD spectrum of **7**. Figure S64. HRESIMS spectrum of **8**. Figure S65. ^1^H NMR spectrum of **8** in CDCl_3_. Figure S66. ^13^C NMR spectrum of **8** in CDCl_3_. Figure S67. HSQC spectrum of **8** in CDCl_3_. Figure S68. COSY spectrum of **8** in CDCl_3_. Figure S69. HMBC spectrum of **8** in CDCl_3_. Figure S70. NOESY spectrum of **8** in CDCl_3_. Figure S71. HRESIMS spectrum of **9** (*m*/*z* [M + Na]^+^). Figure S72. ^1^H NMR spectrum of **9** in DMSO-*d_6_*. Figure S73. ^13^C NMR spectrum of **9** in DMSO- *d_6_*. Figure S74. ^1^H NMR spectrum of (*R*)-MTPA ester of **7**. Figure S75. HSQC spectrum of (*R*)-MTPA ester of **7**. Figure S76. ^1^H NMR spectrum of (*S*)-MTPA ester of **7**. Figure S77. HSQC spectrum of (*S*)-MTPA ester of **7**. Figure S78. Comparison of the experimental ^13^C NMR data of compound **1** and the calculated chemical shifts of (3*S,* 4*R*, 7S, 8*R*, 9*R*, 16*S*, 18*R*, 21*R*-**1**, and 3*S,* 4*R*, 7S, 8*R*, 9*R*, 16*S*, 18*R*, 21*S*-**1**). Figure S79. DP4+ analysis of compound **1**. Figure S80. ^13^C NMR spectrum of **11** in CD_3_OD. Table S1. Gibbs free energy and Boltzmann population of low energy of 3*S,* 4*R*, 7S, 8*R*, 9*R*, 16*S*, 18*R*, 21*R* -**1** in MeOH. Table S2. Gibbs free energy and Boltzmann population of low energy of 3*S,* 4*R*, 8*R*, 9*R*, 16*S*, 18*R*, 21*R* -**3** in MeOH. Table S3. Gibbs free energy and Boltzmann population of low energy of 3*S,* 4*R*, 8*R*, 9*R*, 16*S*, 18*R*, 21*R* -**4** in MeOH. Table S4. Gibbs free energy and Boltzmann population of low energy of 9*S*, 10*S*, 12*S*-**6** in MeOH. Table S5. Gibbs free energy and Boltzmann population of low energy of 2*S*, 4*S*, 8*S* -**7** in MeOH. Table S6. Gibbs free energy and Boltzmann population of low energy of 2*R*, 4*R*, 8*S* -**7** in MeOH. Table S7. Gibbs free energy and Boltzmann population of low energy of 2*S*, 4*R*, 8*S* -**7** in MeOH. Table S8. Gibbs free energy and Boltzmann population of low energy of 2*R*, 4*S*, 8*S* -**7** in MeOH.
