# Supplementary material for: The Cytochalasins and Polyketides from a Mangrove Endophytic Fungus Xylaria arbuscula QYF
Source: Mar Drugs. 2024 Sep 5;22(9):407. doi: 10.3390/md22090407 (PMC11433540; doi:10.3390/md22090407)

checkCIF/PLATON report

Structure factors have been supplied for datablock(s) a24081203qy-1

THIS REPORT IS FOR GUIDANCE ONLY. IF USED AS PART OF A REVIEW PROCEDURE FOR PUBLICATION, IT SHOULD NOT REPLACE THE EXPERTISE OF AN EXPERIENCED CRYSTALLOGRAPHIC REFEREE.

No syntax errors found. CIF dictionary Interpreting this report

Datablock: a24081203qy-1

```
Bond precision:   C-C = 0.0046 A          Wavelength=1.54184

Cell:            a=28.9239(5)    b=7.38262(13)        c=13.1203(3)
                 alpha=90        beta=98.9524(17)     gamma=90
Temperature:     150 K
```

	Calculated	Reported
Volume	2767.50(9)	2767.50(9)
Space group	C 2	C 1 2 1
Hall group	C 2y	C 2y
Moiety formula	C30 H37 N O6	C30 H37 N O6
Sum formula	C30 H37 N O6	C30 H37 N O6
Mr	507.61	507.60
Dx,g cm-3	1.218	1.218
Z	4	4
Mu (mm-1)	0.682	0.682
F000	1088.0	1088.0
F000’	1091.34	
h,k,lmax	36,9,16	35,9,16
Nref	5801[3135]	5591
Tmin,Tmax	0.995,0.997	0.709,1.000
Tmin’	0.949	

```
Correction method= # Reported T Limits: Tmin=0.709 Tmax=1.000
AbsCorr = MULTI-SCAN

Data completeness= 1.78/0.96       Theta(max)= 76.136

R(reflections)= 0.0475( 5107)              wR2(reflections)=
                                           0.1264( 5591)
S = 1.032                    Npar= 340
```

The following ALERTS were generated. Each ALERT has the format
      test-name_ALERT_alert-type_alert-level.
Click on the hyperlinks for more details of the test.

Alert level C

```
PLAT340_ALERT_3_C Low Bond Precision on  C-C Bonds ...............    0.00458 Ang.
PLAT911_ALERT_3_C Missing FCF Refl Between Thmin & STh/L=    0.600          3 Report
               -4   0   6,    4   0   6,   28   0   7,
```

Alert level G

```
PLAT007_ALERT_5_G Number of Unrefined Donor-H Atoms ..............          3 Report
              H1     H2     H4
PLAT480_ALERT_4_G Long H...A H-Bond Reported H22      ..O3       .       2.70 Ang.
PLAT791_ALERT_4_G Model has Chirality at C2         (Sohncke SpGr)          R Verify
PLAT791_ALERT_4_G Model has Chirality at C3         (Sohncke SpGr)          R Verify
PLAT791_ALERT_4_G Model has Chirality at C4         (Sohncke SpGr)          S Verify
PLAT791_ALERT_4_G Model has Chirality at C5         (Sohncke SpGr)          S Verify
PLAT791_ALERT_4_G Model has Chirality at C7         (Sohncke SpGr)          S Verify
PLAT791_ALERT_4_G Model has Chirality at C8         (Sohncke SpGr)          R Verify
PLAT791_ALERT_4_G Model has Chirality at C12        (Sohncke SpGr)          S Verify
PLAT791_ALERT_4_G Model has Chirality at C14        (Sohncke SpGr)          R Verify
PLAT791_ALERT_4_G Model has Chirality at C17        (Sohncke SpGr)          R Verify
PLAT912_ALERT_4_G Missing # of FCF Reflections Above STh/L=  0.600         71 Note
PLAT933_ALERT_2_G Number of HKL-OMIT Records in Embedded .res File         10 Note
              -10 -2   3,   -4   0   6,   -2 -4   4,    4   0   6,    7   5   7,   13   1   9,
               17 -1   6,   22 -2   7,   27   1   8,   29   1   6,
PLAT969_ALERT_5_G The 'Henn et al.' R-Factor-gap value ..........      2.766 Note
              Predicted wR2: Based on SigI**2  4.57 or SHELX Weight 12.25
PLAT978_ALERT_2_G Number C-C Bonds with Positive Residual Density.          7 Info
```

   0 **ALERT level A** = Most likely a serious problem - resolve or explain
   0 **ALERT level B** = A potentially serious problem, consider carefully
   2 **ALERT level C** = Check. Ensure it is not caused by an omission or oversight
  15 **ALERT level G** = General information/check it is not something unexpected

```
   0 ALERT type 1 CIF construction/syntax error, inconsistent or missing data
   2 ALERT type 2 Indicator that the structure model may be wrong or deficient
   2 ALERT type 3 Indicator that the structure quality may be low
  11 ALERT type 4 Improvement, methodology, query or suggestion
   2 ALERT type 5 Informative message, check
```

It is advisable to attempt to resolve as many as possible of the alerts in all categories. Often the minor alerts point to easily fixed oversights, errors and omissions in your CIF or refinement strategy, so attention to these fine details can be worthwhile. In order to resolve some of the more serious problems it may be necessary to carry out additional measurements or structure refinements. However, the purpose of your study may justify the reported deviations and the more serious of these should normally be commented upon in the discussion or experimental section of a paper or in the "special_details" fields of the CIF. checkCIF was carefully designed to identify outliers and unusual parameters, but every test has its limitations and alerts that are not important in a particular case may appear. Conversely, the absence of alerts does not guarantee there are no aspects of the results needing attention. It is up to the individual to critically assess their own results and, if necessary, seek expert advice.

Publication of your CIF in IUCr journals

A basic structural check has been run on your CIF. These basic checks will be run on all CIFs submitted for publication in IUCr journals (*Acta Crystallographica*, *Journal of Applied Crystallography*, *Journal of Synchrotron Radiation*); however, if you intend to submit to *Acta Crystallographica Section C* or *E* or *IUCrData*, you should make sure that full publication checks are run on the final version of your CIF prior to submission.

Publication of your CIF in other journals

Please refer to the *Notes for Authors* of the relevant journal for any special instructions relating to CIF submission.

PLATON version of 15/07/2024; check.def file version of 15/07/2024

Datablock a24081203qy-1 - ellipsoid plot

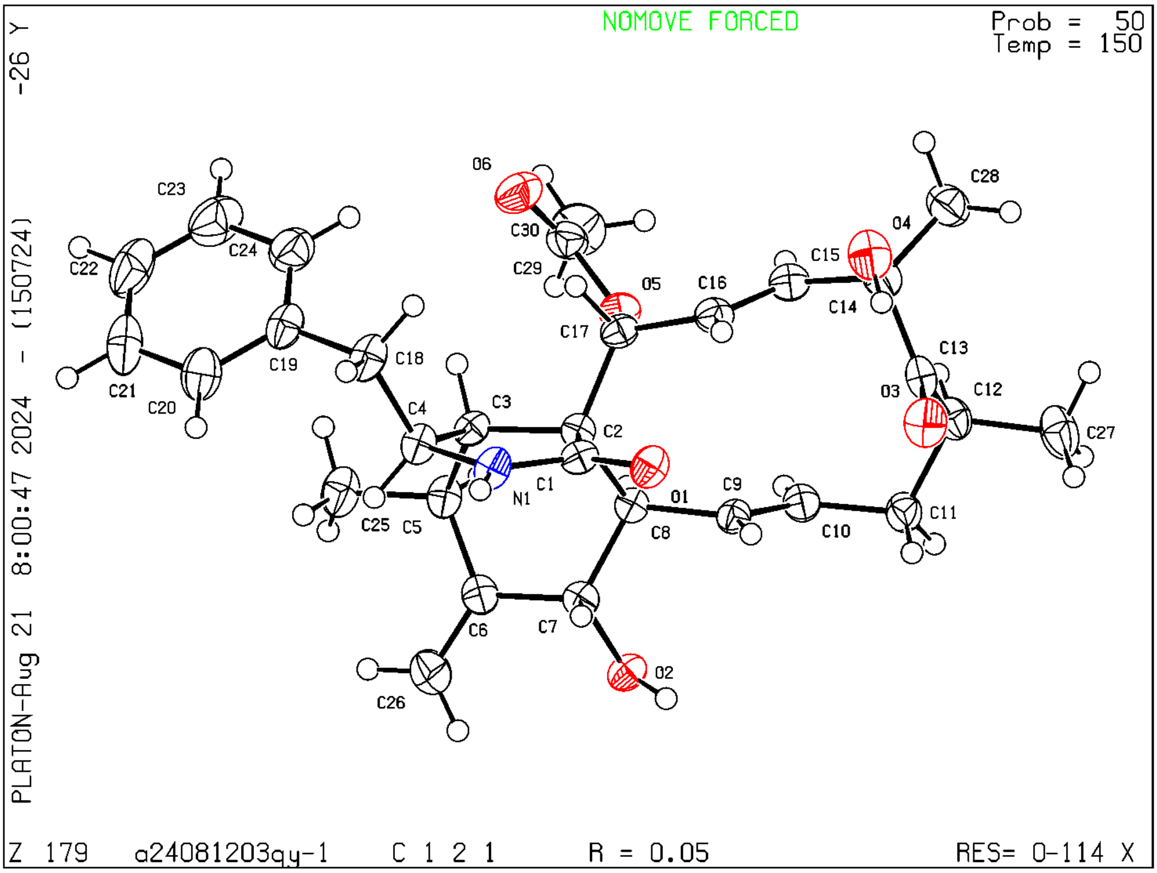

Supplement: Supplementary file 1 [file marinedrugs-22-00407-s001.zip › compound 10_cifreport.pdf]
