# Supplementary material for: The Cytochalasins and Polyketides from a Mangrove Endophytic Fungus Xylaria arbuscula QYF
Source: Mar Drugs. 2024 Sep 5;22(9):407. doi: 10.3390/md22090407 (PMC11433540; doi:10.3390/md22090407)

checkCIF/PLATON report

Structure factors have been supplied for datablock(s) a24081501qy-2_auto

THIS REPORT IS FOR GUIDANCE ONLY. IF USED AS PART OF A REVIEW PROCEDURE FOR PUBLICATION, IT SHOULD NOT REPLACE THE EXPERTISE OF AN EXPERIENCED CRYSTALLOGRAPHIC REFEREE.

No syntax errors found. CIF dictionary Interpreting this report

Datablock: a24081501qy-2_auto

Bond precision:	C-C = 0.0065 A		Wavelength=1.54184
Cell:	a=29.0003(9)	b=7.2996(2)	c=13.5642(5)
	alpha=90	beta=98.693(3)	gamma=90
Temperature:	150 K		

	Calculated	Reported
Volume	2838.43(16)	2838.45(16)
Space group	C 2	C 1 2 1
Hall group	C 2y	C 2y
Moiety formula	C30 H37 N O6	C30 H37 N O6
Sum formula	C30 H37 N O6	C30 H37 N O6
Mr	507.61	507.60
Dx,g cm-3	1.188	1.188
Z	4	4
Mu (mm-1)	0.665	0.665
F000	1088.0	1088.0
F000’	1091.34	
h,k,lmax	35,9,16	35,9,16
Nref	5669[3068]	5538
Tmin,Tmax	0.995,0.997	0.799,1.000
Tmin’	0.982	

Correction method= # Reported T Limits: Tmin=0.799 Tmax=1.000
AbsCorr = MULTI-SCAN

Data completeness= 1.81/0.98 Theta(max)= 72.987

R(reflections)= 0.0571(4574) wR2(reflections)= 0.1453(5538)

S = 1.039 Npar= 344

The following ALERTS were generated. Each ALERT has the format
test-name_ALERT_alert-type_alert-level.
Click on the hyperlinks for more details of the test.

Alert level C

```
PLAT220_ALERT_2_C NonSolvent   Resd 1  C   Ueq(max)/Ueq(min) Range        3.6 Ratio
PLAT241_ALERT_2_C High   'MainMol' Ueq as Compared to Neighbors of        C21 Check
PLAT242_ALERT_2_C Low    'MainMol' Ueq as Compared to Neighbors of        C19 Check
PLAT245_ALERT_2_C U(iso) H4         Smaller than U(eq) O4        by      0.016 Ang**2
PLAT331_ALERT_2_C Small Aver Phenyl C-C Dist C19      --C24      .       1.37 Ang.
PLAT340_ALERT_3_C Low Bond Precision on  C-C Bonds ...............    0.00645 Ang.
PLAT601_ALERT_2_C Unit Cell Contains Solvent Accessible VOIDS of .         57 Ang**3
PLAT911_ALERT_3_C Missing FCF Refl Between Thmin & STh/L=    0.600         17 Report
              -24  0  3, -21  1  3, -21  3  3, -10  2  3,  -8  2  3,  -6  2  3,
               -4  0  3,  -2  4  3,   0  0  3,   4  0  3,   5  1  3,   9  1  3,
               -4  0  6,  -4  4  6,   4  0  6,   5  1  6,   6  0  6,
```

Alert level G

```
PLAT007_ALERT_5_G Number of Unrefined Donor-H Atoms ..............          2 Report
              H1    H2
PLAT791_ALERT_4_G Model has Chirality at C2          (Sohncke SpGr)          R Verify
PLAT791_ALERT_4_G Model has Chirality at C3          (Sohncke SpGr)          R Verify
PLAT791_ALERT_4_G Model has Chirality at C4          (Sohncke SpGr)          S Verify
PLAT791_ALERT_4_G Model has Chirality at C7          (Sohncke SpGr)          S Verify
PLAT791_ALERT_4_G Model has Chirality at C8          (Sohncke SpGr)          R Verify
PLAT791_ALERT_4_G Model has Chirality at C12         (Sohncke SpGr)          S Verify
PLAT791_ALERT_4_G Model has Chirality at C14         (Sohncke SpGr)          R Verify
PLAT791_ALERT_4_G Model has Chirality at C17         (Sohncke SpGr)          R Verify
PLAT912_ALERT_4_G Missing # of FCF Reflections Above STh/L=  0.600         14 Note
PLAT933_ALERT_2_G Number of HKL-OMIT Records in Embedded .res File         44 Note
              -24  0  3, -24  2  3, -21 -3  3, -21 -1  3, -21  1  3, -21  3  3,
              -14 -6  1, -12  2  3, -10 -2  3, -10  2  3,  -9 -3  3,  -8 -2  3,
               -8  2  3,  -6 -2  3,  -6  2  3,  -5 -7  9,  -5 -5 14,  -4 -4  6,
               -4  0  3,  -4  0  6,  -4  4  6,  -2 -4  3,  -2  4  3,  -1 -5  6,
                0 -2  3,   0  0  3,   1  1  6,   1  5  3,   3 -7 10,   3  3  3,
                4 -4 12,   4  0  3,   4  0  6,   5 -7  7,   5 -1  3,   5 -1  6,
                5  1  3,   5  1  6,   6 -6  7,   6  0  6,   7  1  3,   9 -1  3,
                9  1  3,  13 -5  1,
PLAT969_ALERT_5_G The 'Henn et al.' R-Factor-gap value ...........      2.115 Note
              Predicted wR2: Based on SigI**2  6.87 or SHELX Weight 13.99
PLAT978_ALERT_2_G Number C-C Bonds with Positive Residual Density.          2 Info
```

0 **ALERT level A** = Most likely a serious problem - resolve or explain
0 **ALERT level B** = A potentially serious problem, consider carefully
8 **ALERT level C** = Check. Ensure it is not caused by an omission or oversight
13 **ALERT level G** = General information/check it is not something unexpected

0 ALERT type 1 CIF construction/syntax error, inconsistent or missing data
8 ALERT type 2 Indicator that the structure model may be wrong or deficient
2 ALERT type 3 Indicator that the structure quality may be low
9 ALERT type 4 Improvement, methodology, query or suggestion
2 ALERT type 5 Informative message, check

It is advisable to attempt to resolve as many as possible of the alerts in all categories. Often the minor alerts point to easily fixed oversights, errors and omissions in your CIF or refinement strategy, so attention to these fine details can be worthwhile. In order to resolve some of the more serious problems it may be necessary to carry out additional measurements or structure refinements. However, the purpose of your study may justify the reported deviations and the more serious of these should normally be commented upon in the discussion or experimental section of a paper or in the "special_details" fields of the CIF. checkCIF was carefully designed to identify outliers and unusual parameters, but every test has its limitations and alerts that are not important in a particular case may appear. Conversely, the absence of alerts does not guarantee there are no aspects of the results needing attention. It is up to the individual to critically assess their own results and, if necessary, seek expert advice.

Publication of your CIF in IUCr journals

A basic structural check has been run on your CIF. These basic checks will be run on all CIFs submitted for publication in IUCr journals (*Acta Crystallographica*, *Journal of Applied Crystallography*, *Journal of Synchrotron Radiation*); however, if you intend to submit to *Acta Crystallographica Section C* or *E* or *IUCrData*, you should make sure that full publication checks are run on the final version of your CIF prior to submission.

Publication of your CIF in other journals

Please refer to the *Notes for Authors* of the relevant journal for any special instructions relating to CIF submission.

PLATON version of 15/07/2024; check.def file version of 15/07/2024

Datablock a24081501qy-2_auto - ellipsoid plot

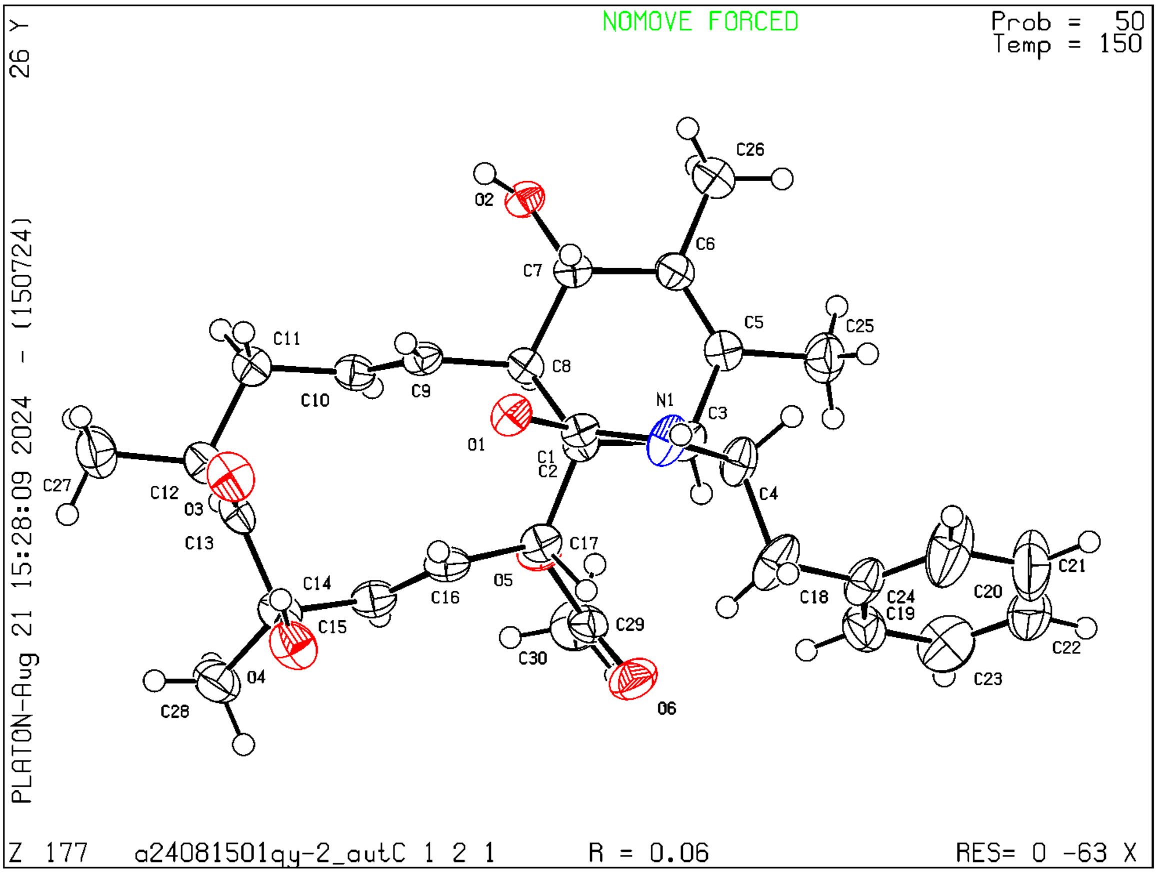

Supplement: Supplementary file 1 [file marinedrugs-22-00407-s001.zip › compound 11 cifreport.pdf]
