# Supplementary material for: The Cytochalasins and Polyketides from a Mangrove Endophytic Fungus Xylaria arbuscula QYF
Source: Mar Drugs. 2024 Sep 5;22(9):407. doi: 10.3390/md22090407 (PMC11433540; doi:10.3390/md22090407)
Supplement: Supplementary file 1 [file marinedrugs-22-00407-s001.zip › supporting information-revised.pdf]

Supporting Information

The Cytochalasins and Polyketides from a Mangrove Endophytic Fungus *Xylaria arbuscula* QYF

Qi Tan [1], Xinyu Ye [1], Siqi Fu [2], Yihao Yin [1], Yufeng Liu [1], Jianying Wu [1], Fei Cao [2], Bo Wang [1], Tingshun Zhu [1], Wencong Yang [1, 3, *], and Zhigang She [1, *]

[1] School of Chemistry, Sun Yat-sen University, Guangzhou, 510006, China; tanq27@mail2.sysu.edu.cn (Q.T.); yexy55@mail3.sysu.edu.cn (X.Y.); yinyh6@mail2.sysu.edu.cn (Y.Y.); liuyf76@mail2.sysu.edu.cn (Y.L.); wujy89@mail2.sysu.edu.cn (J.W.); ceswb@mail.sysu.edu.cn (B.W.); zhutshun@mail.sysu.edu.cn (T.Z.)

[2] College of Pharmaceutical Sciences, Hebei University, Baoding, 071002, China; 15703382700@163.com (S.F.); caofei542927001@163.com (F.C.)

[3] School of Pharmaceutical Sciences, Sun Yat-sen University, Guangzhou, 510006, China

***** Correspondence: yangwc6@mail2.sysu.edu.cn (W.Y.); cesshzhg@mail.sysu.edu.cn (Z.S.)

Content

Figure S1. HRESIMS spectrum of **1** **(***m*/*z* [M + Na]$^+$**)**. 6

Figure S2. HRESIMS spectrum of **1** **(***m*/*z* [M + H - H_2O]$^+$**)**. 6

Figure S3. ^{1}H NMR spectrum of **1** in CD_3OD. 7

Figure S4. ^{13}C NMR spectrum of **1** in CD_3OD. 7

Figure S5. HSQC spectrum of **1** in CD_3OD. 8

Figure S6. COSY spectrum of **1** in CD_3OD. 8

Figure S7. HMBC spectrum of **1** in CD_3OD. 9

Figure S8. NOESY spectrum of **1** in CD_3OD. 9

Figure S9. UV spectrum of **1**. 10

Figure S10. CD spectrum of **1**. 10

Figure S11. HRESIMS spectrum of **2** **(***m*/*z* [M + Na]$^+$**)**. 10

Figure S12. HRESIMS spectrum of **2** **(***m*/*z* [M + H - H_2O]$^+$**)**. 11

Figure S13. ^{1}H NMR spectrum of **2** in $CDCl_3$. 11

Figure S14. ^{13}C NMR spectrum of **2** in $CDCl_3$. 12

Figure S15. HSQC spectrum of **2** in $CDCl_3$. 12

Figure S16. COSY spectrum of **2** in $CDCl_3$. 13

Figure S17. HMBC spectrum of **2** in $CDCl_3$. 13

Figure S18. NOESY spectrum of **2** in $CDCl_3$. 14

Figure S19. UV spectrum of **2**. 14

Figure S20. CD spectrum of **2**. 15

Figure S21. HRESIMS spectrum of **3**. 15

Figure S22. ^{1}H NMR spectrum of **3** in CD_3OD. 16

Figure S23. ^{13}C NMR spectrum of **3** in CD_3OD. 16

Figure S24. HSQC spectrum of **3** in CD_3OD. 17

Figure S25. COSY spectrum of **3** in CD_3OD. 17

Figure S26. HMBC spectrum of **3** in CD_3OD. 18

Figure S27. NOESY spectrum of **3** in CD_3OD. 18

Figure S28. UV spectrum of **3**. 19

Figure S29. CD spectrum of **3**. 19

Figure S30. HRESIMS spectrum of **4**. 20

Figure S31. ^{1}H NMR spectrum of **4** in CD_3OD. 20
Figure S32. ^{13}C NMR spectrum of **4** in CD_3OD. 21
Figure S33. HSQC spectrum of **4** in CD_3OD. 21
Figure S34. COSY spectrum of **4** in CD_3OD. 22
Figure S35. HMBC spectrum of **4** in CD_3OD. 22
Figure S36. NOESY spectrum of **4** in CD_3OD. 23
Figure S37. UV spectrum of **4**. 23
Figure S38. CD spectrum of **4**. 24
Figure S39. HRESIMS spectrum of **5**. 24
Figure S40. ^{1}H NMR spectrum of **5** in CD_3OD. 25
Figure S41. ^{13}C NMR spectrum of **5** in CD_3OD. 25
Figure S42. HSQC spectrum of **5** in CD_3OD. 26
Figure S43. COSY spectrum of **5** in CD_3OD. 26
Figure S44. HMBC spectrum of **5** in CD_3OD. 27
Figure S45. NOESY spectrum of **5** in CD_3OD. 27
Figure S46. HRESIMS spectrum of **6**. 28
Figure S47. ^{1}H NMR spectrum of **6** in CD_3OD. 28
Figure S48. ^{13}C NMR spectrum of **6** in CD_3OD. 29
Figure S49. HSQC spectrum of **6** in CD_3OD. 29
Figure S50. COSY spectrum of **6** in CD_3OD. 30
Figure S51. HMBC spectrum of **6** in CD_3OD. 30
Figure S52. NOESY spectrum of **6** in CD_3OD. 31
Figure S53. UV spectrum of **6**. 31
Figure S54. CD spectrum of **6**. 32
Figure S55. HRESIMS spectrum of **7**. 32
Figure S56. ^{1}H NMR spectrum of **7** in CD_3OD. 33
Figure S57. ^{13}C NMR spectrum of **7** in CD_3OD. 33
Figure S58. HSQC spectrum of **7** in CD_3OD. 34
Figure S59. COSY spectrum of **7** in CD_3OD. 34
Figure S60. HMBC spectrum of **7** in CD_3OD. 35
Figure S61. NOESY spectrum of **7** in CD_3OD. 35
Figure S62. UV spectrum of **7**. 36

Figure S63. CD spectrum of **7**. 36

Figure S64. HRESIMS spectrum of **8**. 37

Figure S65. ^{1}H NMR spectrum of **8** in $CDCl_3$. 37

Figure S66. ^{13}C NMR spectrum of **8** in $CDCl_3$. 38

Figure S67. HSQC spectrum of **8** in $CDCl_3$. 38

Figure S68. COSY spectrum of **8** in $CDCl_3$. 39

Figure S69. HMBC spectrum of **8** in $CDCl_3$. 39

Figure S70. NOESY spectrum of **8** in $CDCl_3$. 40

Figure S71. HRESIMS spectrum of **9** **(***m*/*z* $[M + Na]^+$**)**. 40

Figure S72. ^{1}H NMR spectrum of **9** in DMSO-d_6. 41

Figure S73. ^{13}C NMR spectrum of **9** in DMSO- d_6. 41

Figure S74. ^{1}H NMR spectrum of (*R*)-MTPA ester of **7**. 42

Figure S75. HSQC spectrum of (*R*)-MTPA ester of **7**. 42

Figure S76. ^{1}H NMR spectrum of (*S*)-MTPA ester of **7**. 43

Figure S77. HSQC spectrum of (*S*)-MTPA ester of **7**. 43

Figure S78. Comparison of the experimental ^{13}C NMR data of compound **1** and the calculated chemical shifts of (3*S*, 4*R*, 7S, 8*R*, 9*R*, 16*S*, 18*R*, 21*R*-**1**, and 3*S*, 4*R*, 7S, 8*R*, 9*R*, 16*S*, 18*R*, 21*S*-**1**). 44

Figure S79. DP4+ analysis of compound **1**. 45

Figure S80. ^{13}C NMR spectrum of **11** in CD_3OD. 46

Table S1. Gibbs free energy and Boltzmann population of low energy of 3*S*, 4*R*, 7S, 8*R*, 9*R*, 16*S*, 18*R*, 21*R* -**1** in MeOH. 46

Table S2. Gibbs free energy and Boltzmann population of low energy of 3*S*, 4*R*, 8*R*, 9*R*, 16*S*, 18*R*, 21*R* -**3** in MeOH. 46

Table S3. Gibbs free energy and Boltzmann population of low energy of 3*S*, 4*R*, 8*R*, 9*R*, 16*S*, 18*R*, 21*R* -**4** in MeOH. 47

Table S4. Gibbs free energy and Boltzmann population of low energy of 9*S*, 10*S*, 12*S*-**6** in MeOH. 47

Table S5. Gibbs free energy and Boltzmann population of low energy of 2*S*, 4*S*, 8*S* -**7** in MeOH. 47

Table S6. Gibbs free energy and Boltzmann population of low energy of 2*R*, 4*R*, 8*S* -**7** in MeOH. 48

Table S7. Gibbs free energy and Boltzmann population of low energy of 2*S*, 4*R*, 8*S* -**7** in MeOH. 48

Table S8. Gibbs free energy and Boltzmann population of low energy of 2*R*, 4*S*, 8*S* **-7** in MeOH. 49

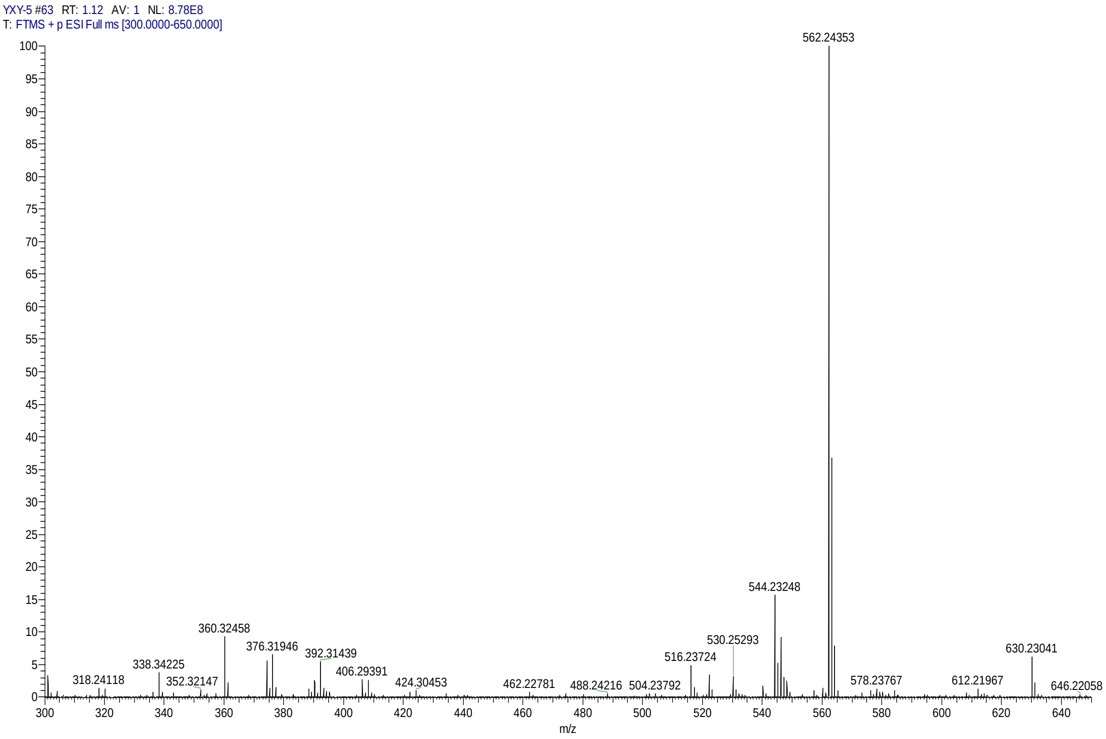

Figure S1. HRESIMS spectrum of **1** (*m*/*z* [M + Na]$^{+}$).

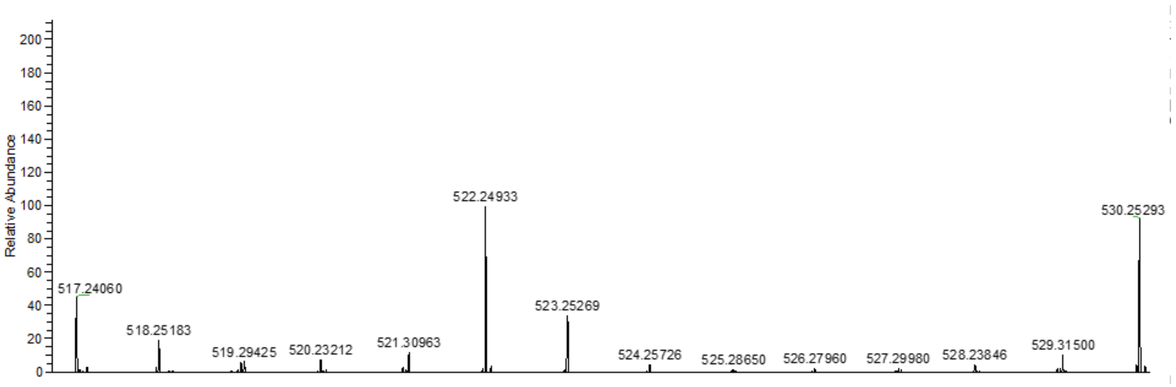

Figure S2. HRESIMS spectrum of **1** (*m*/*z* [M + H - H_2O]$^{+}$).

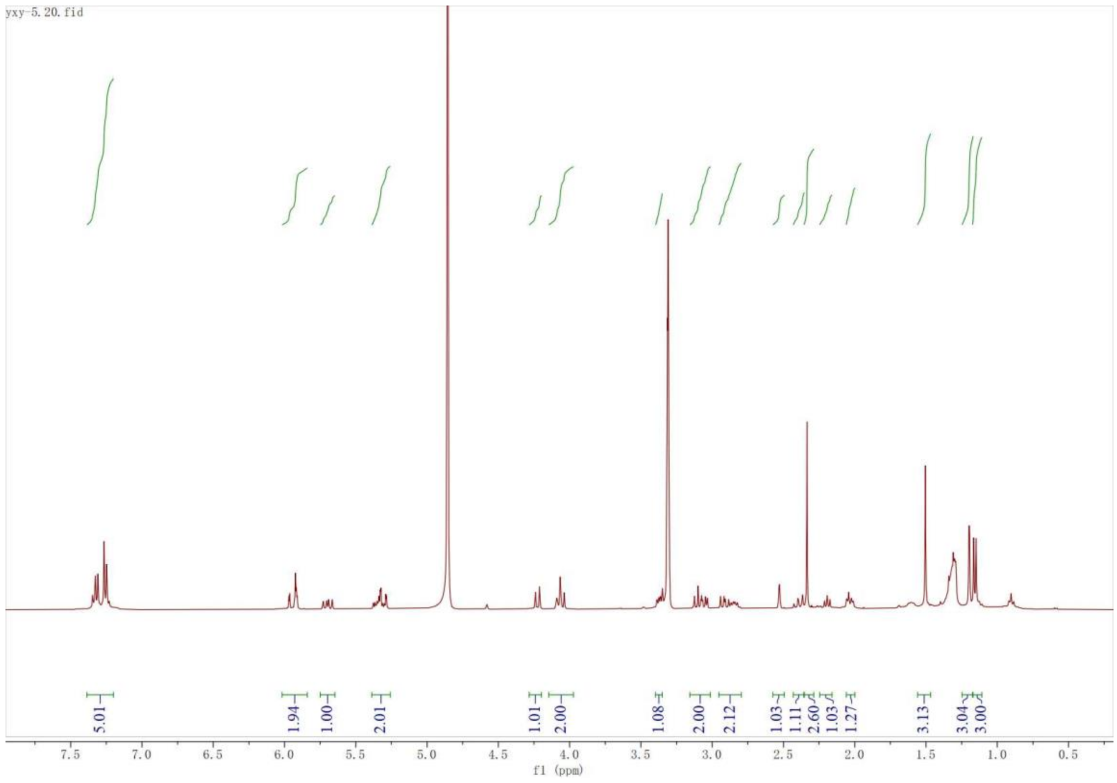

Figure S3. ^{1}H NMR spectrum of **1** in CD_3OD.

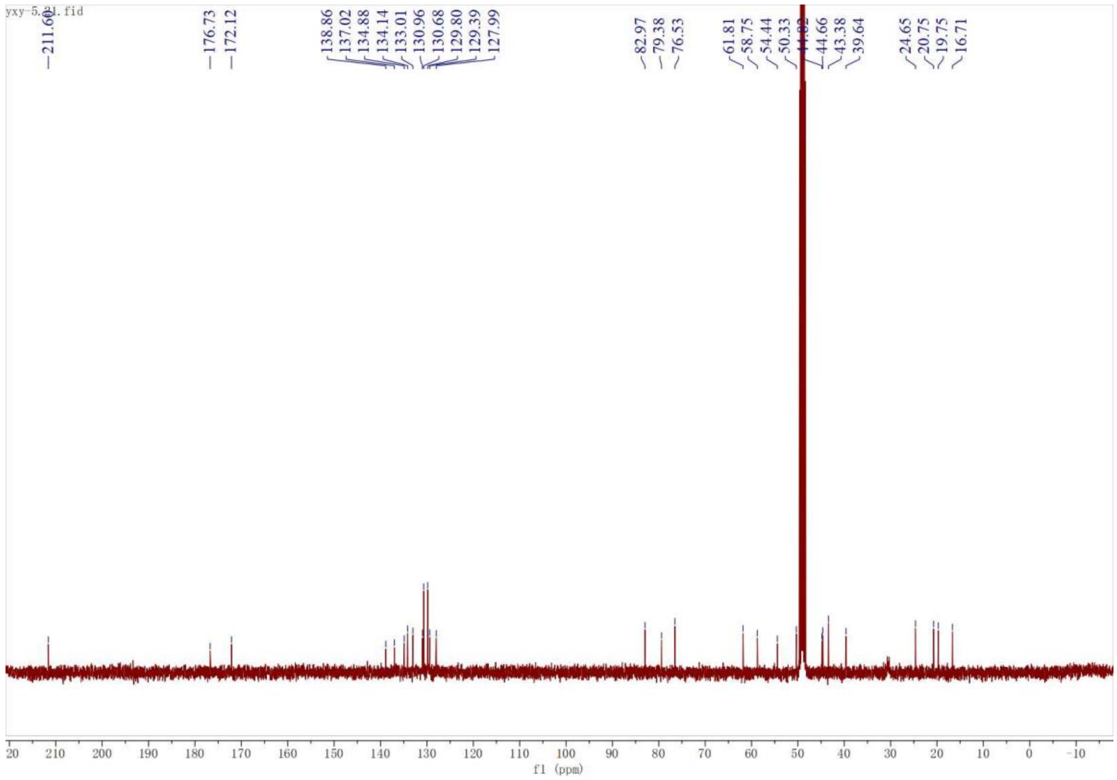

Figure S4. ^{13}C NMR spectrum of **1** in CD_3OD.

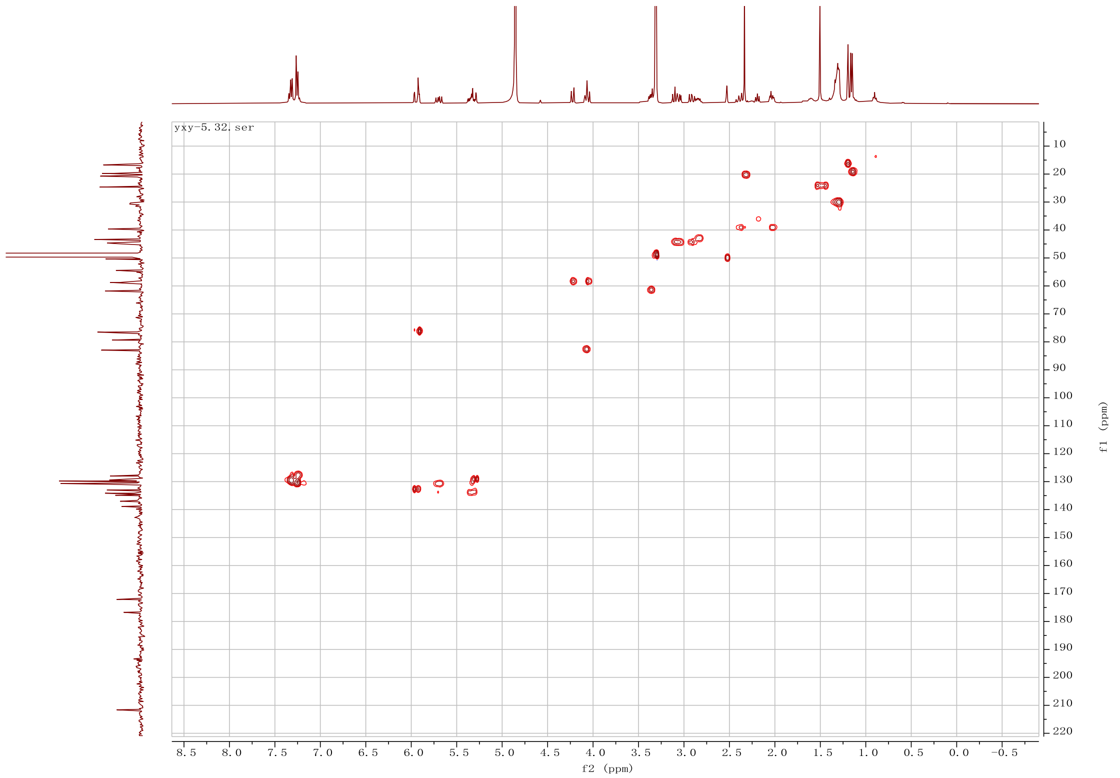

Figure S5. HSQC spectrum of **1** in CD_3OD.

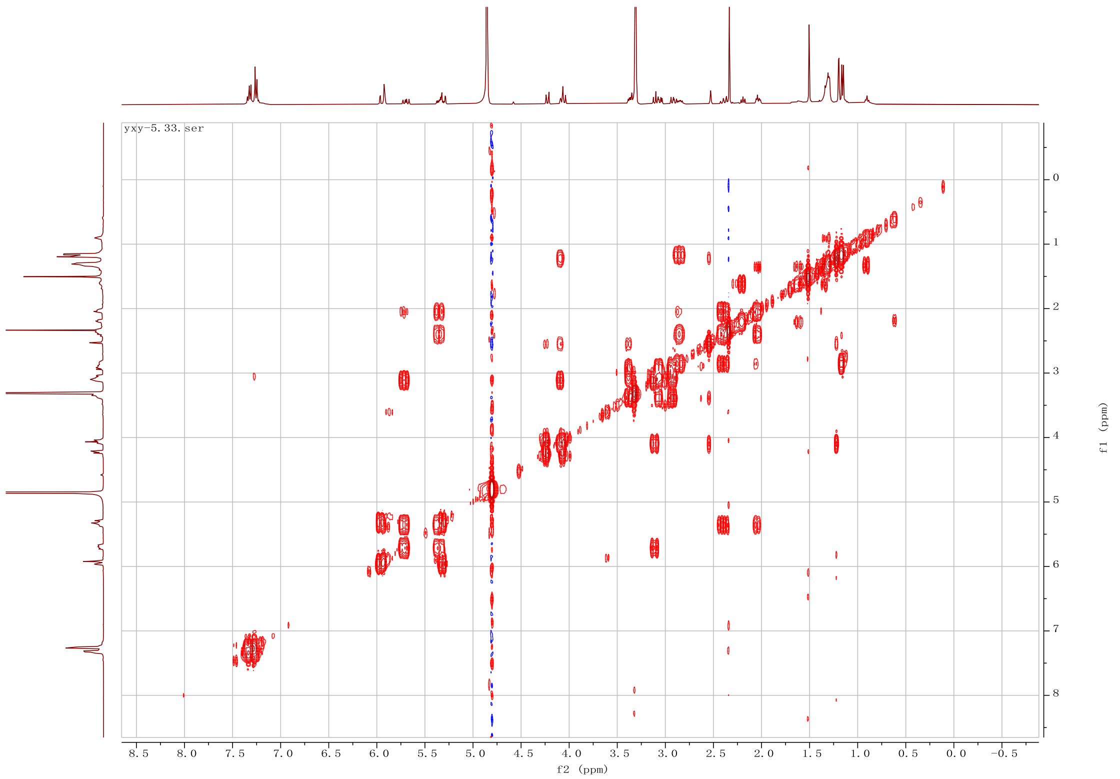

Figure S6. COSY spectrum of **1** in CD_3OD.

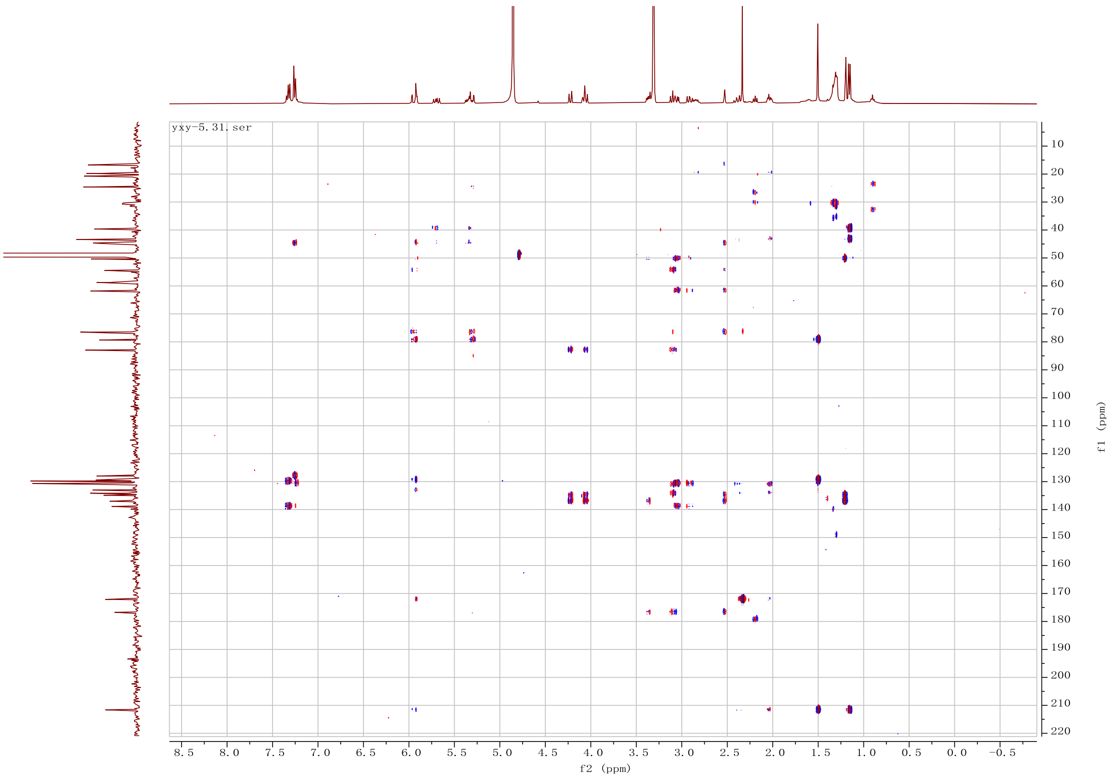

Figure S7. HMBC spectrum of **1** in CD_3OD.

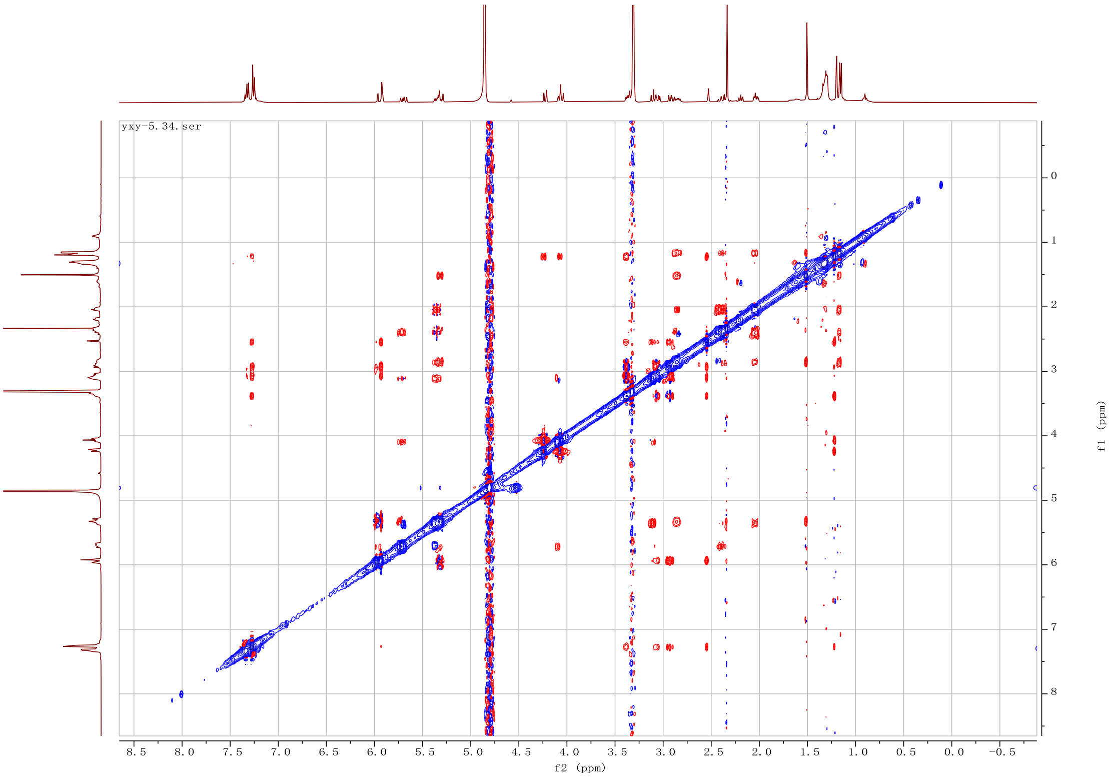

Figure S8. NOESY spectrum of **1** in CD_3OD.

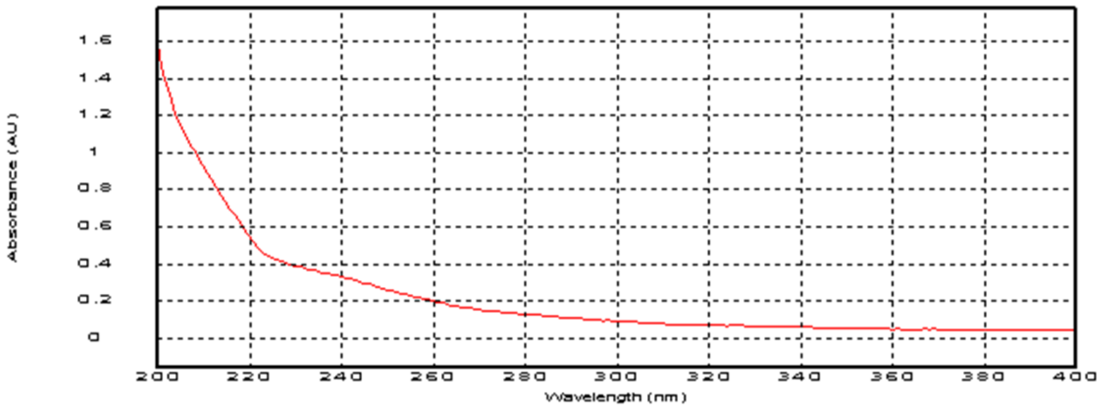

Figure S9. UV spectrum of **1**.

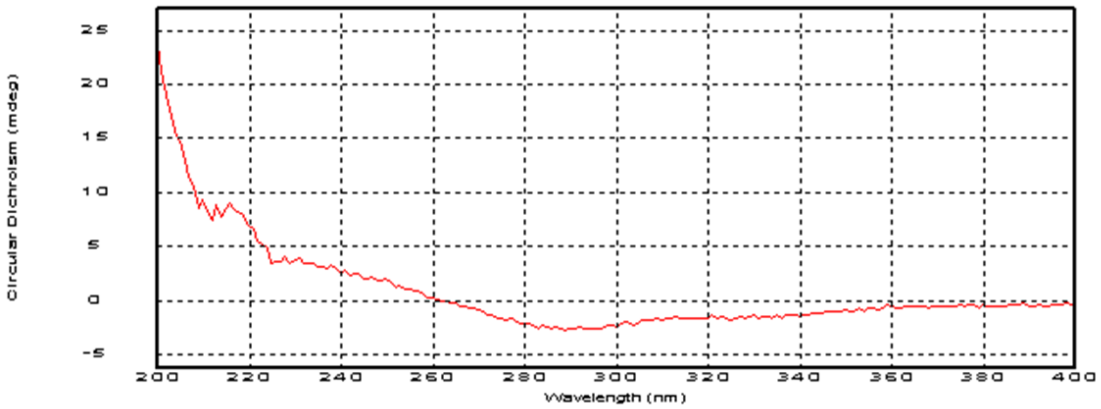

Figure S10. CD spectrum of **1**.

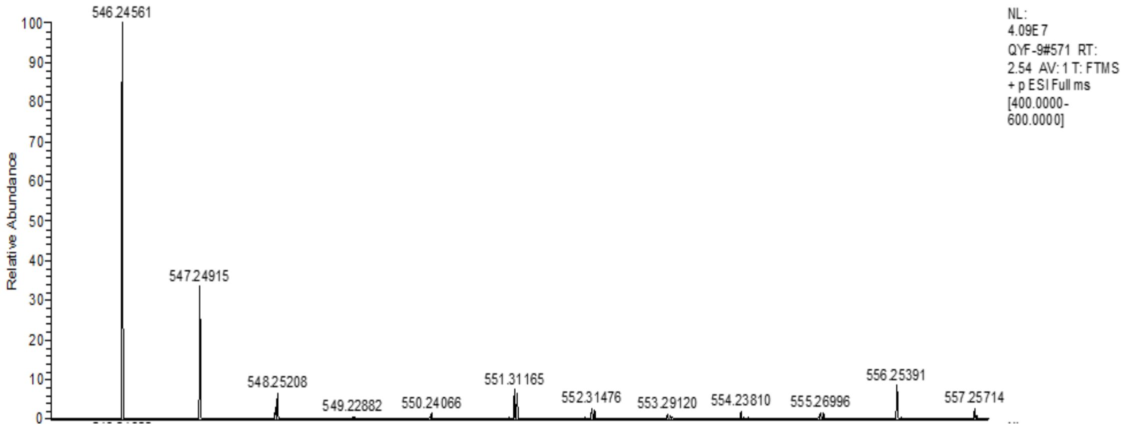

Figure S11. HRESIMS spectrum of **2** (*m*/*z* [M + Na]$^+$).

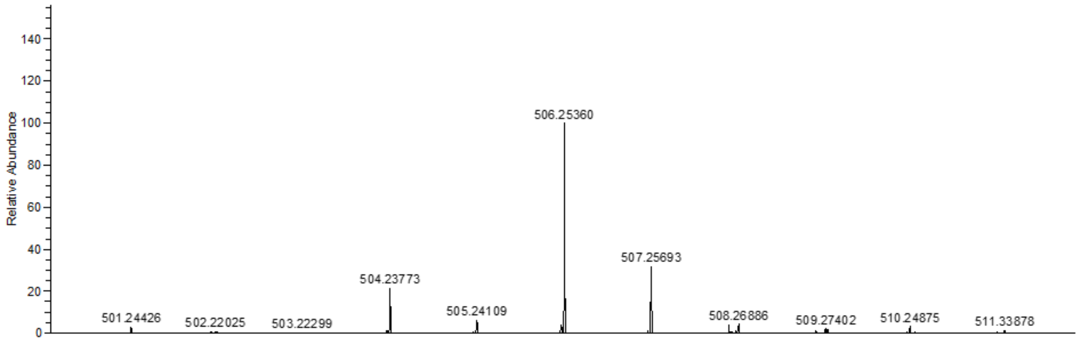

Figure S12. HRESIMS spectrum of **2** (*m*/*z* [M + H - H_2O]$^+$).

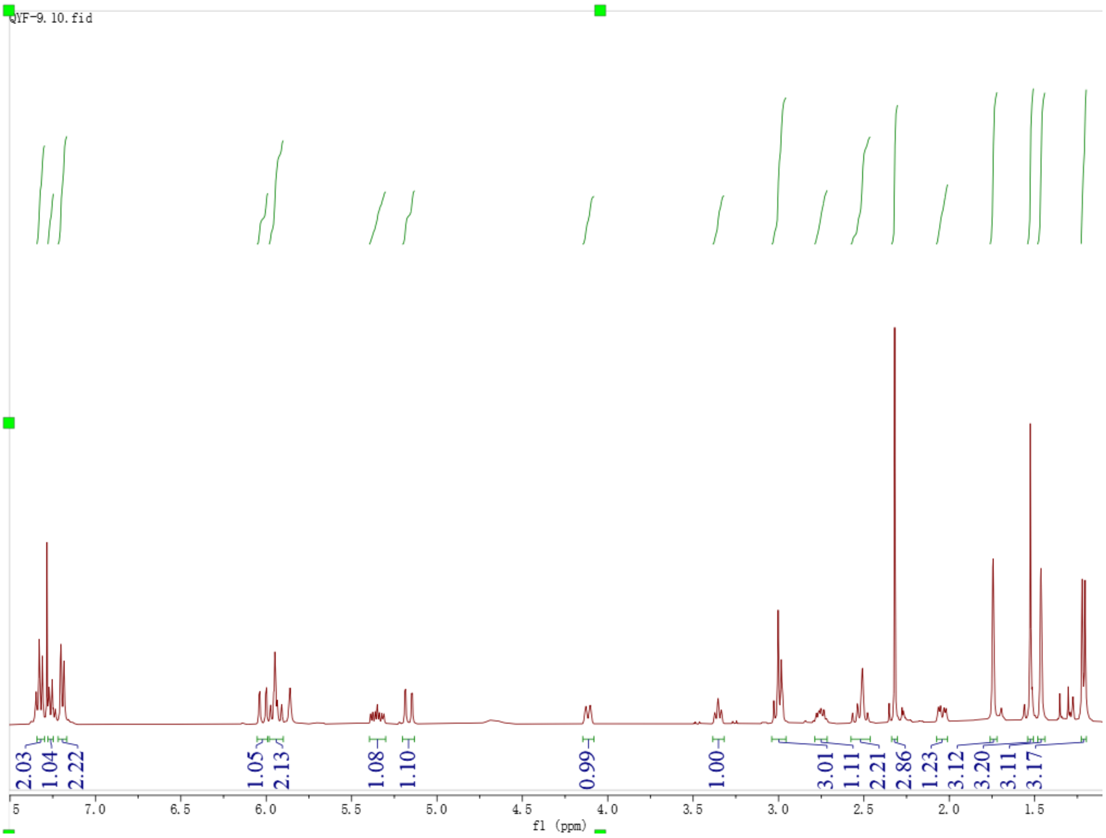

Figure S13. ^{1}H NMR spectrum of **2** in $CDCl_3$.

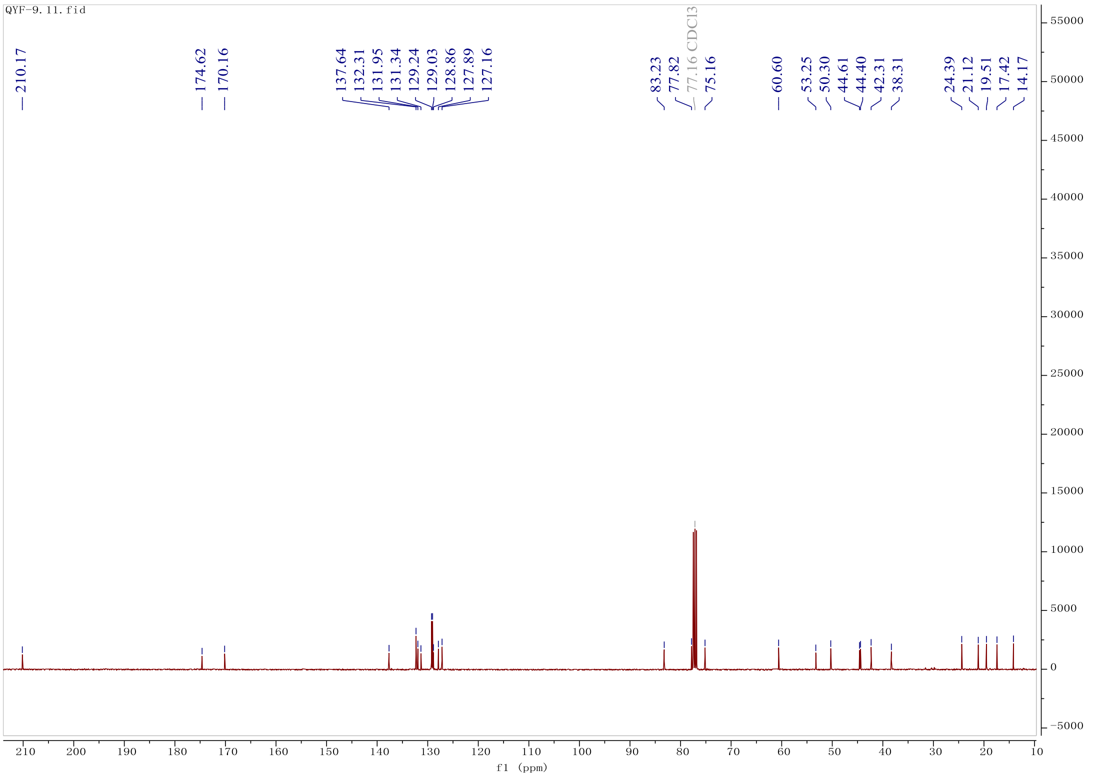

Figure S14. ^{13}C NMR spectrum of **2** in $CDCl_3$.

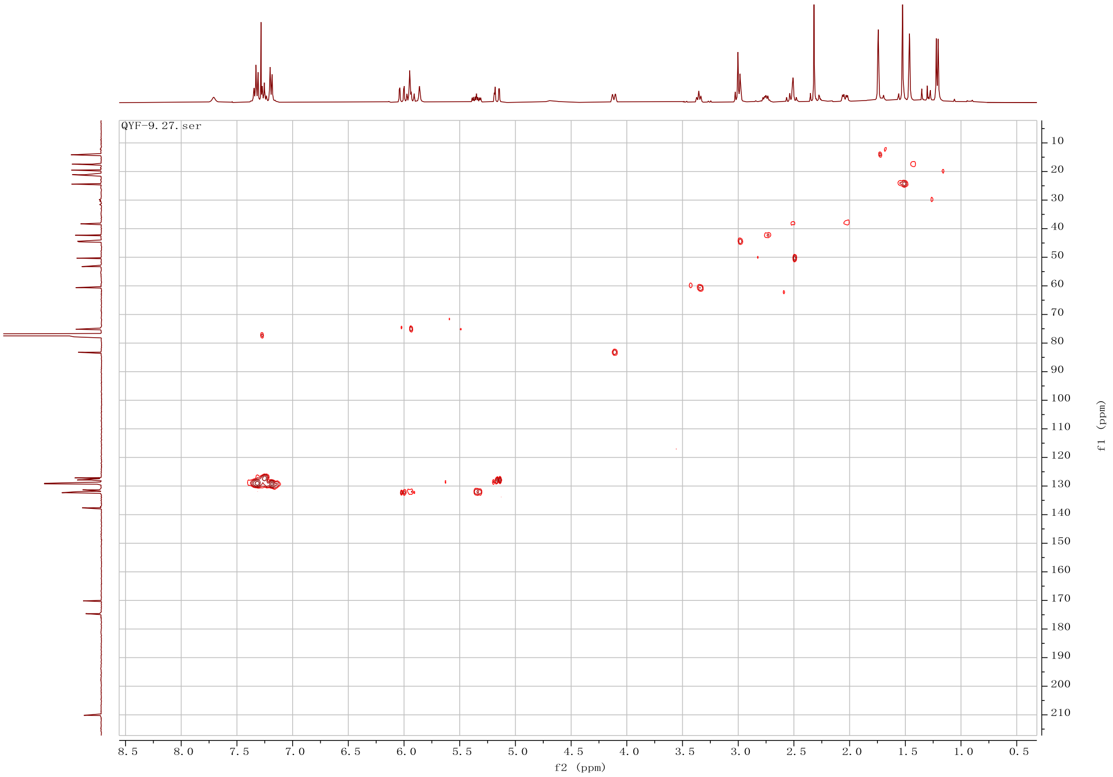

Figure S15. HSQC spectrum of **2** in $CDCl_3$.

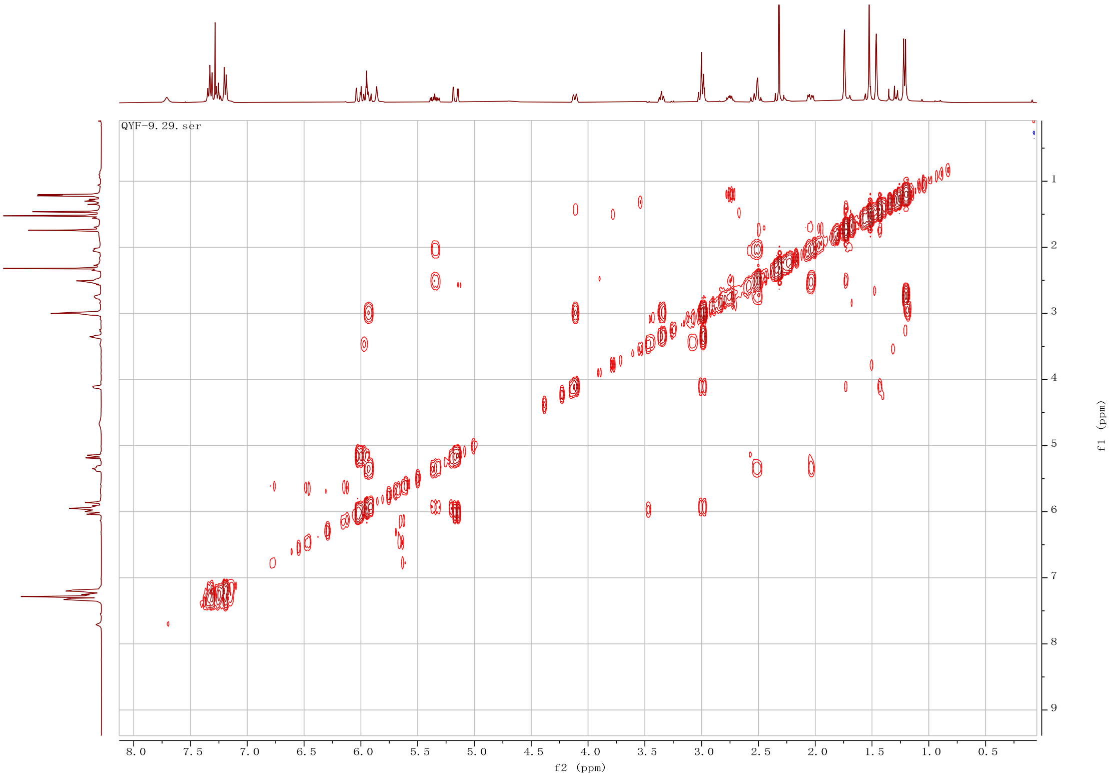

Figure S16. COSY spectrum of **2** in $CDCl_3$.

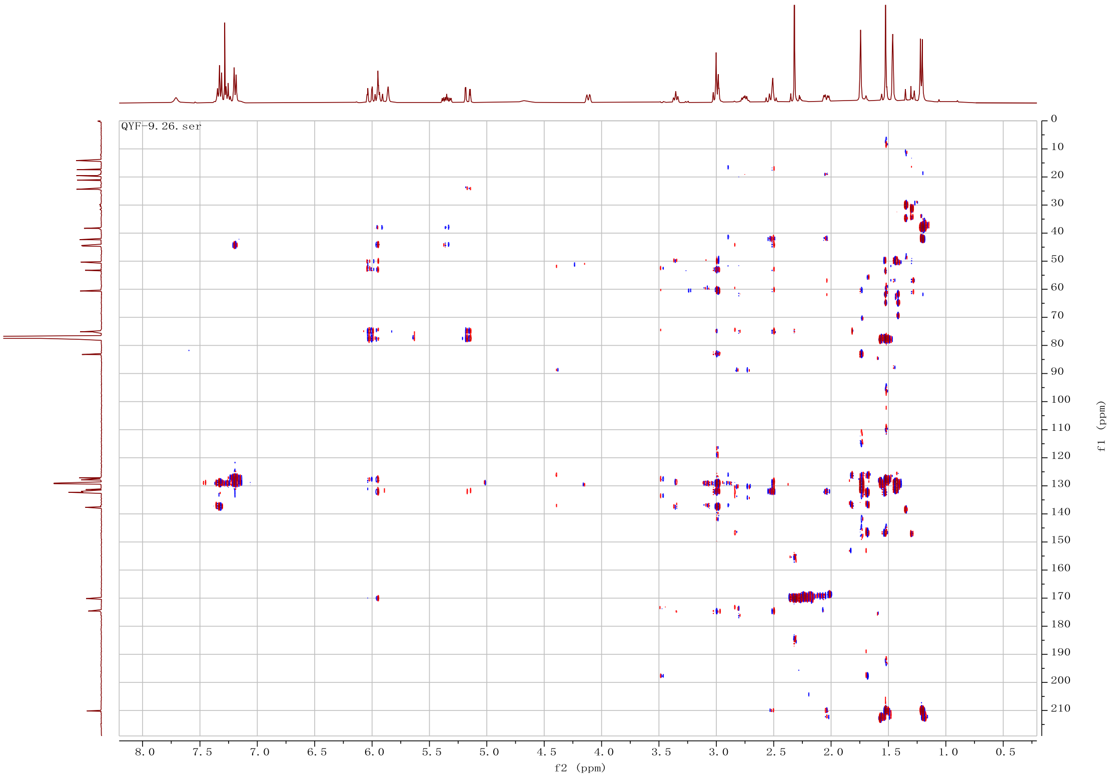

Figure S17. HMBC spectrum of **2** in $CDCl_3$.

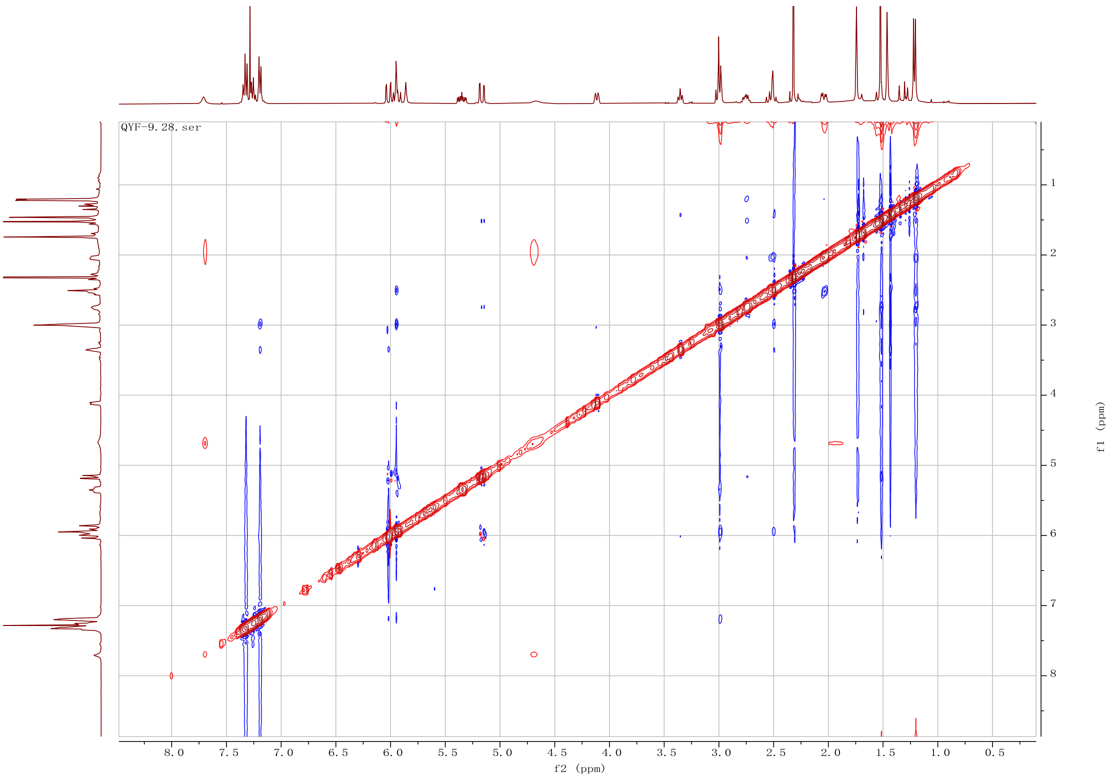

Figure S18. NOESY spectrum of **2** in $CDCl_3$.

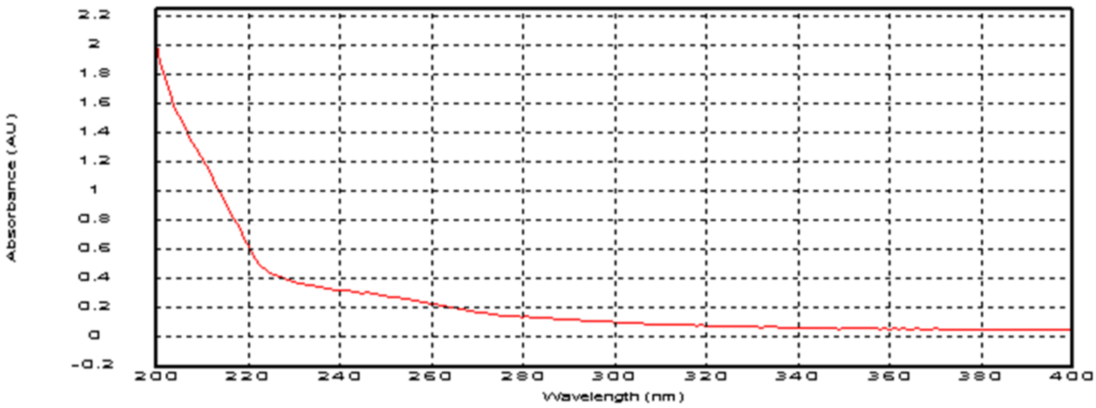

Figure S19. UV spectrum of **2**.

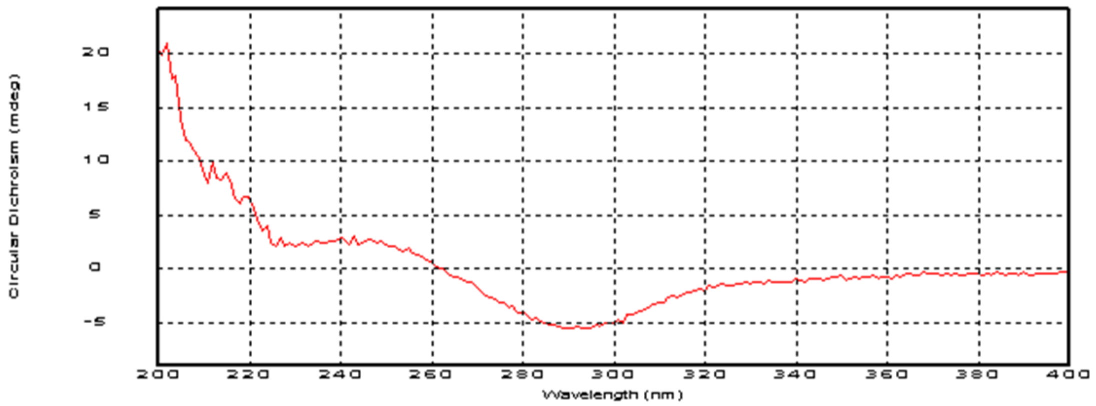

Figure S20. CD spectrum of **2**.

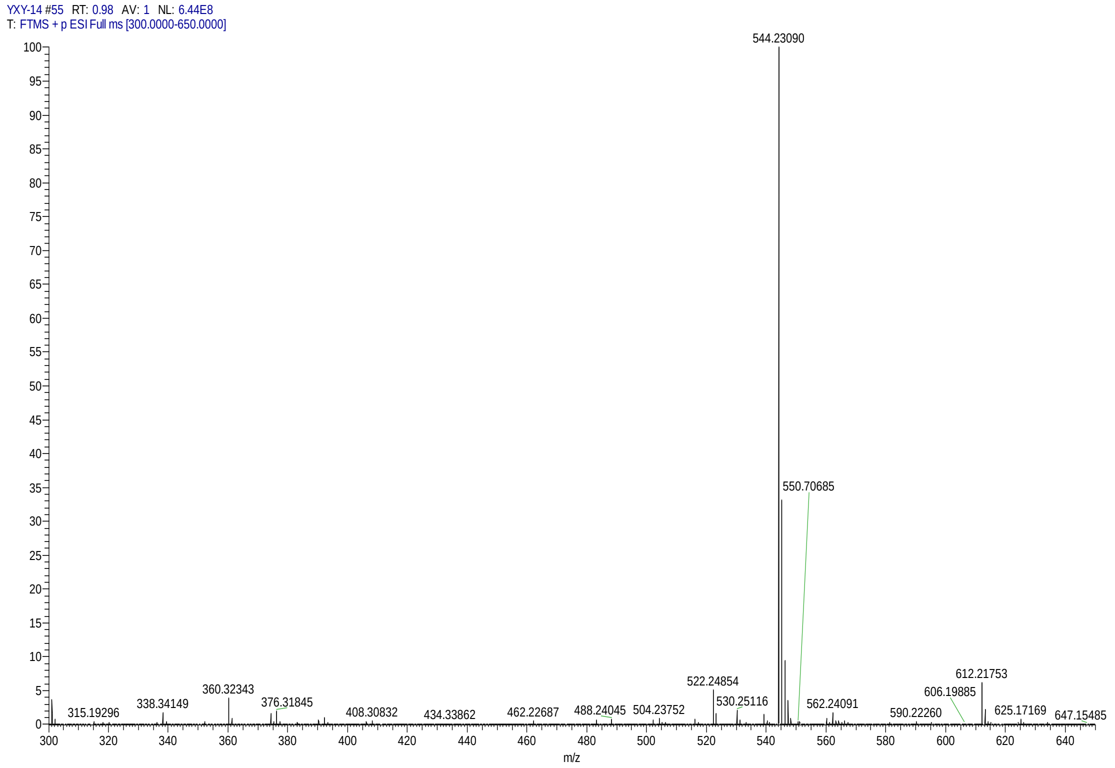

Figure S21. HRESIMS spectrum of **3**.

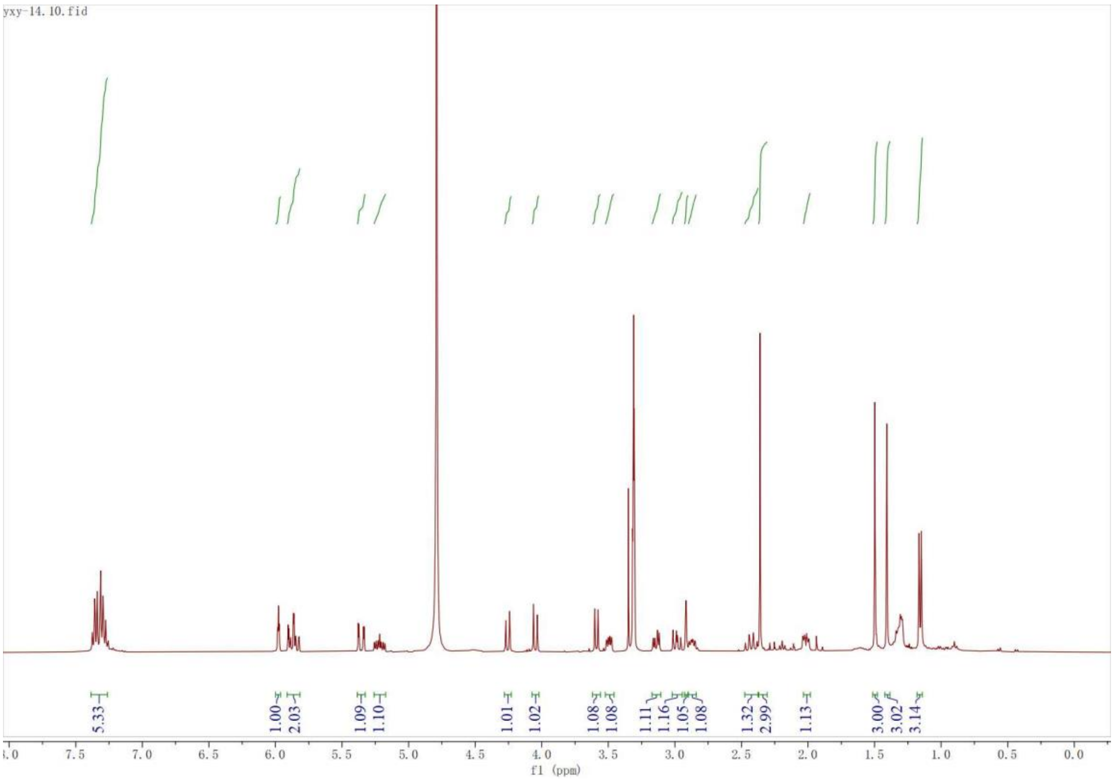

Figure S22. ^{1}H NMR spectrum of **3** in CD_3OD.

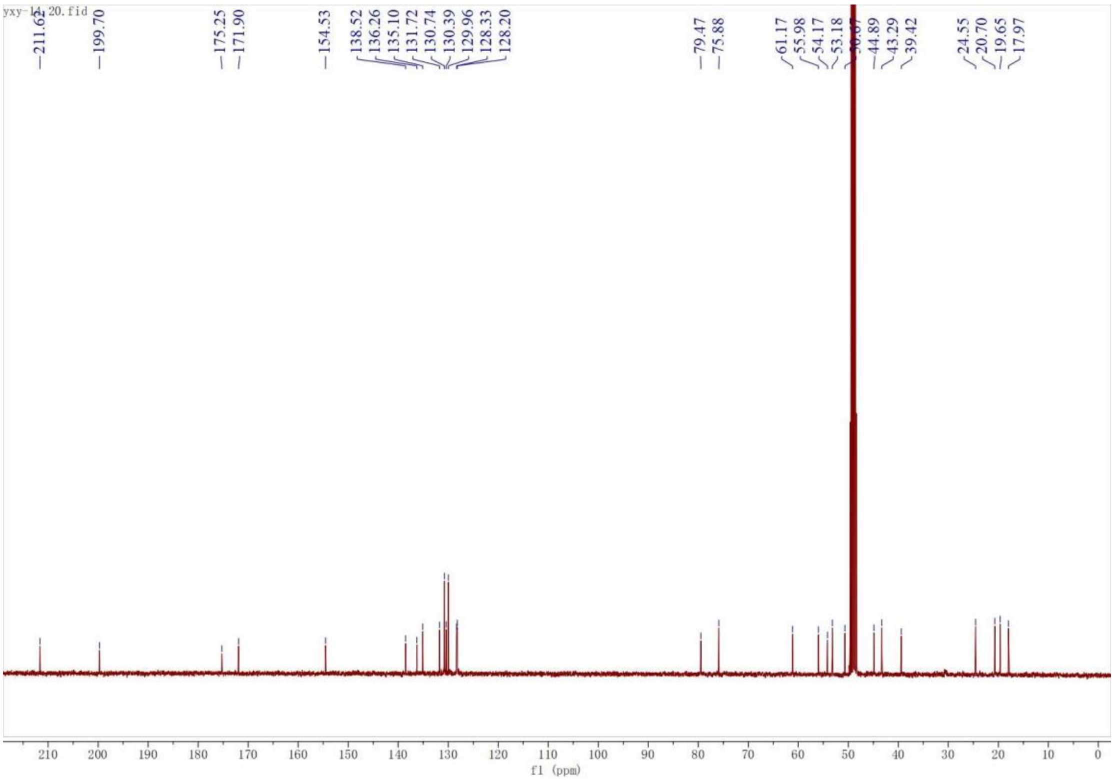

Figure S23. ^{13}C NMR spectrum of **3** in CD_3OD.

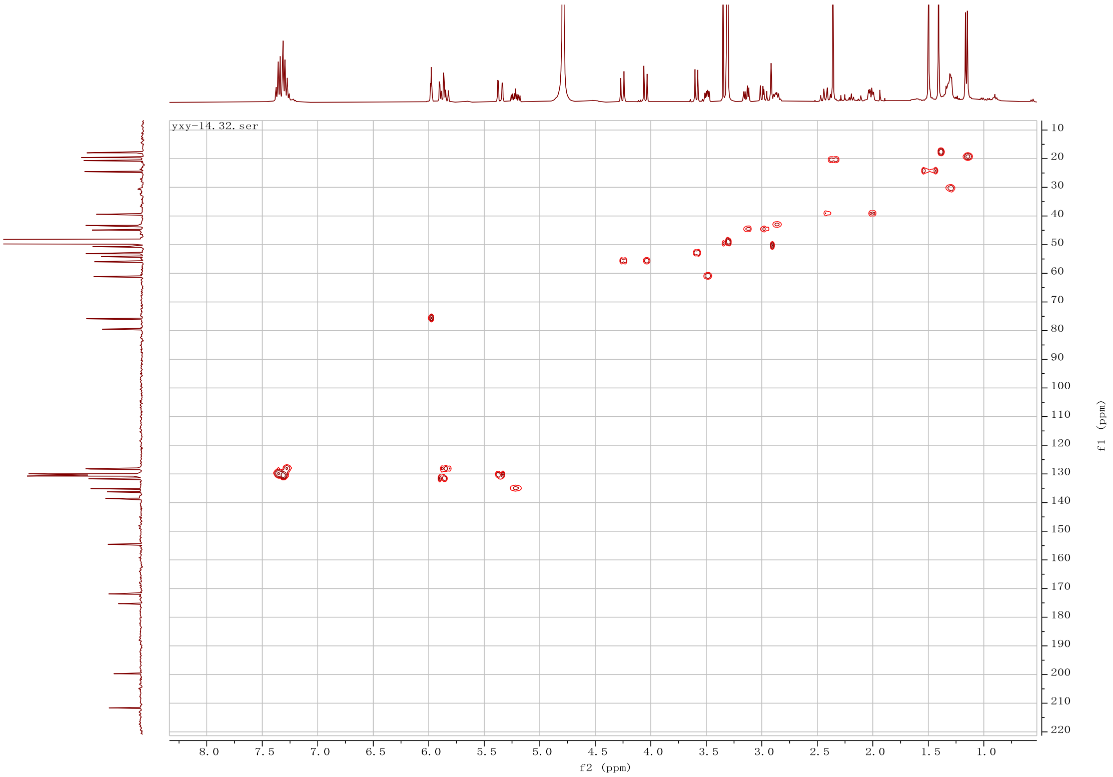

Figure S24. HSQC spectrum of **3** in CD_3OD.

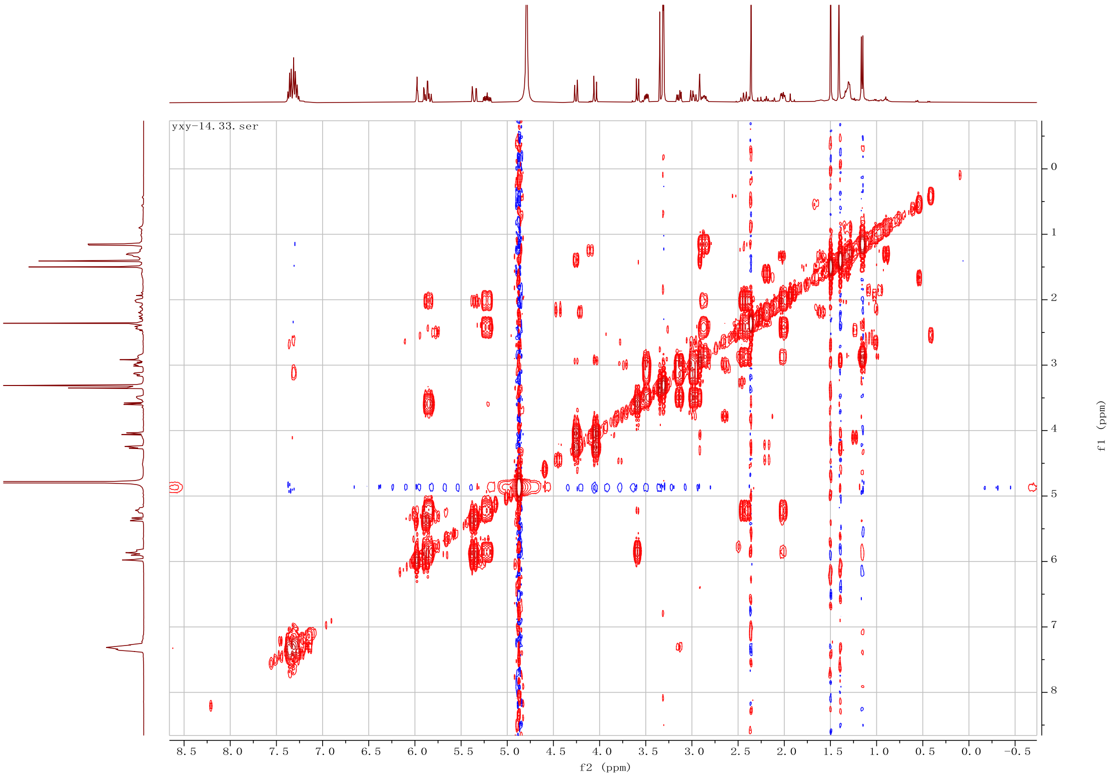

Figure S25. COSY spectrum of **3** in CD_3OD.

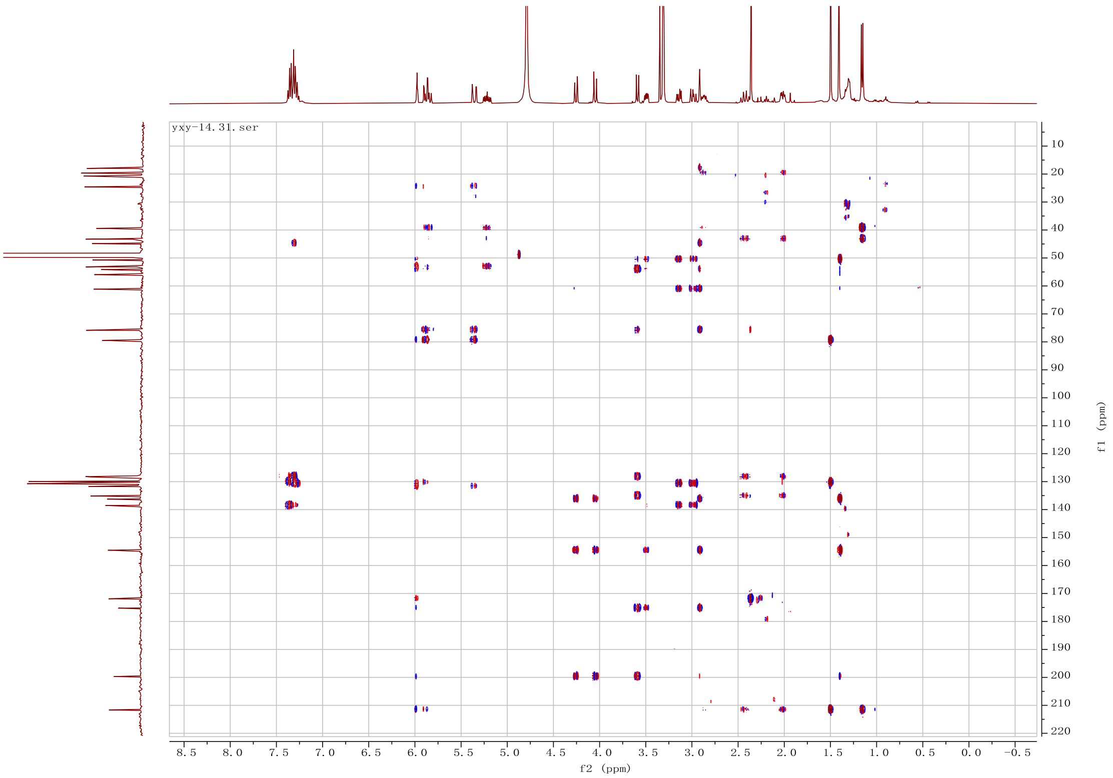

Figure S26. HMBC spectrum of **3** in CD_3OD.

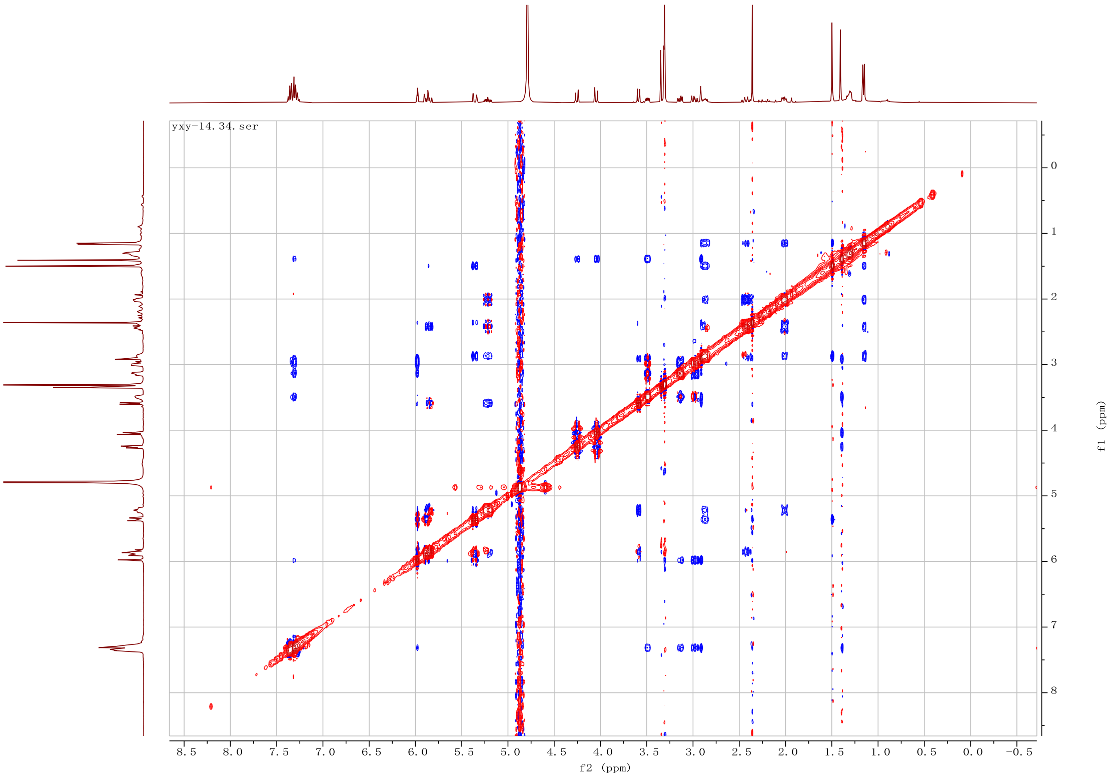

Figure S27. NOESY spectrum of **3** in CD_3OD.

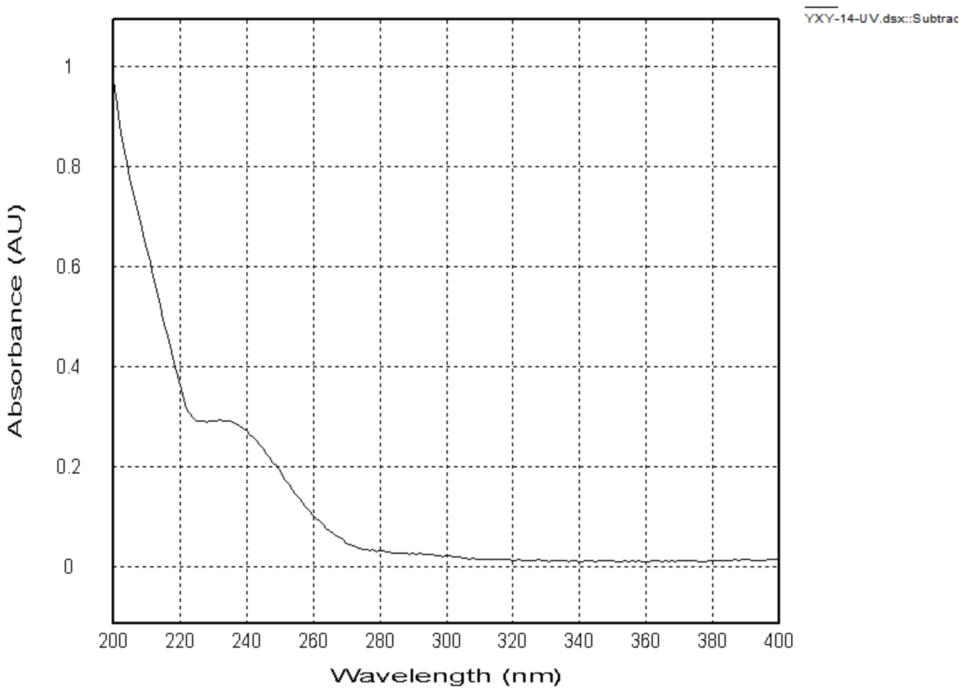

Figure S28. UV spectrum of **3**.

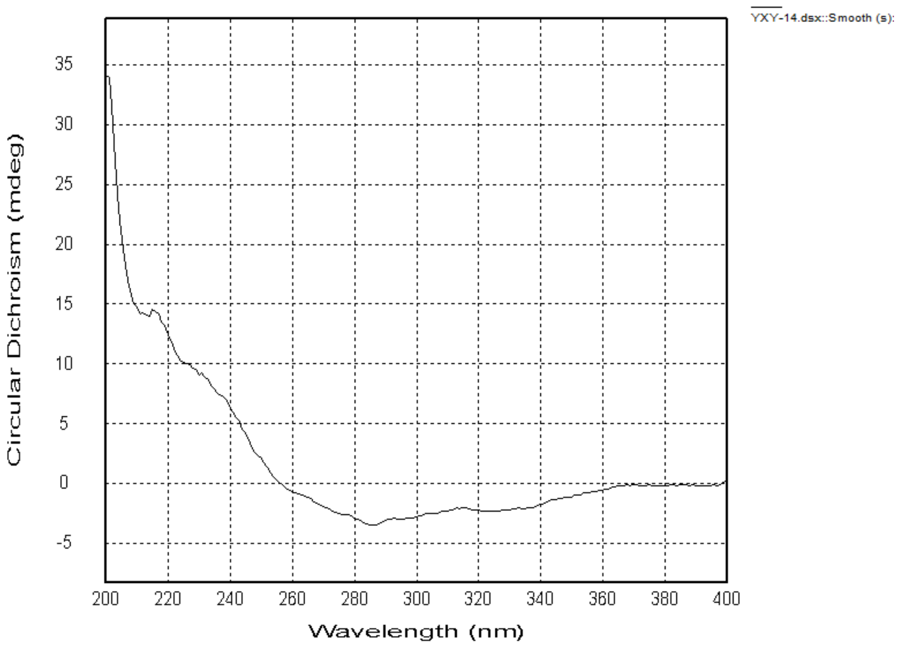

Figure S29. CD spectrum of **3**.

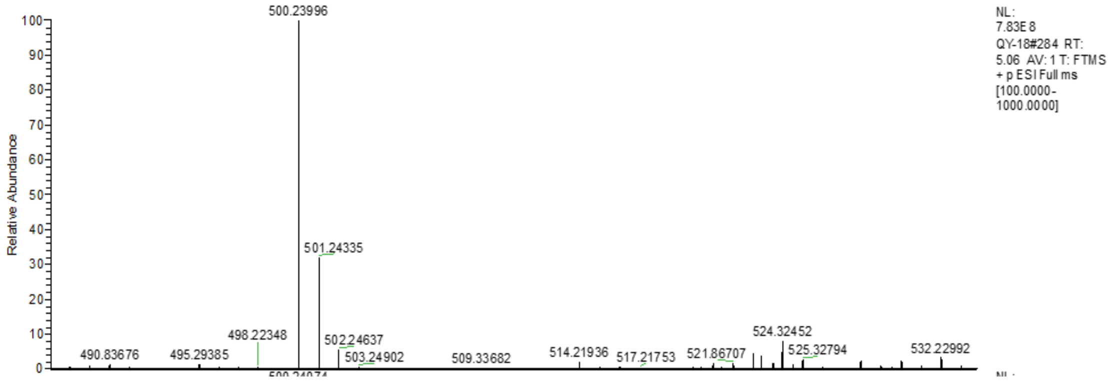

Figure S30. HRESIMS spectrum of **4**.

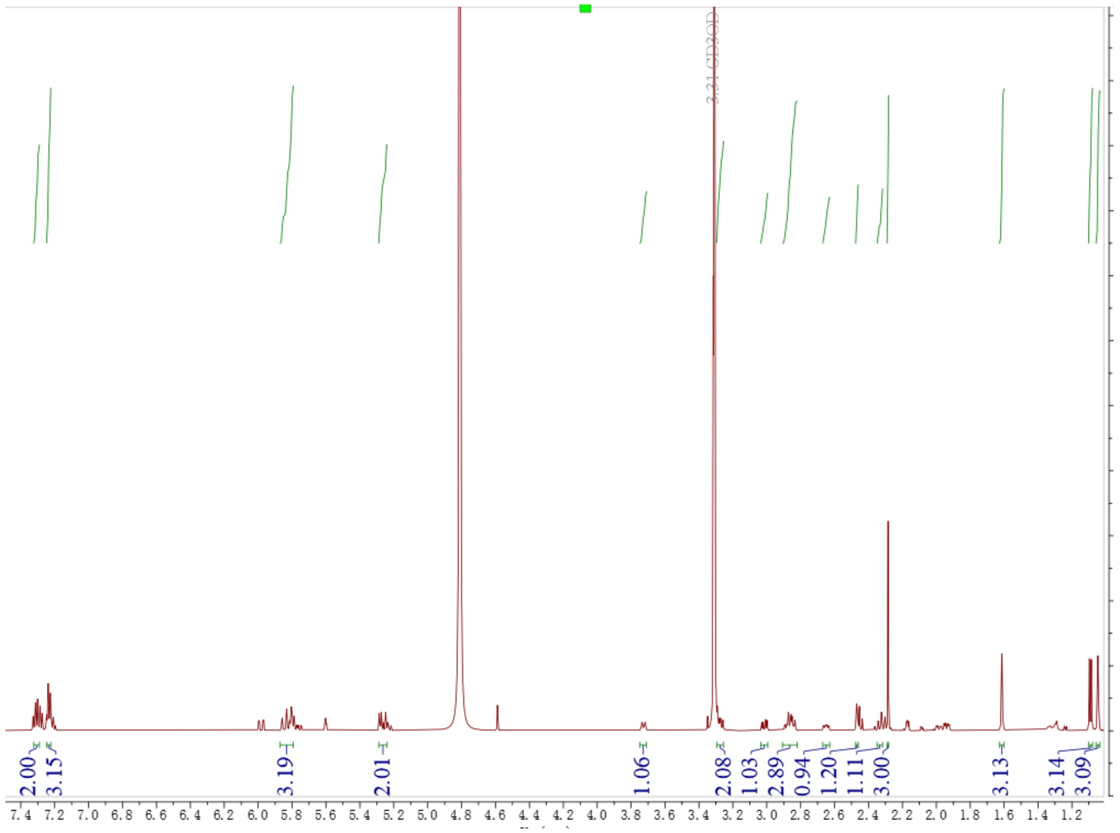

Figure S31. ^{1}H NMR spectrum of **4** in CD_3OD.

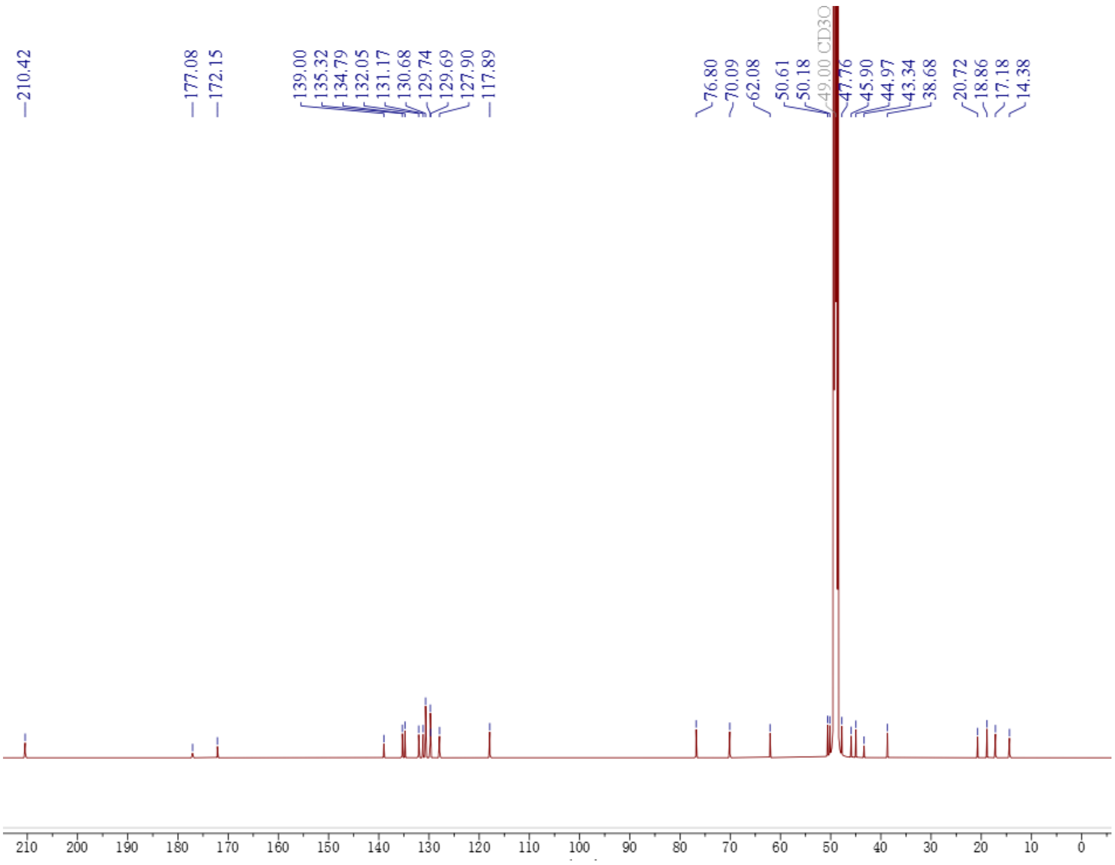

Figure S32. ^{13}C NMR spectrum of **4** in CD_3OD.

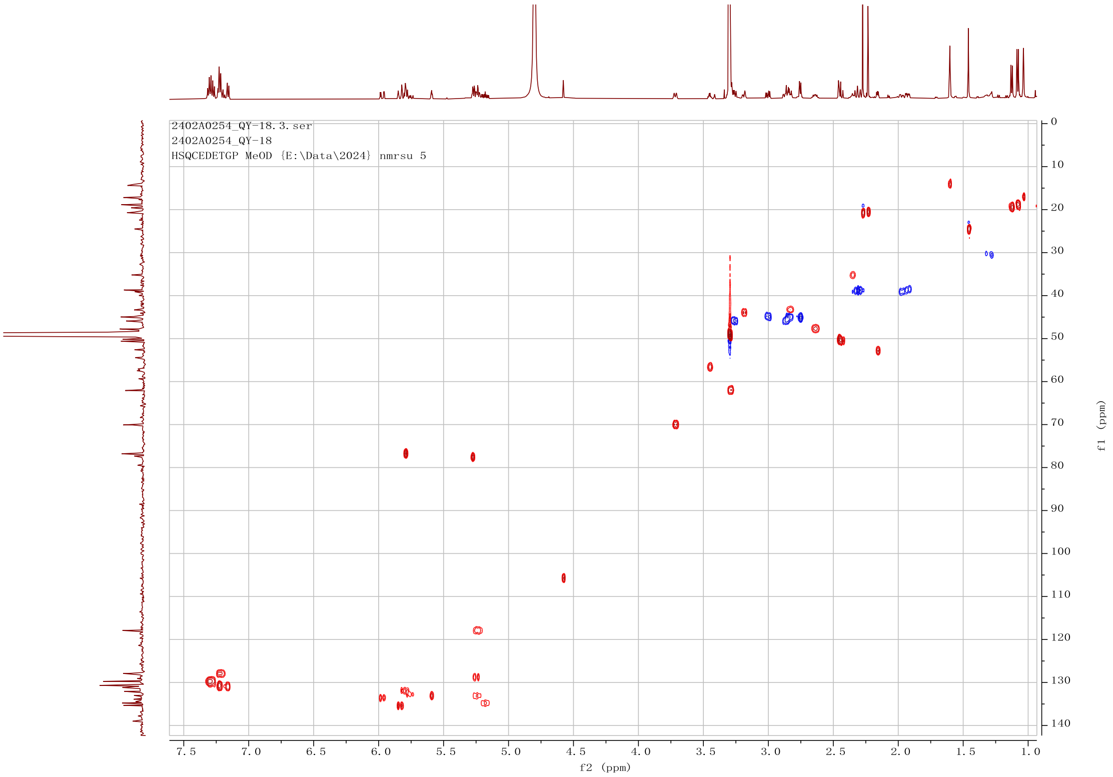

Figure S33. HSQC spectrum of **4** in CD_3OD.

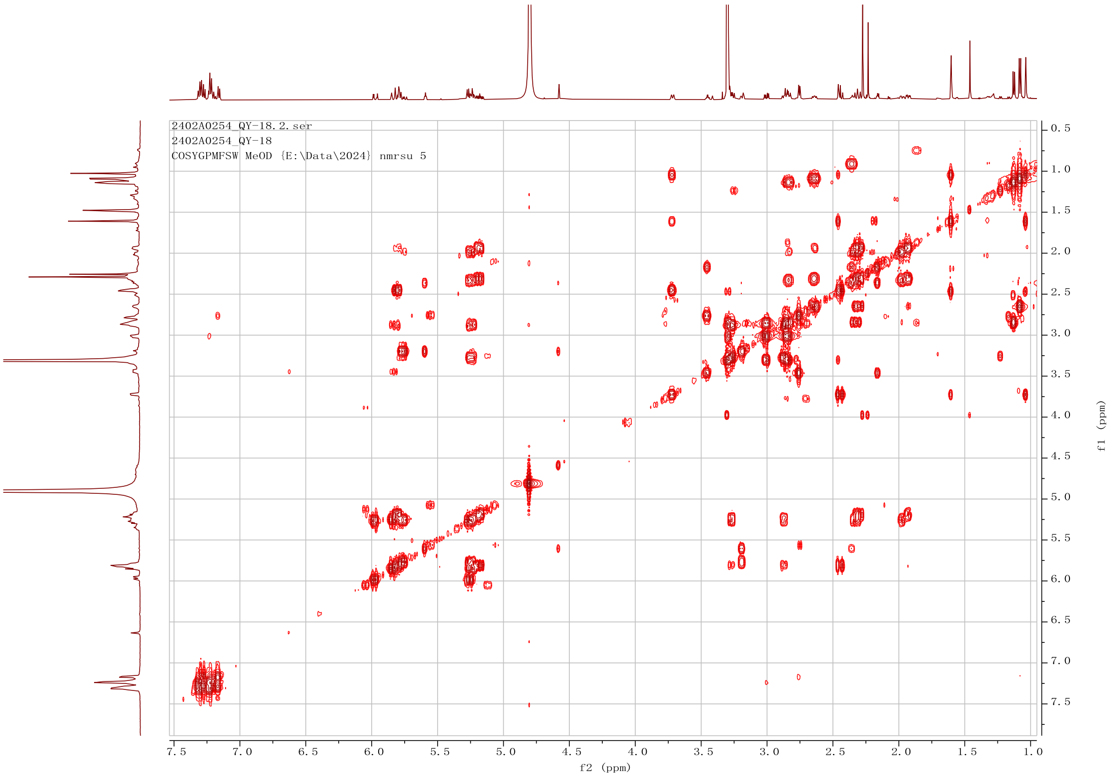

Figure S34. COSY spectrum of **4** in CD_3OD.

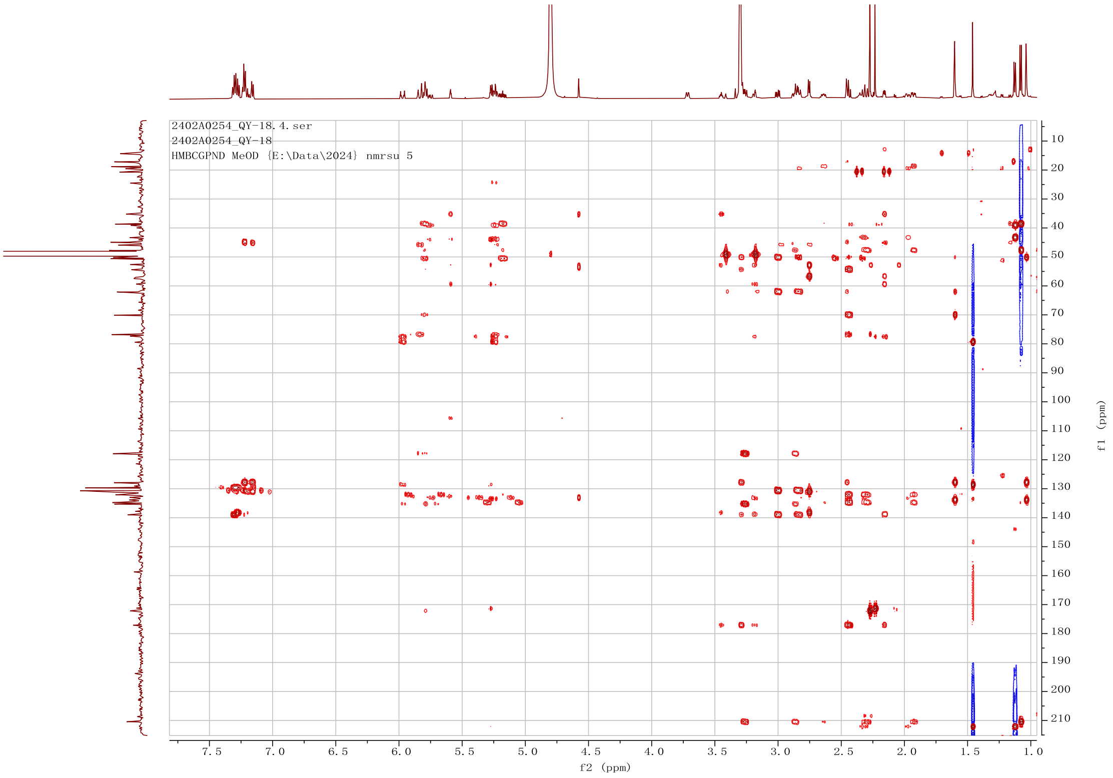

Figure S35. HMBC spectrum of **4** in CD_3OD.

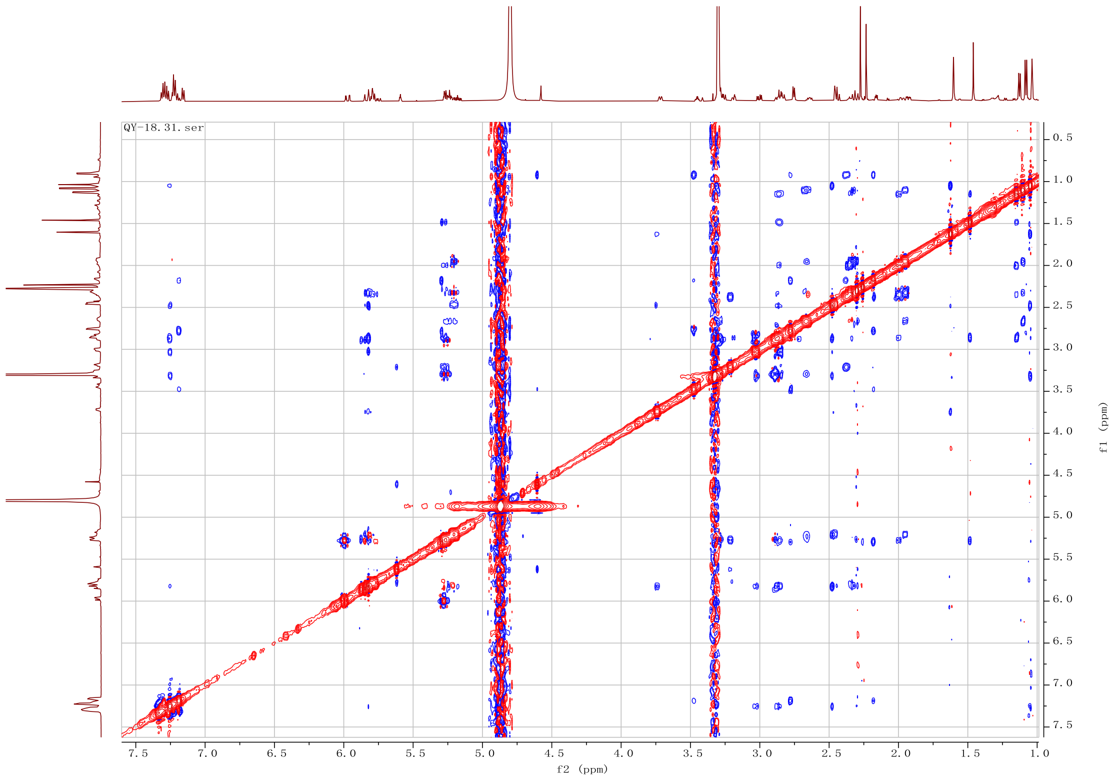

Figure S36. NOESY spectrum of **4** in CD_3OD.

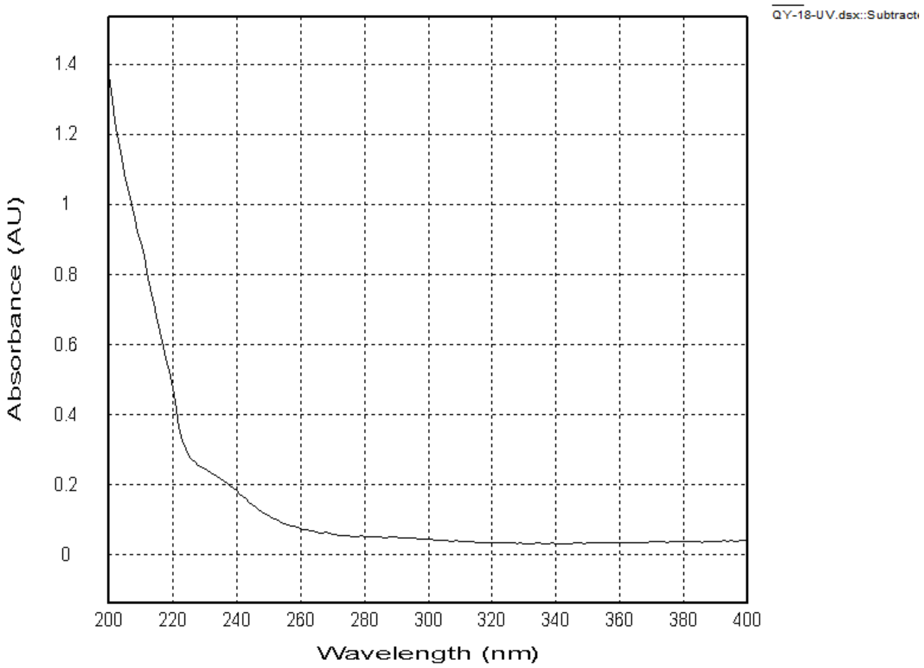

Figure S37. UV spectrum of **4**.

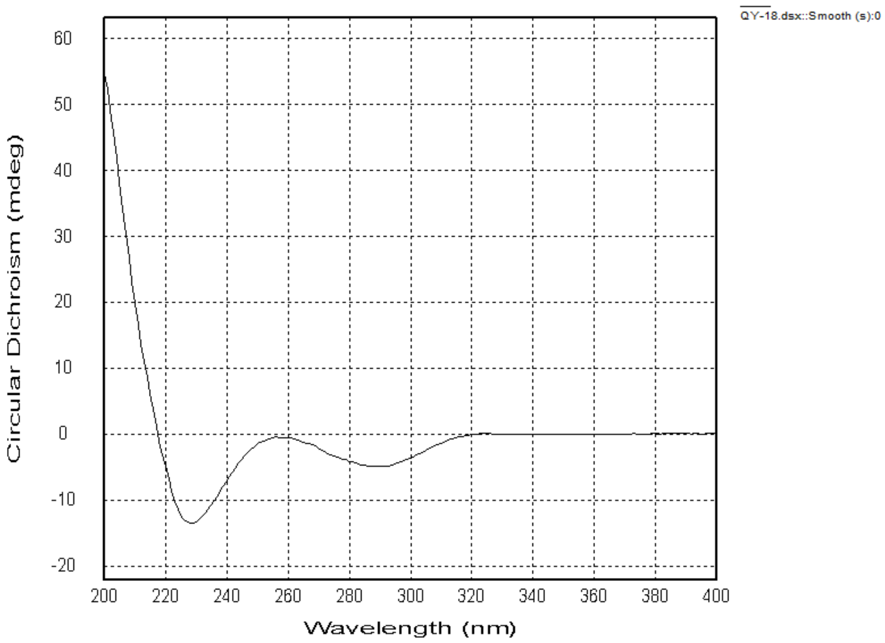

Figure S38. CD spectrum of **4**.

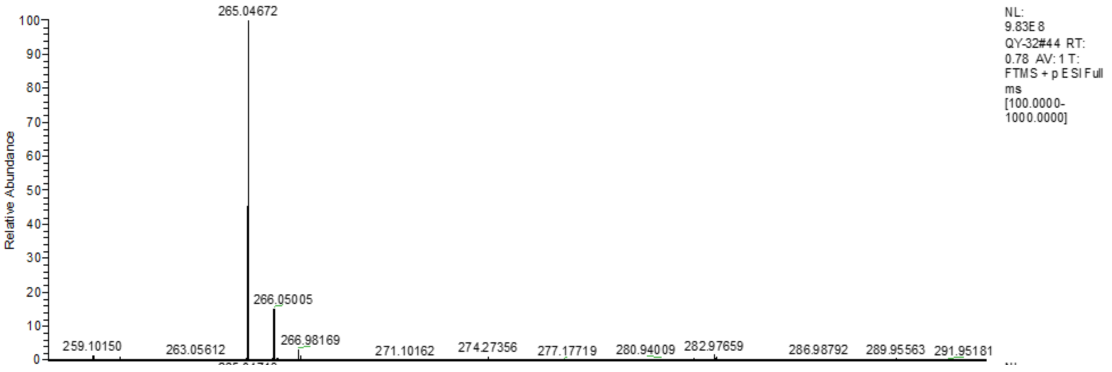

Figure S39. HRESIMS spectrum of **5**.

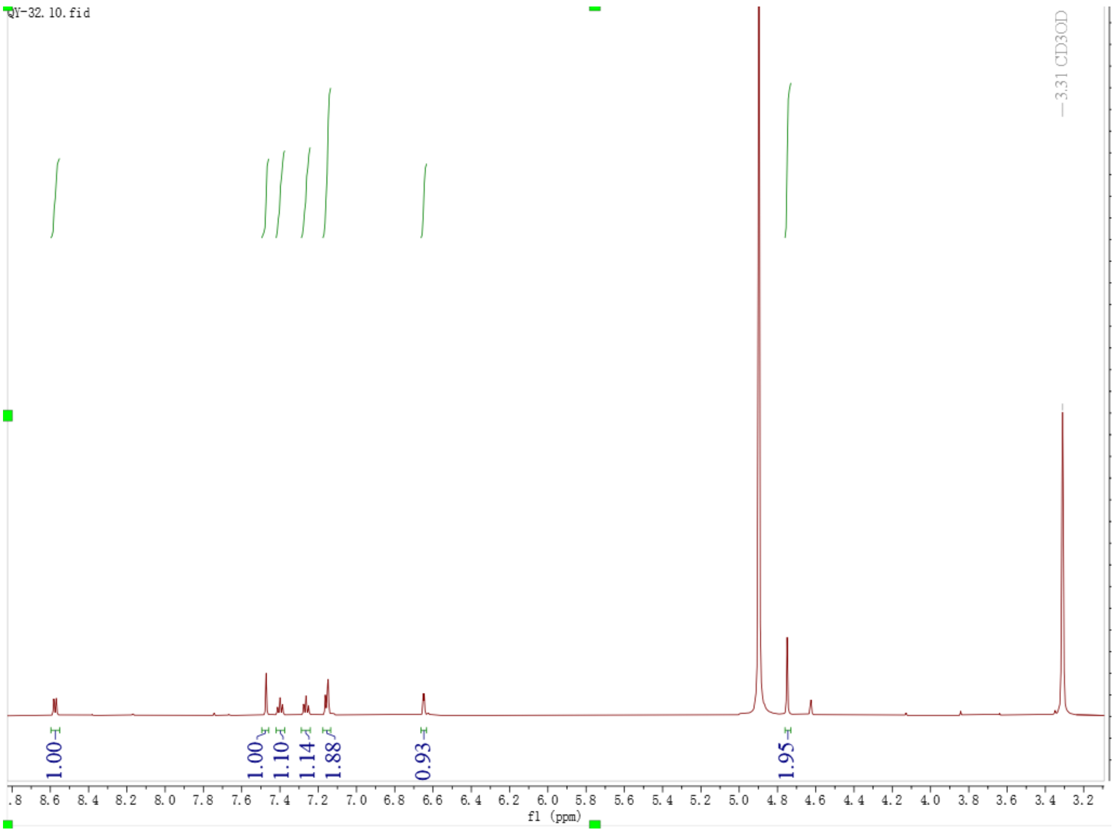

Figure S40. ^{1}H NMR spectrum of **5** in CD_3OD.

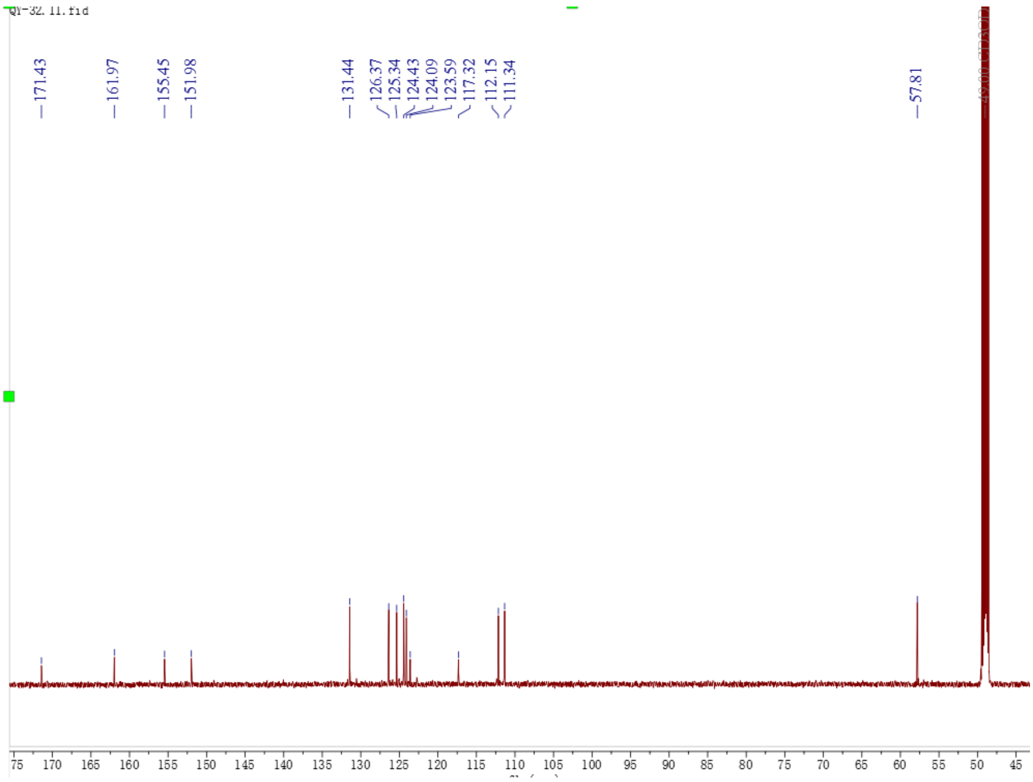

Figure S41. ^{13}C NMR spectrum of **5** in CD_3OD.

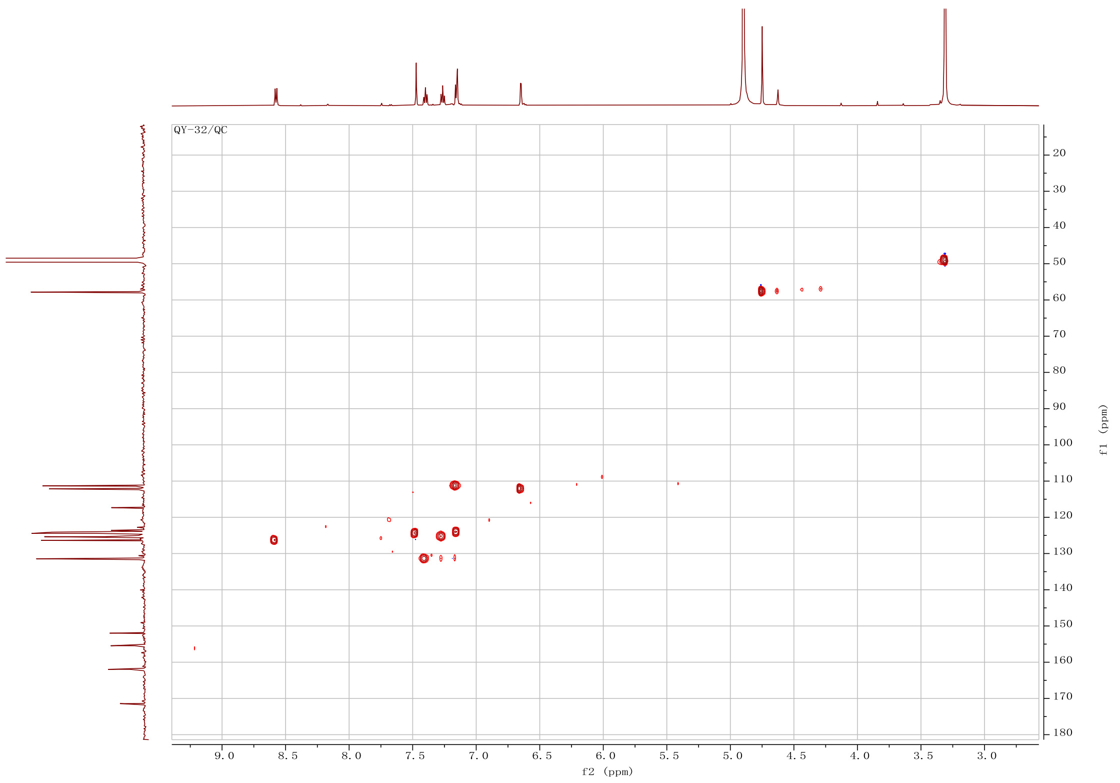

Figure S42. HSQC spectrum of **5** in CD_3OD.

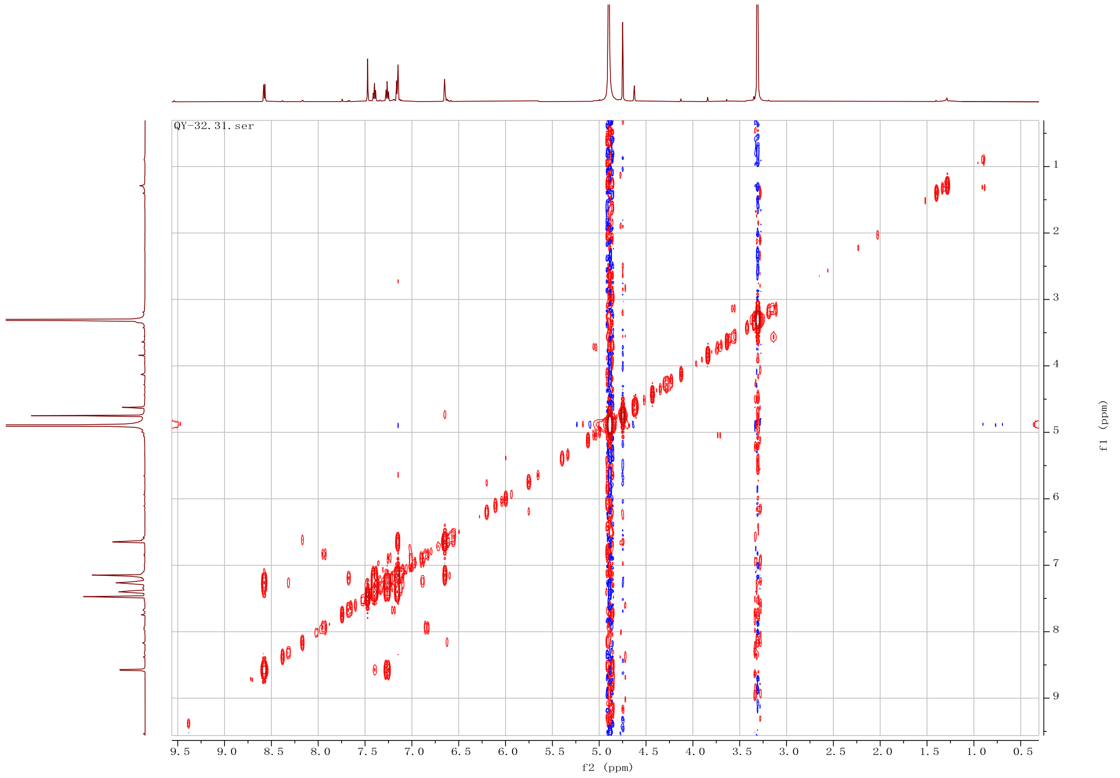

Figure S43. COSY spectrum of **5** in CD_3OD.

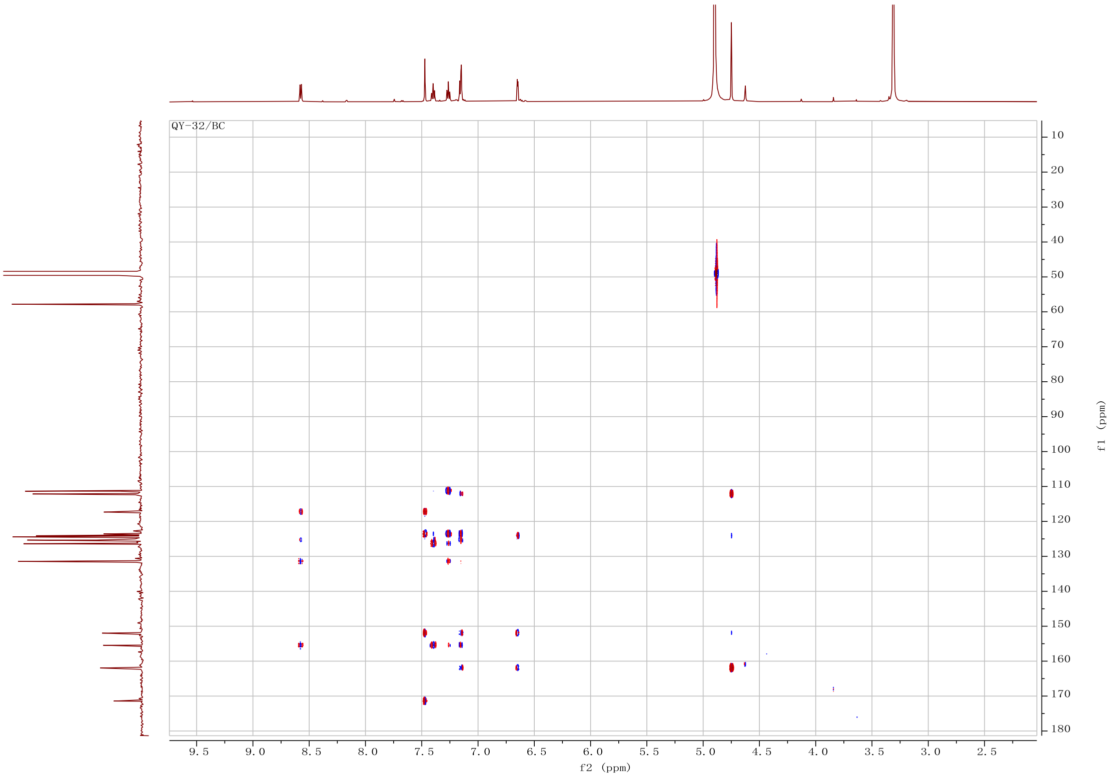

Figure S44. HMBC spectrum of **5** in CD_3OD.

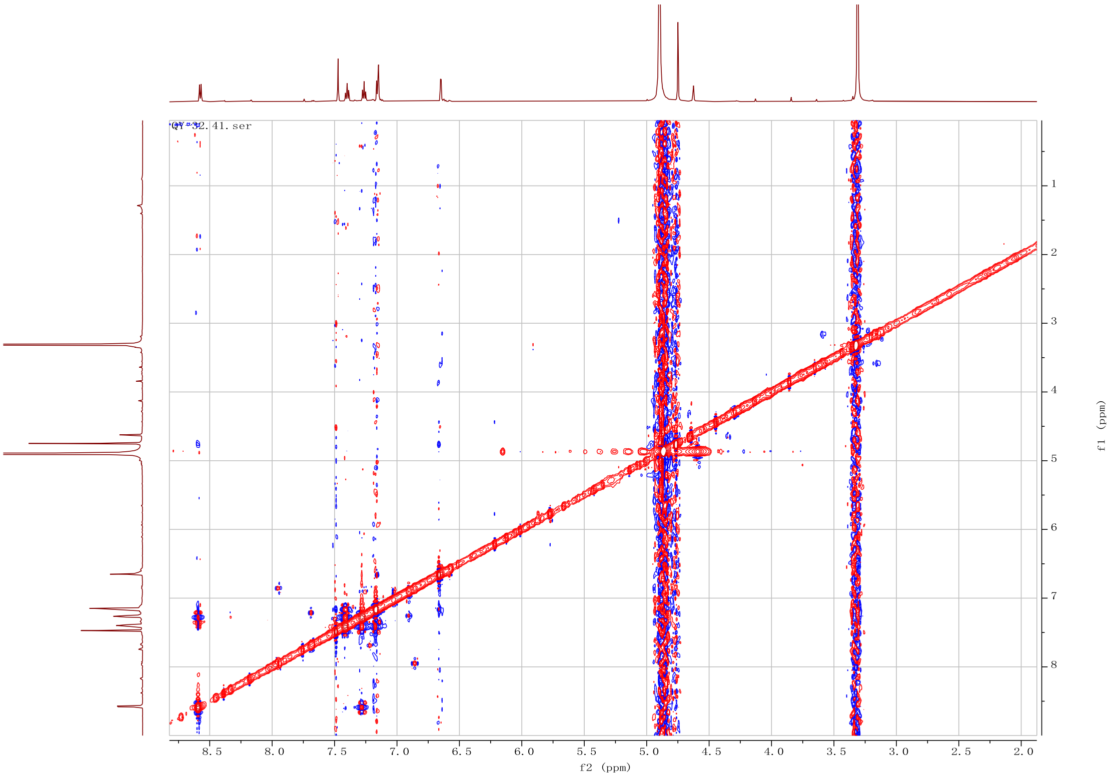

Figure S45. NOESY spectrum of **5** in CD_3OD.

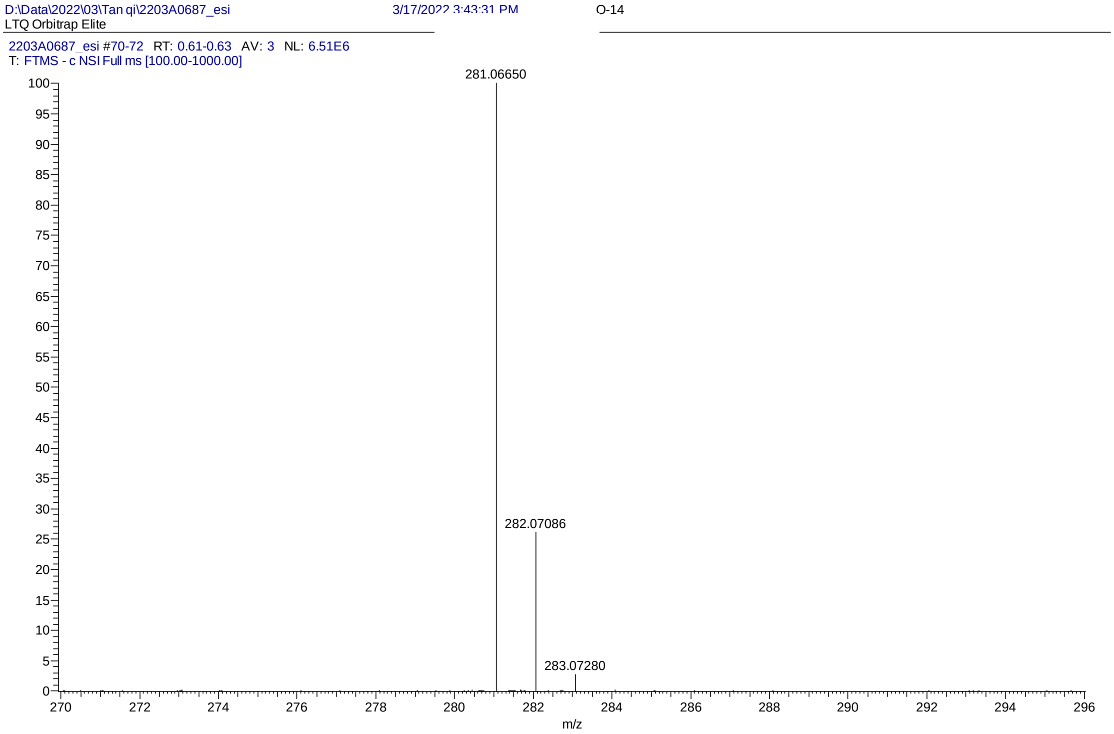

Figure S46. HRESIMS spectrum of **6**.

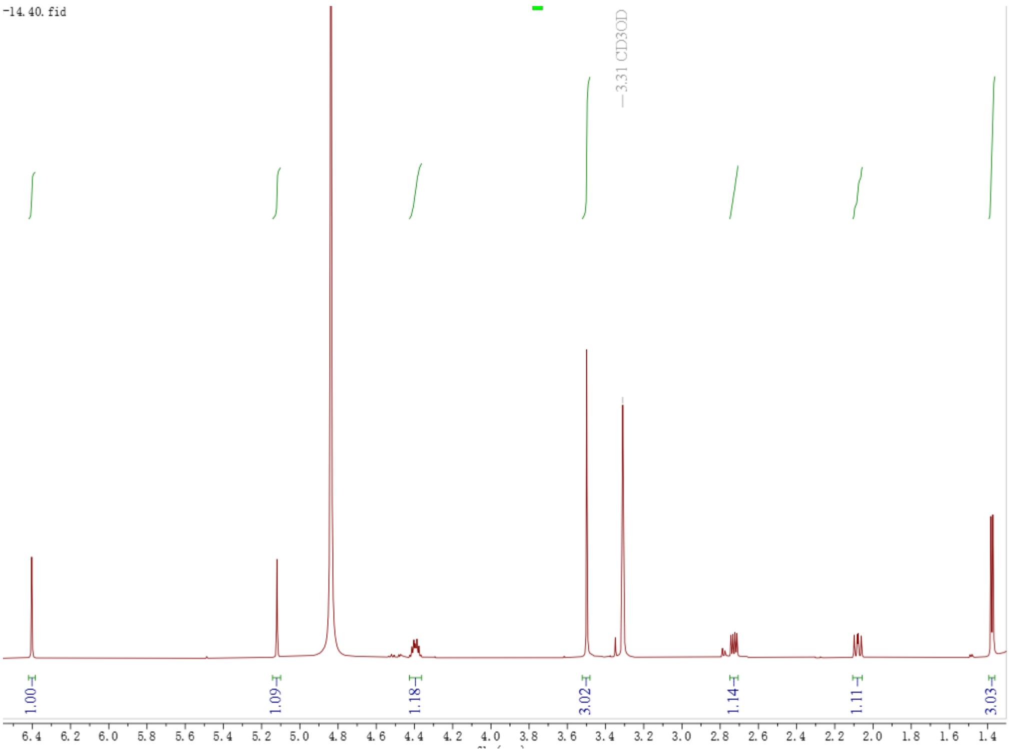

Figure S47. ^{1}H NMR spectrum of **6** in CD_3OD.

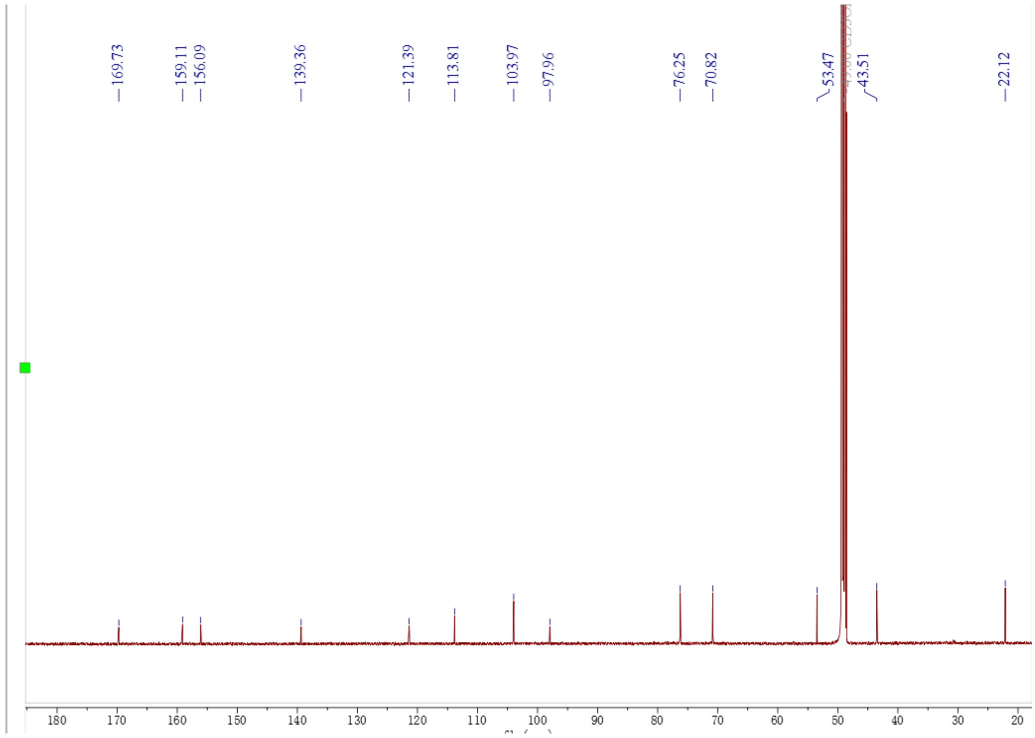

Figure S48. ^{13}C NMR spectrum of **6** in CD_3OD.

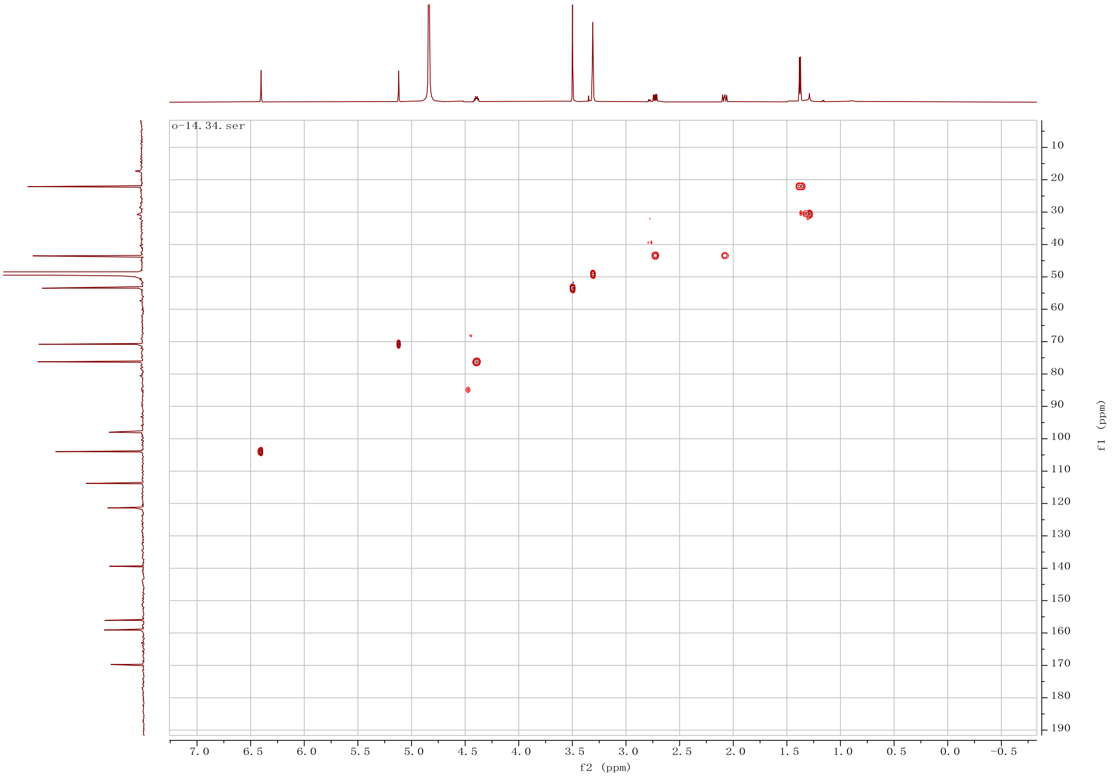

Figure S49. HSQC spectrum of **6** in CD_3OD.

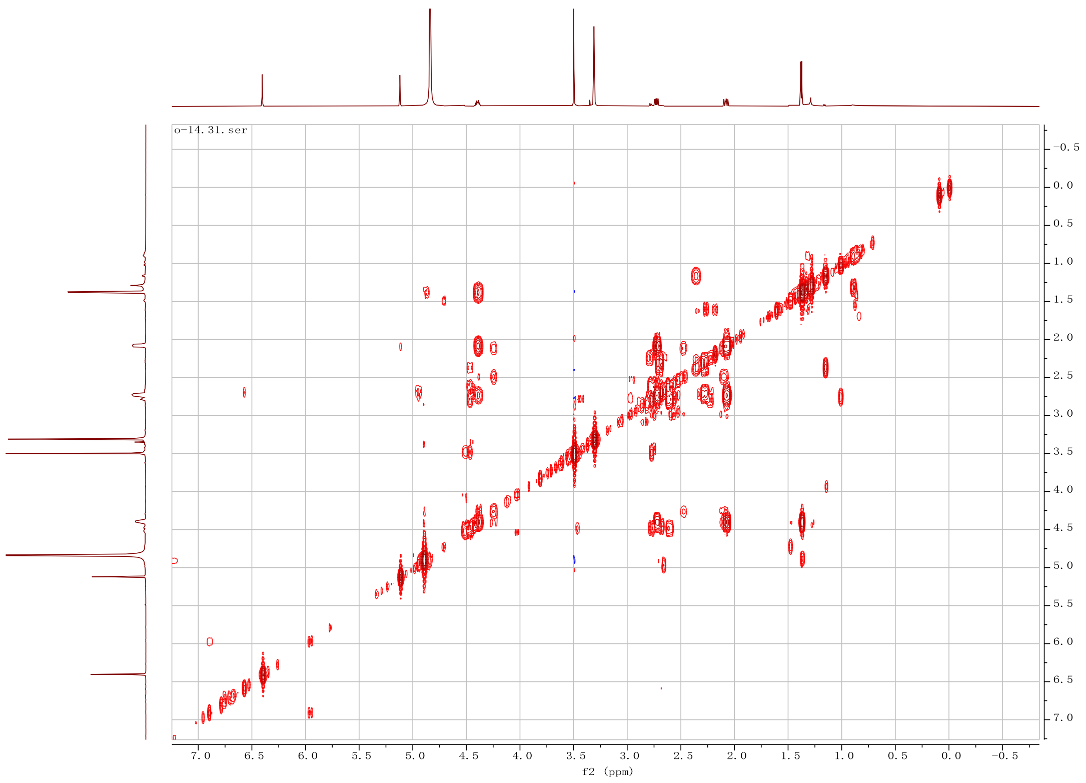

Figure S50. COSY spectrum of **6** in CD_3OD.

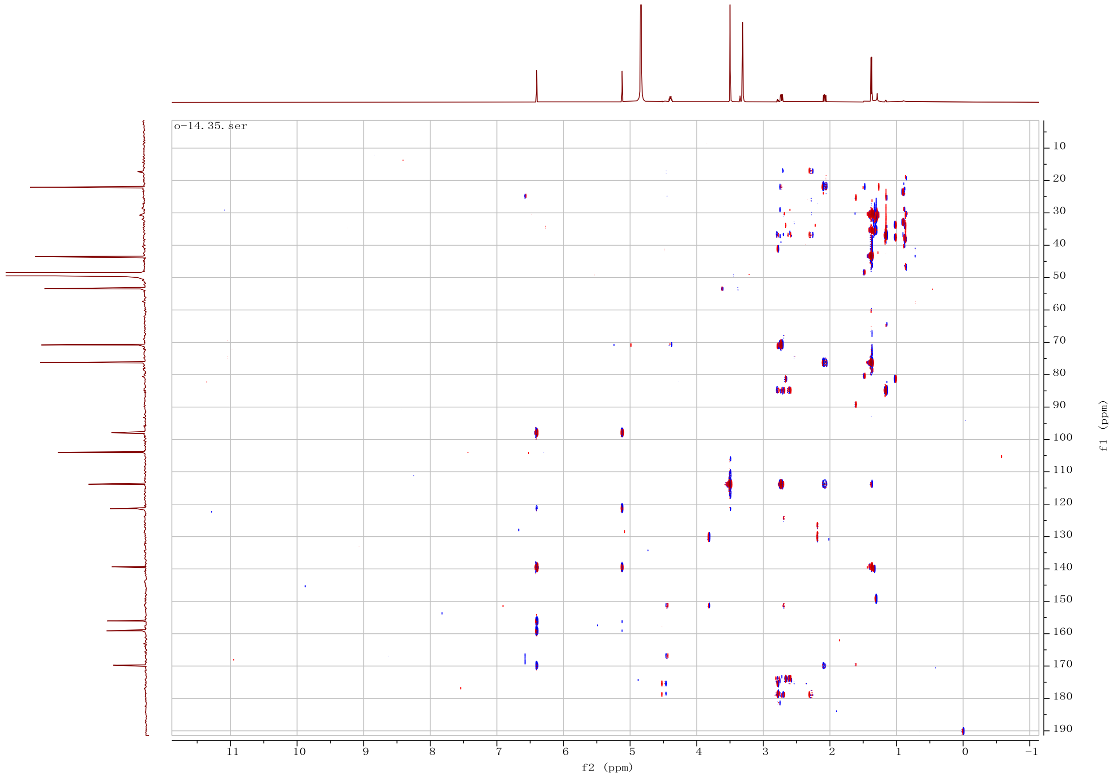

Figure S51. HMBC spectrum of **6** in CD_3OD.

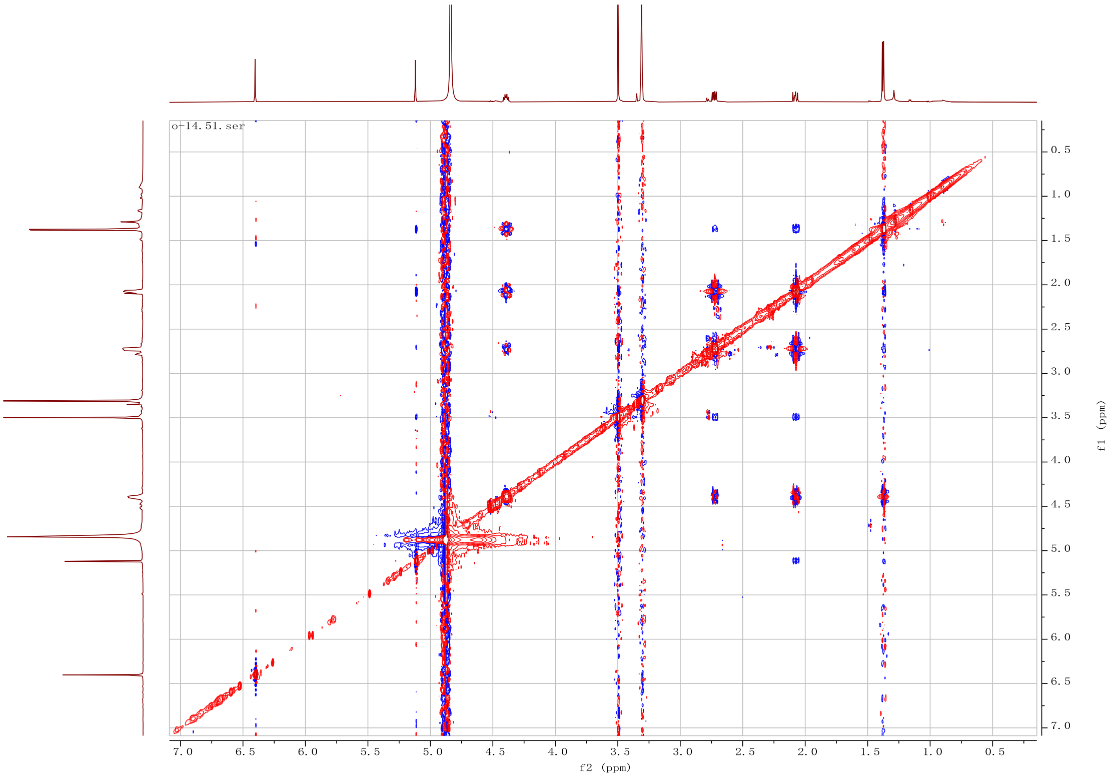

Figure S52. NOESY spectrum of **6** in CD_3OD.

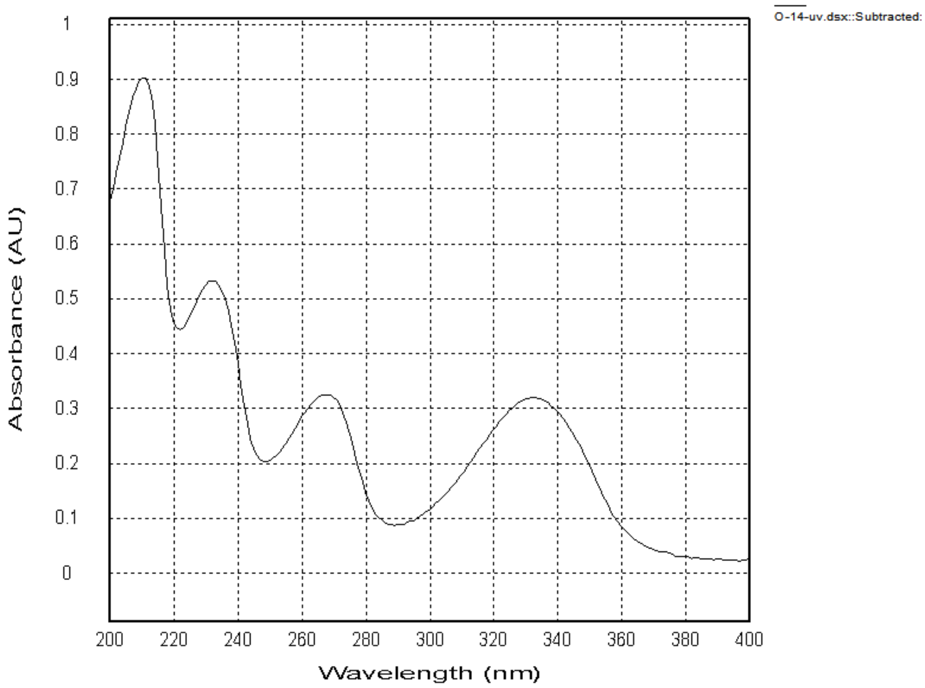

Figure S53. UV spectrum of **6**.

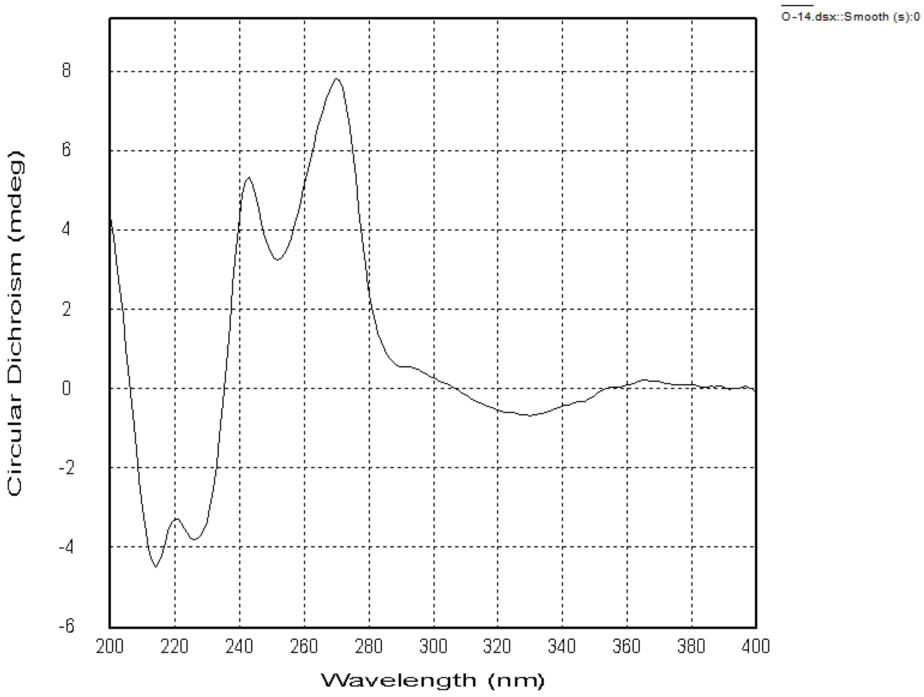

Figure S54. CD spectrum of **6**.

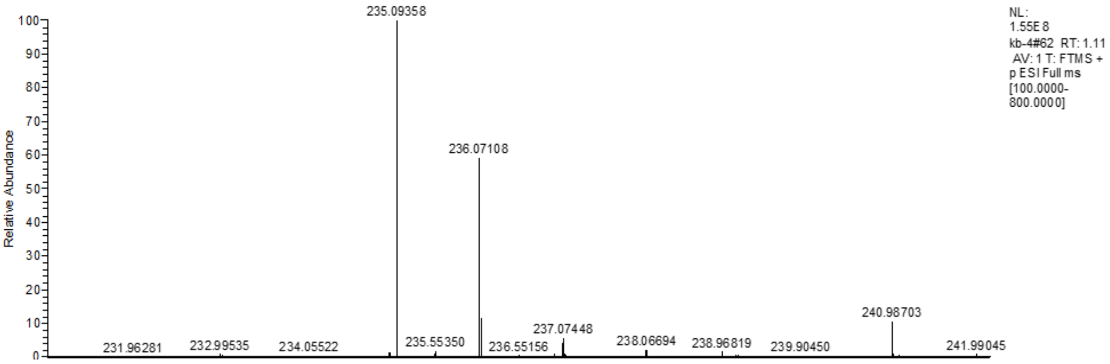

Figure S55. HRESIMS spectrum of **7**.

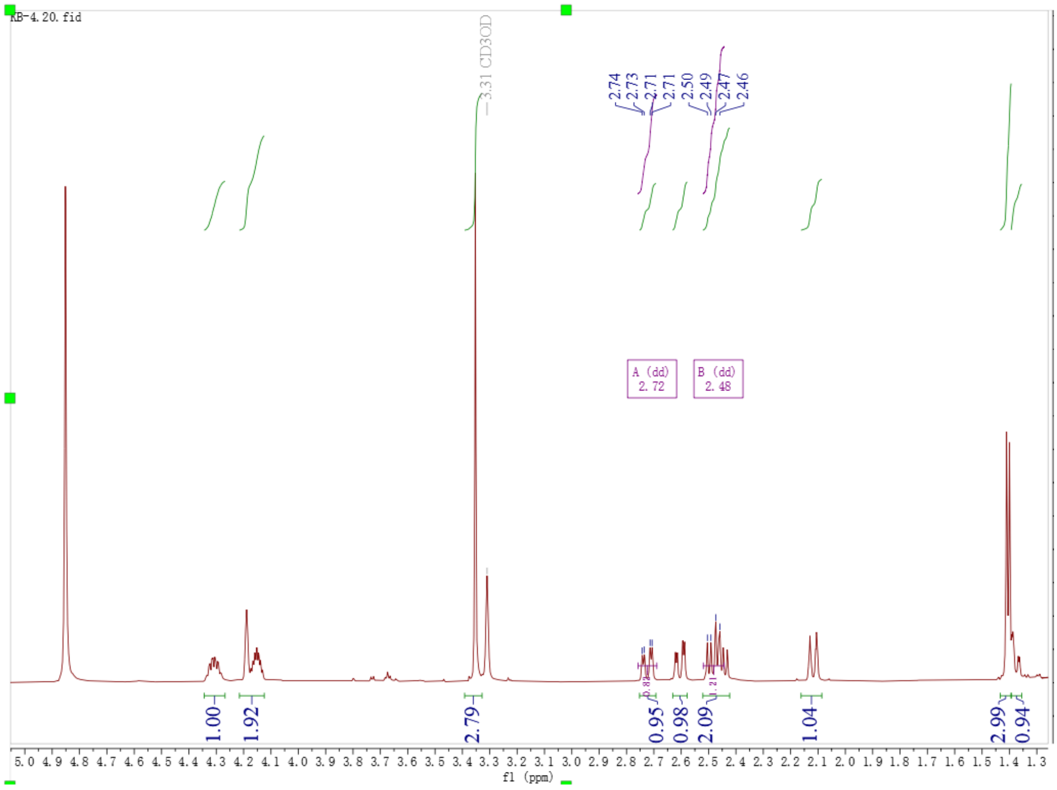

Figure S56. ^{1}H NMR spectrum of **7** in CD_3OD.

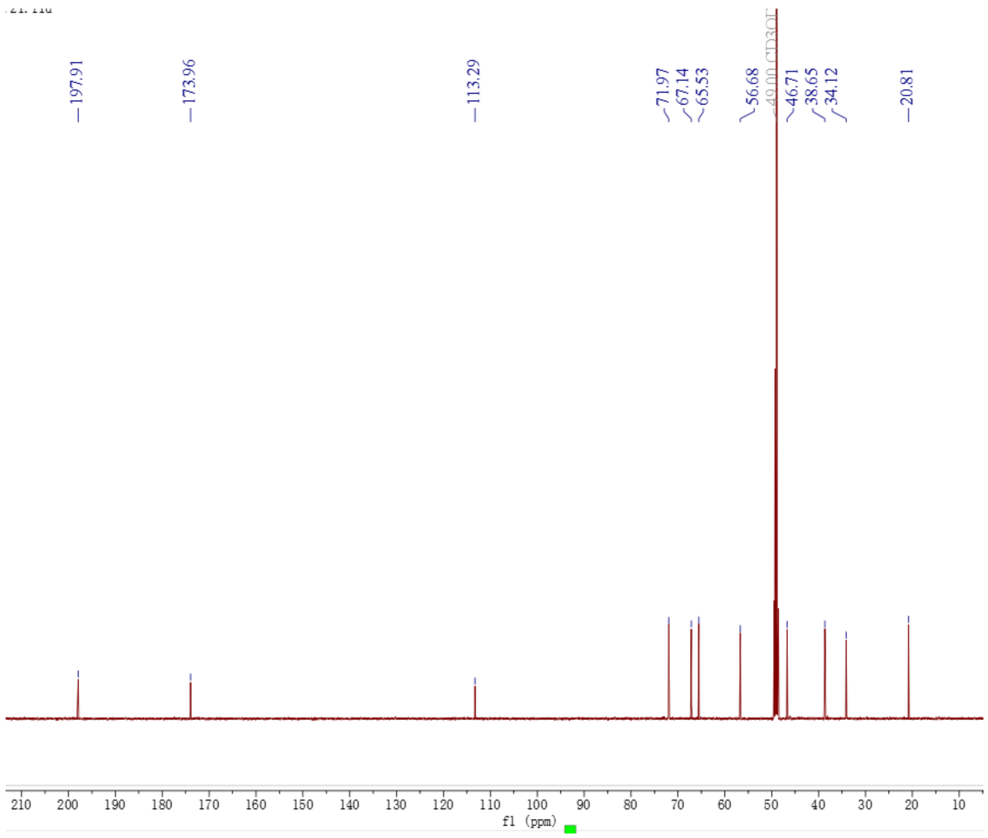

Figure S57. ^{13}C NMR spectrum of **7** in CD_3OD.

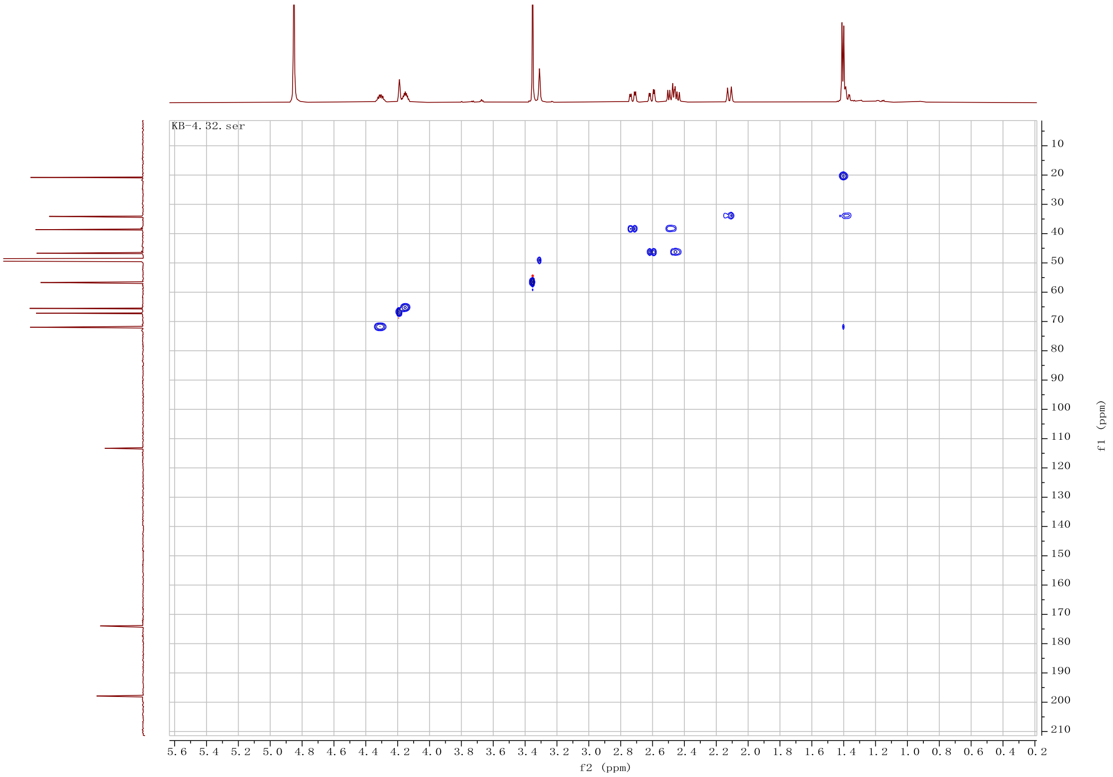

Figure S58. HSQC spectrum of **7** in CD_3OD.

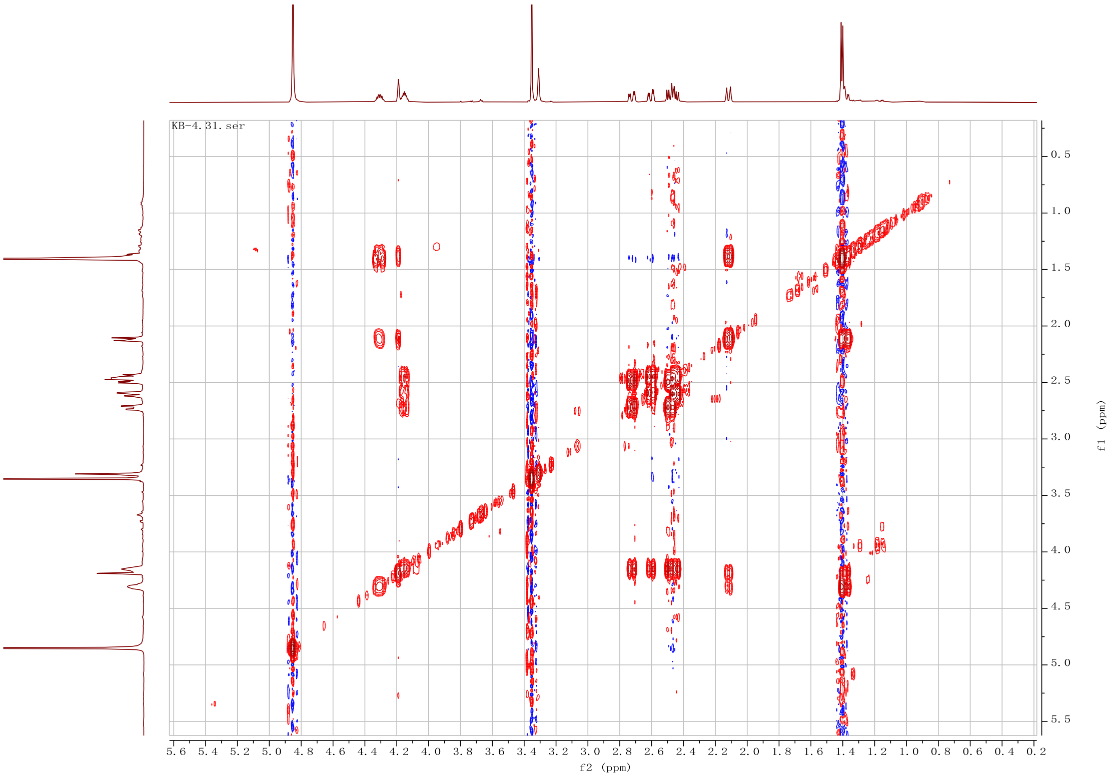

Figure S59. COSY spectrum of **7** in CD_3OD.

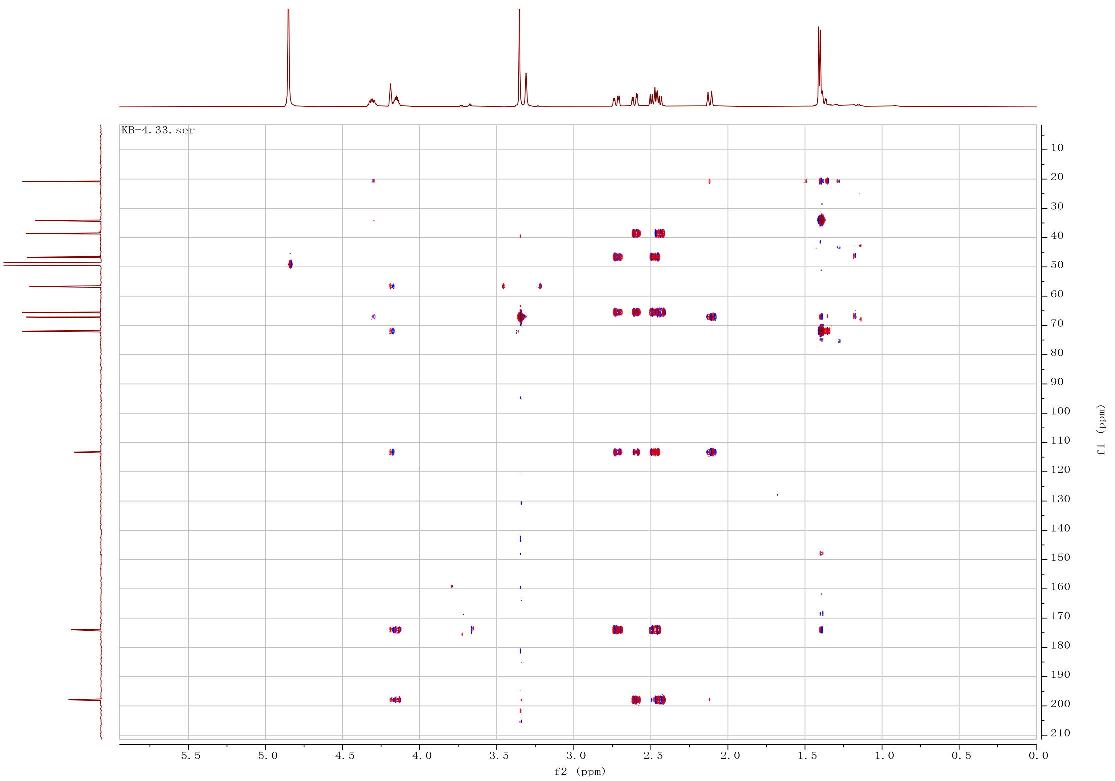

Figure S60. HMBC spectrum of **7** in CD_3OD.

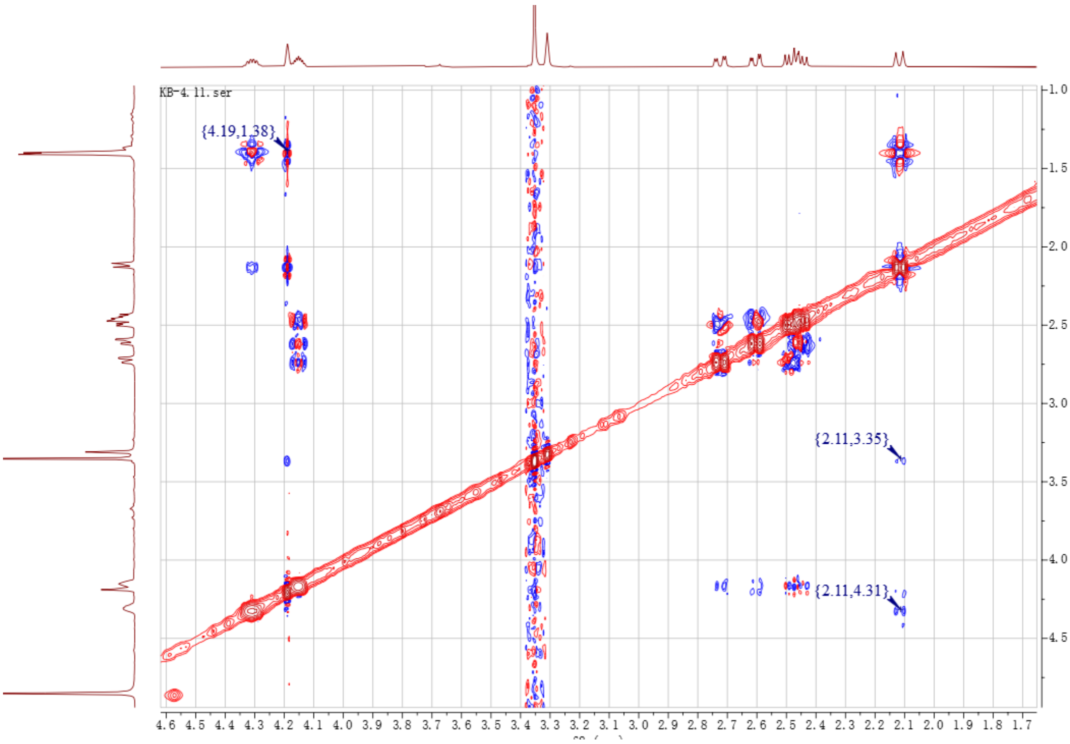

Figure S61. NOESY spectrum of **7** in CD_3OD.

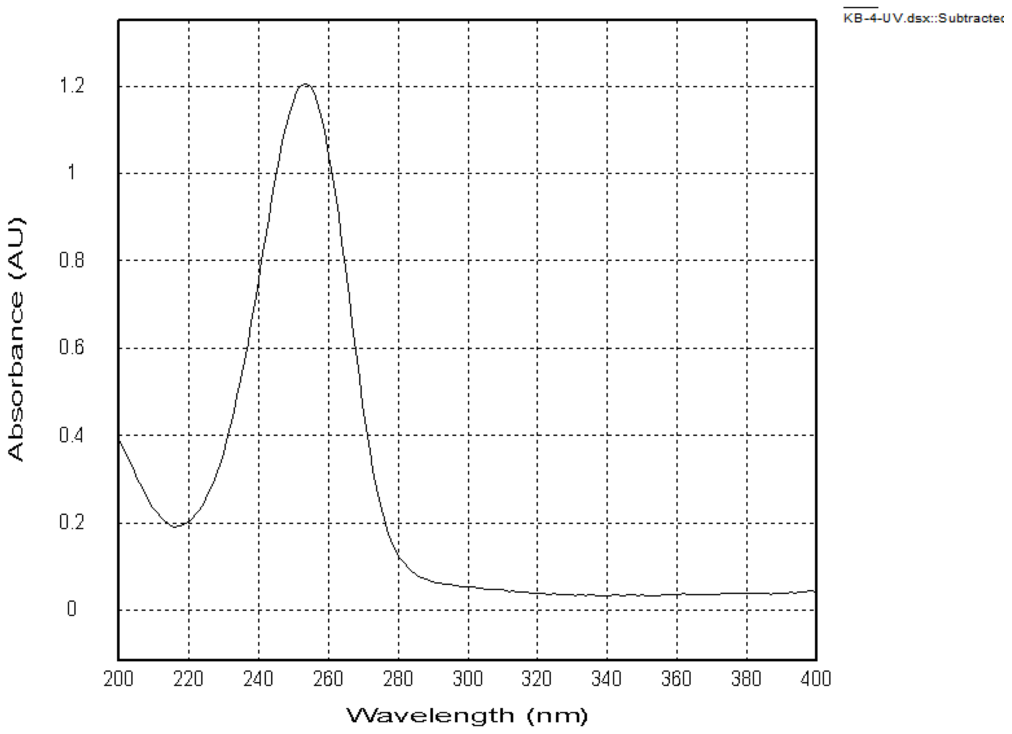

Figure S62. UV spectrum of **7**.

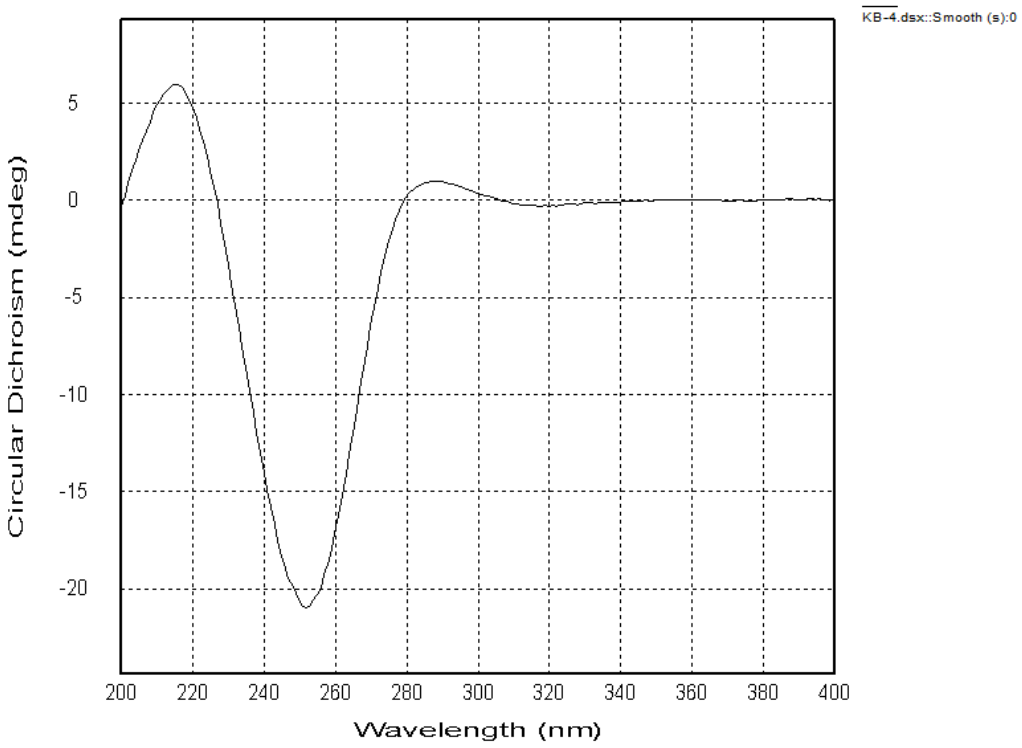

Figure S63. CD spectrum of **7**.

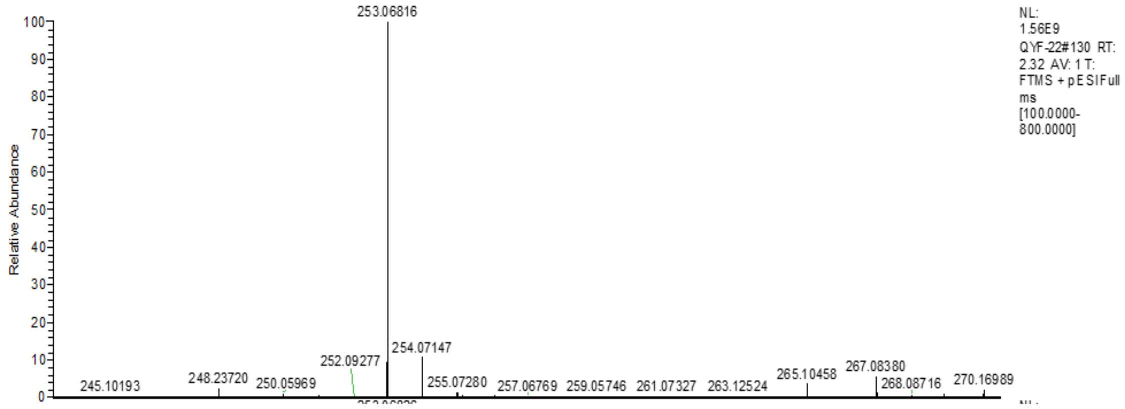

Figure S64. HRESIMS spectrum of **8**.

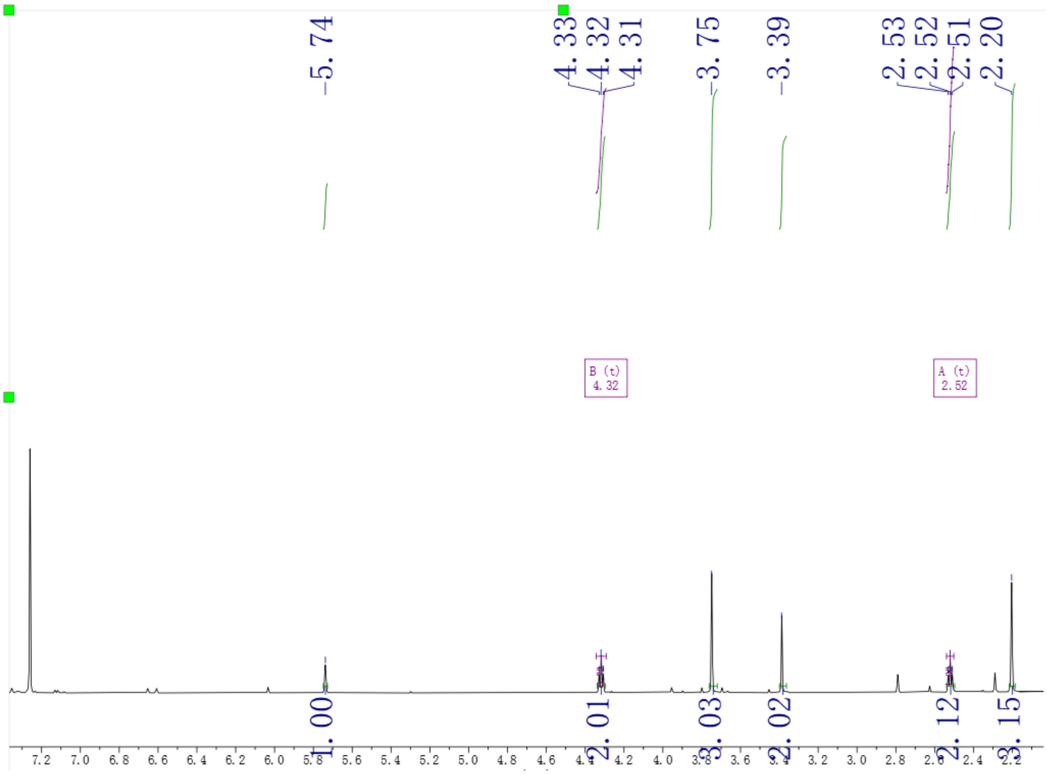

Figure S65. ^{1}H NMR spectrum of **8** in $CDCl_3$.

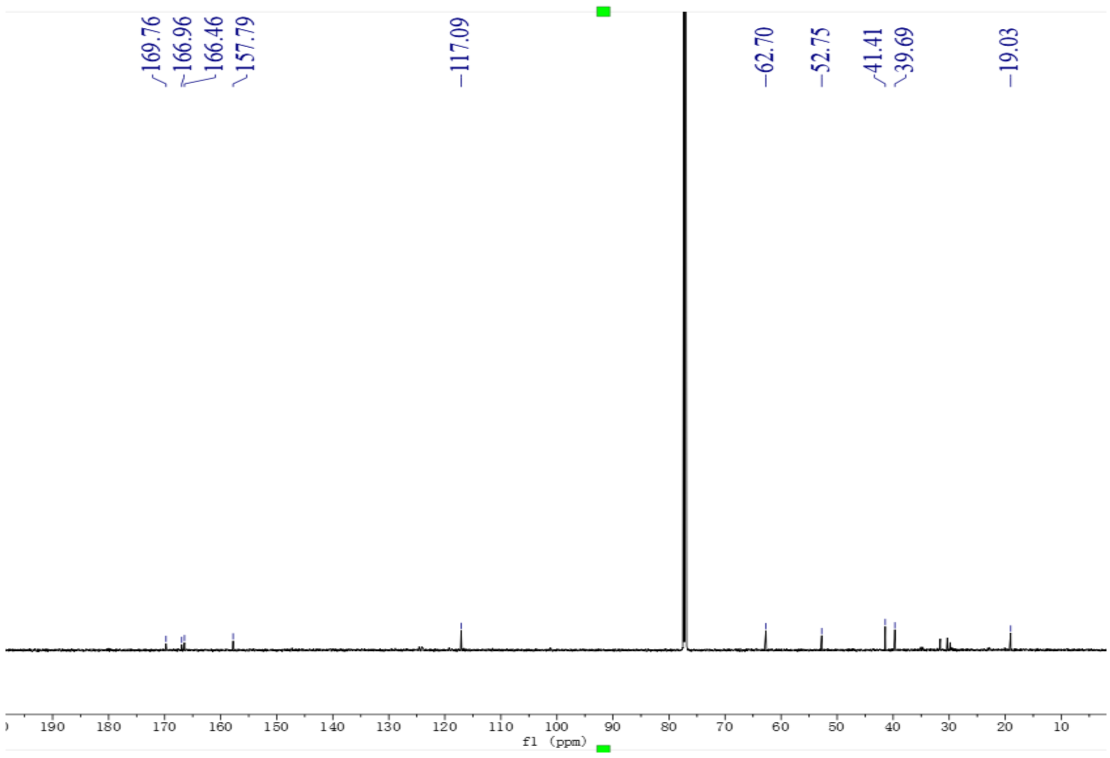

Figure S66. ^{13}C NMR spectrum of **8** in $CDCl_3$.

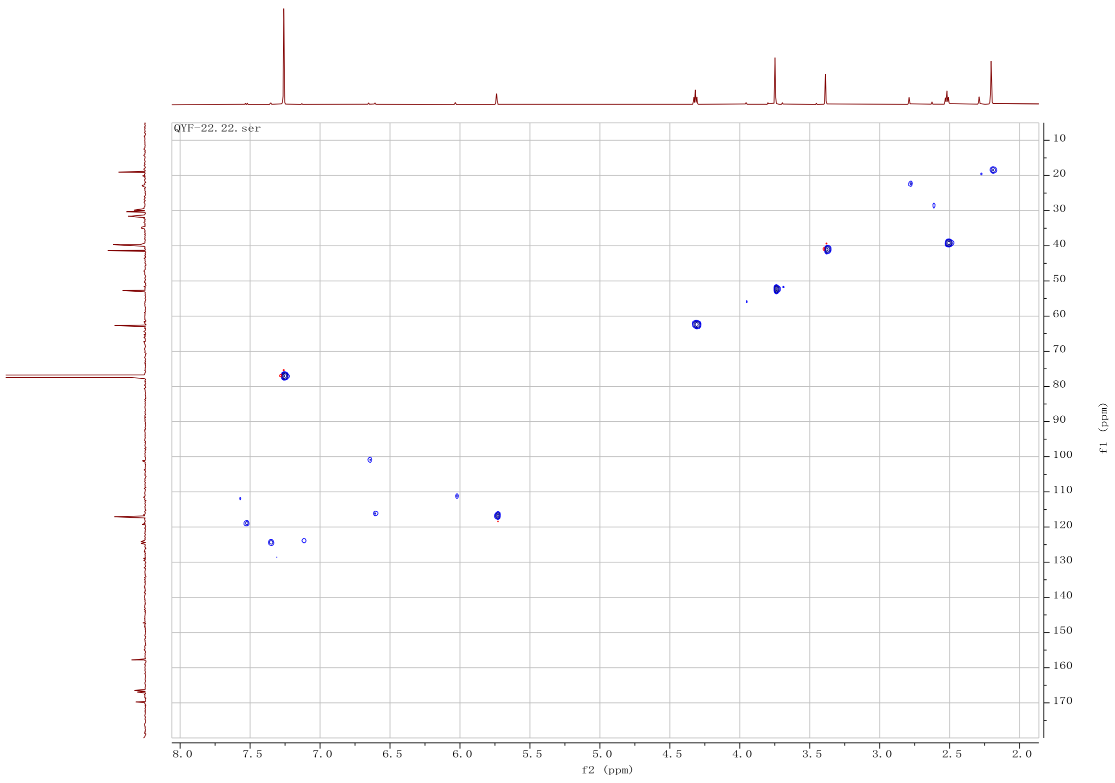

Figure S67. HSQC spectrum of **8** in $CDCl_3$.

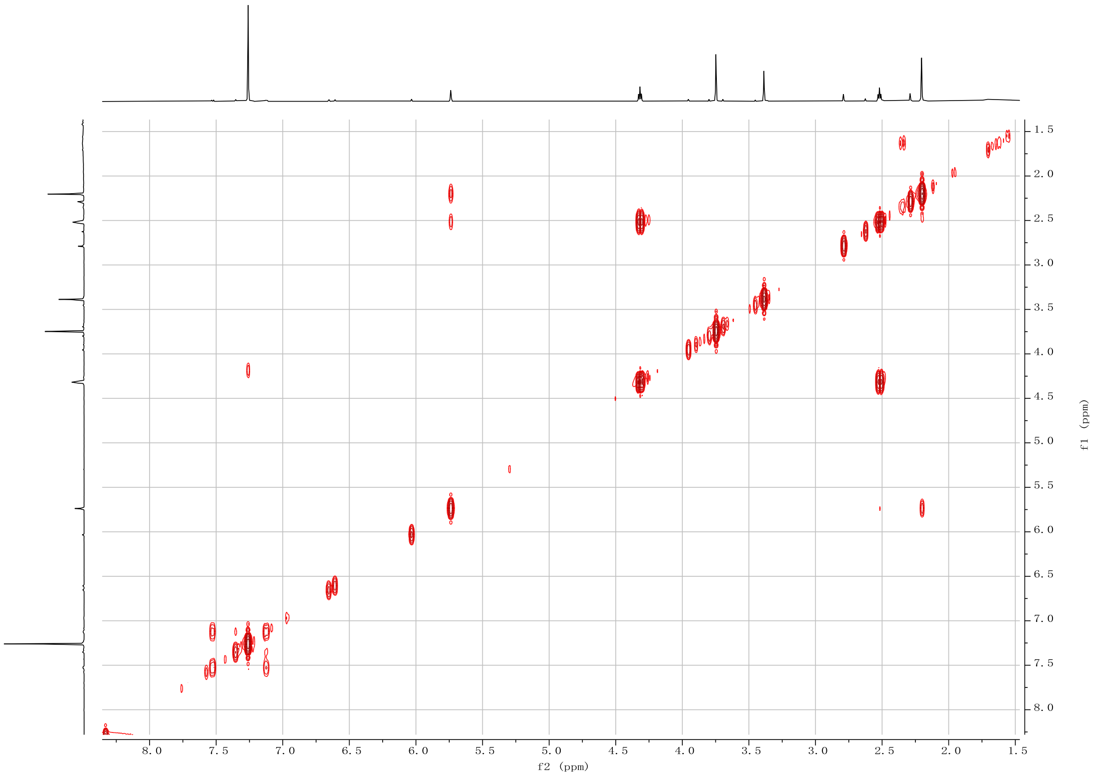

Figure S68. COSY spectrum of **8** in $CDCl_3$.

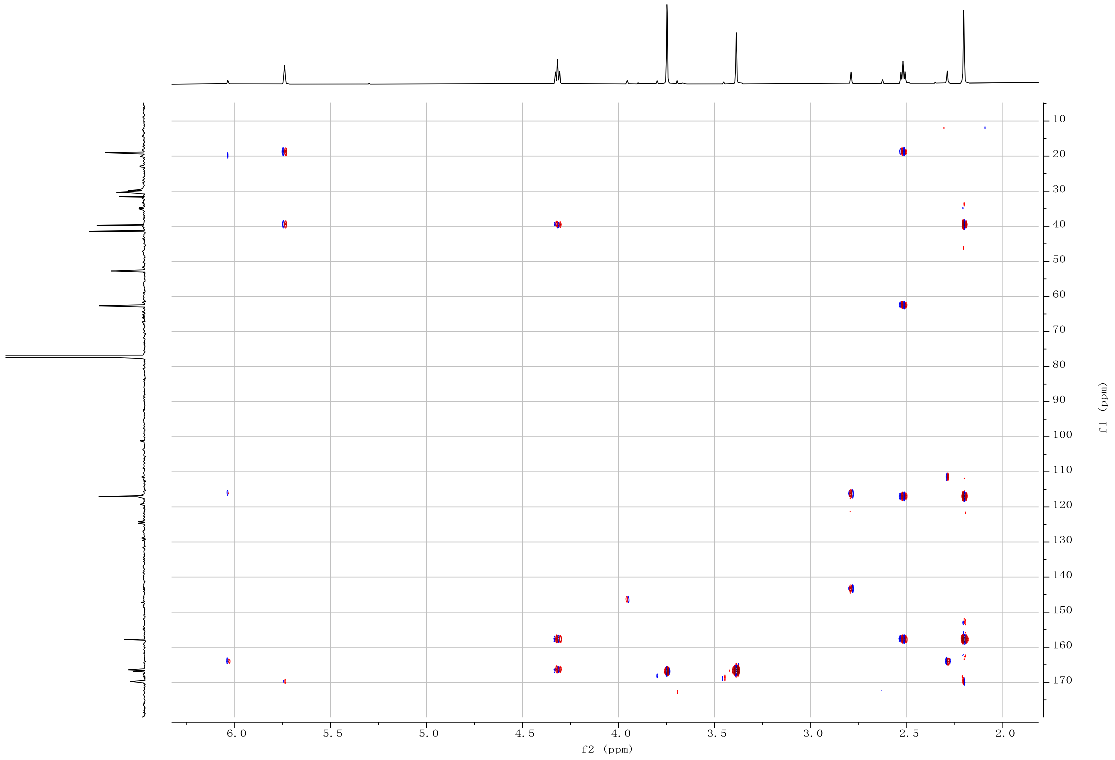

Figure S69. HMBC spectrum of **8** in $CDCl_3$.

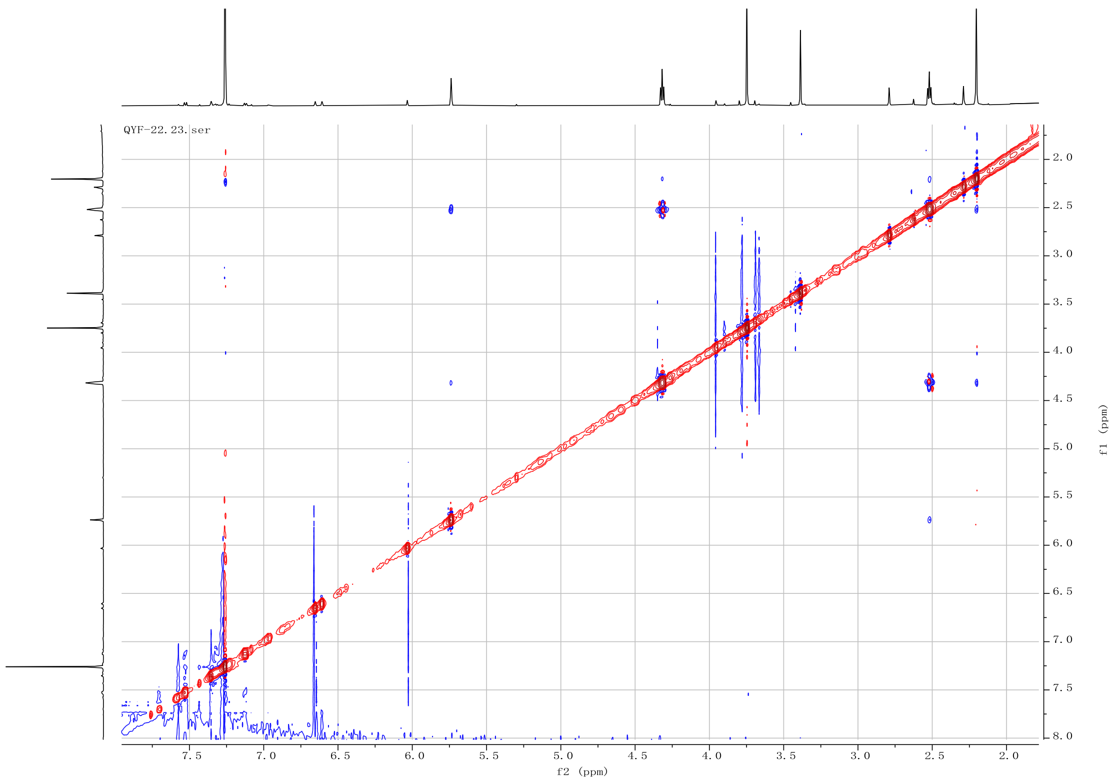

Figure S70. NOESY spectrum of **8** in $CDCl_3$.

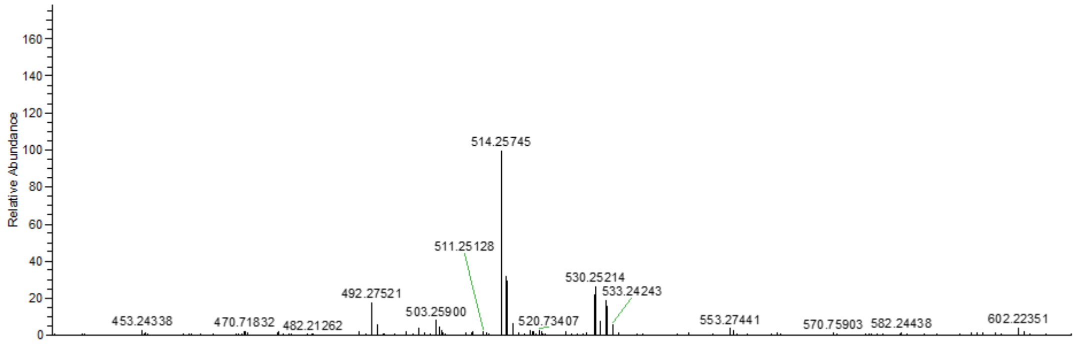

Figure S71. HRESIMS spectrum of **9** (*m*/*z* [M + Na]$^+$).

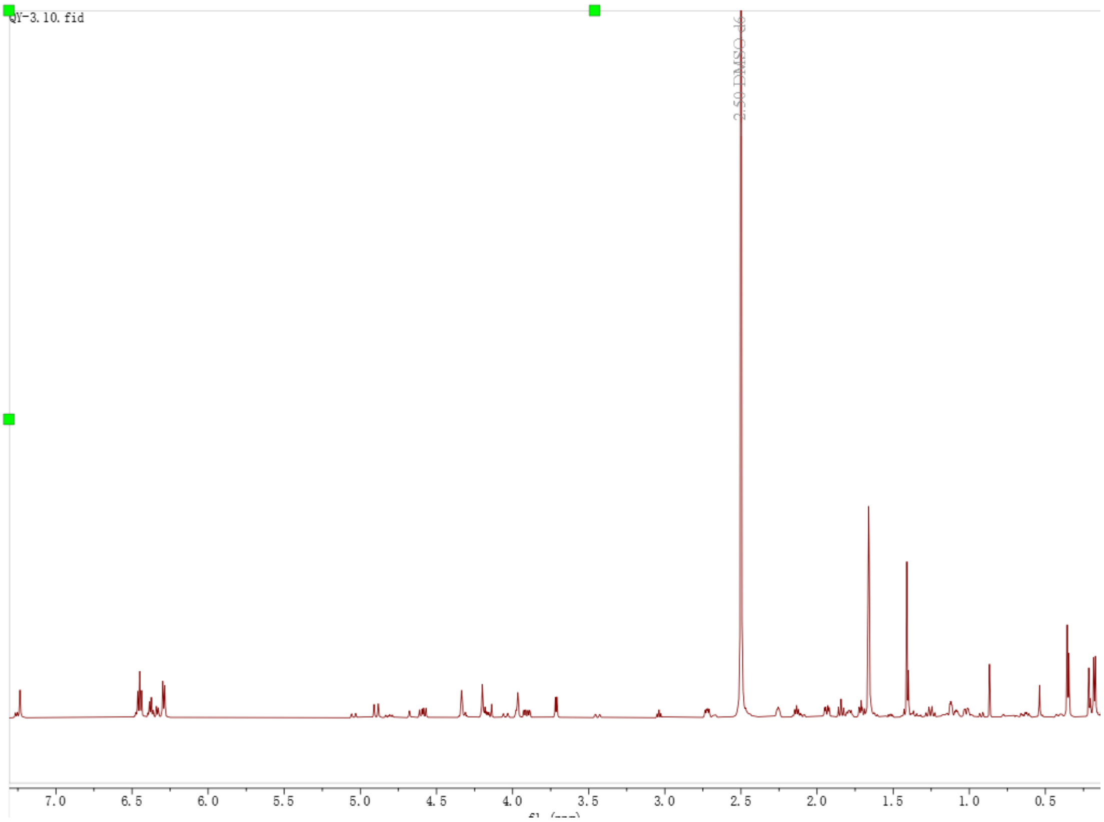

Figure S72. ^{1}H NMR spectrum of **9** in DMSO-d_6.

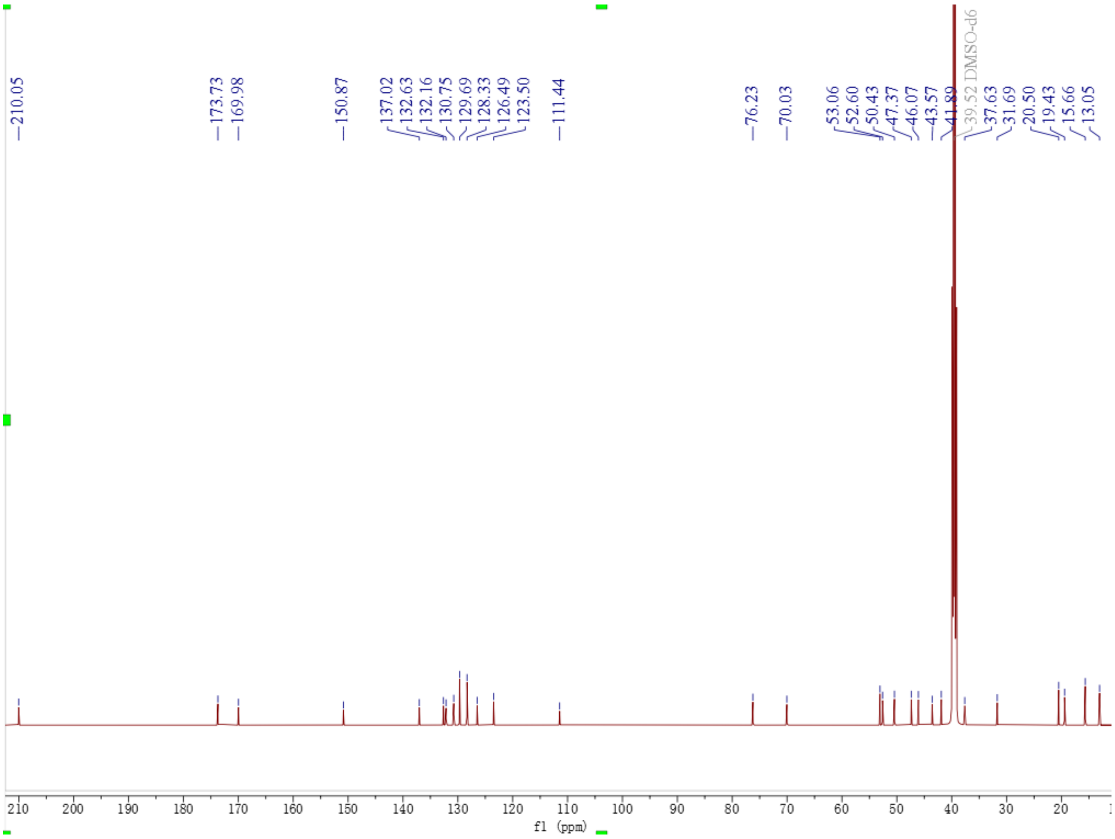

Figure S73. ^{13}C NMR spectrum of **9** in DMSO- d_6.

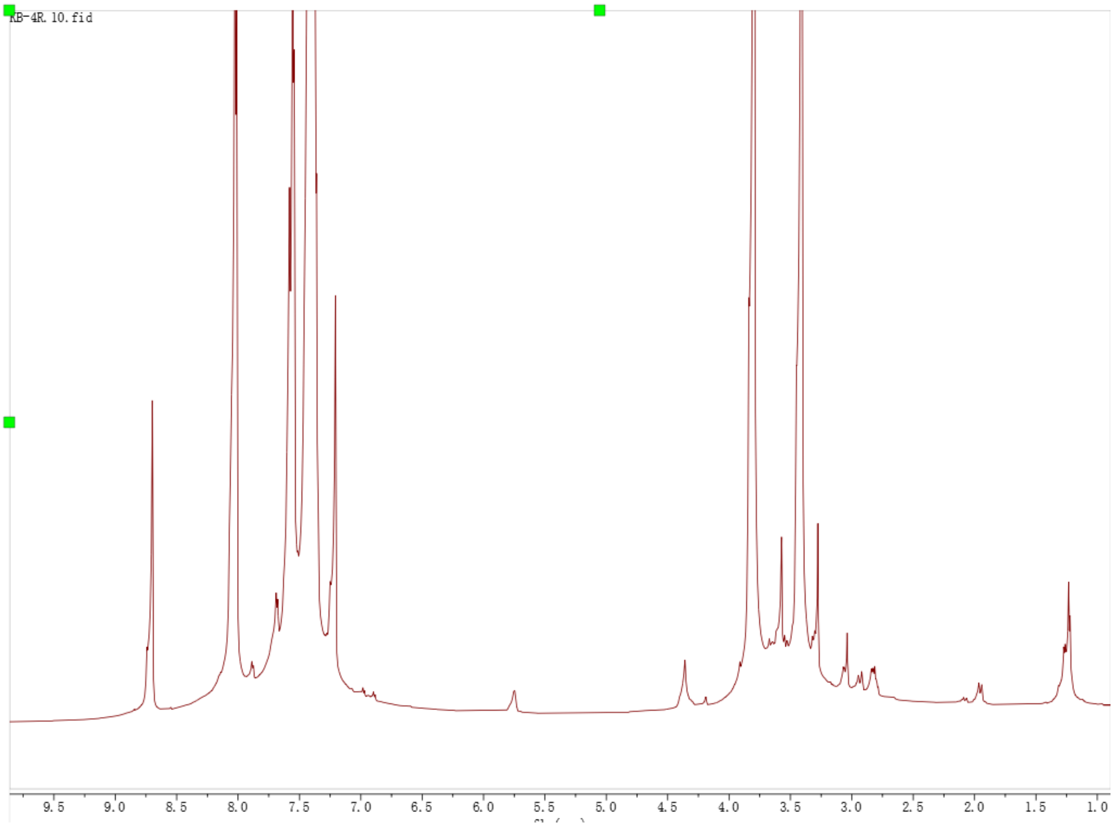

Figure S74. ^{1}H NMR spectrum of (*R*)-MTPA ester of **7**.

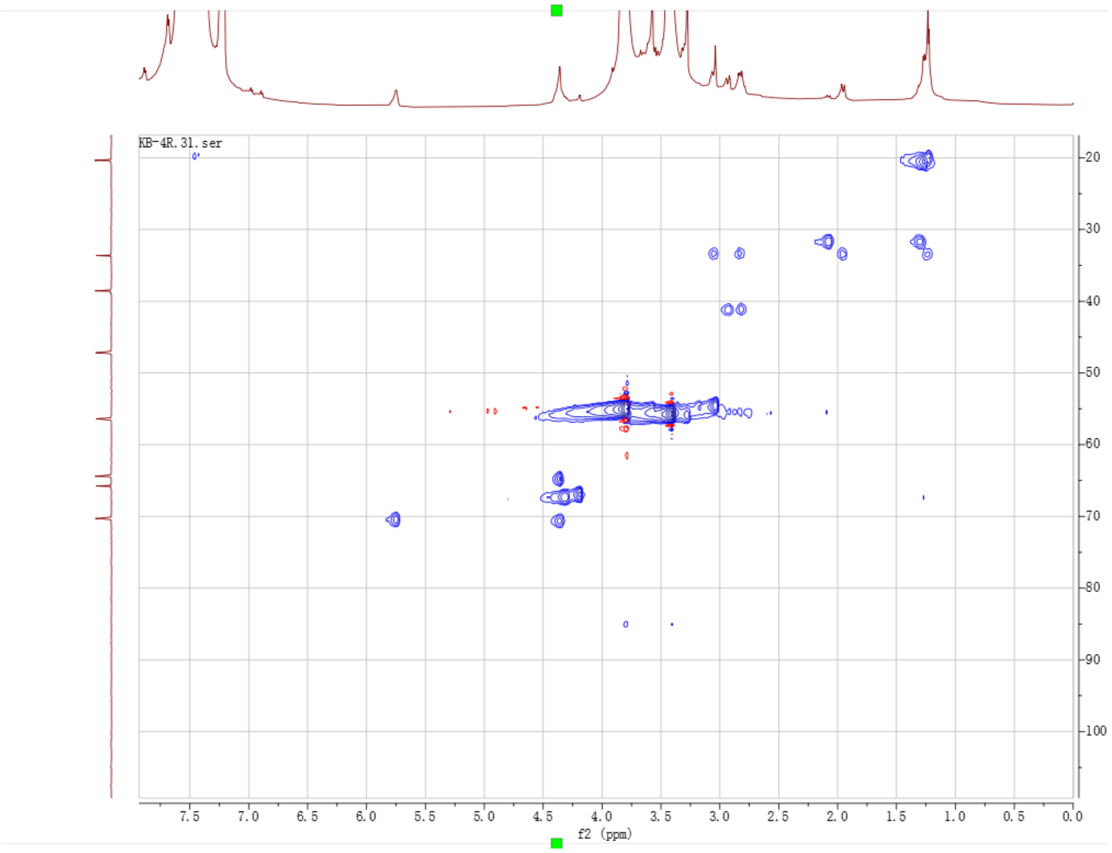

Figure S75. HSQC spectrum of (*R*)-MTPA ester of **7**.

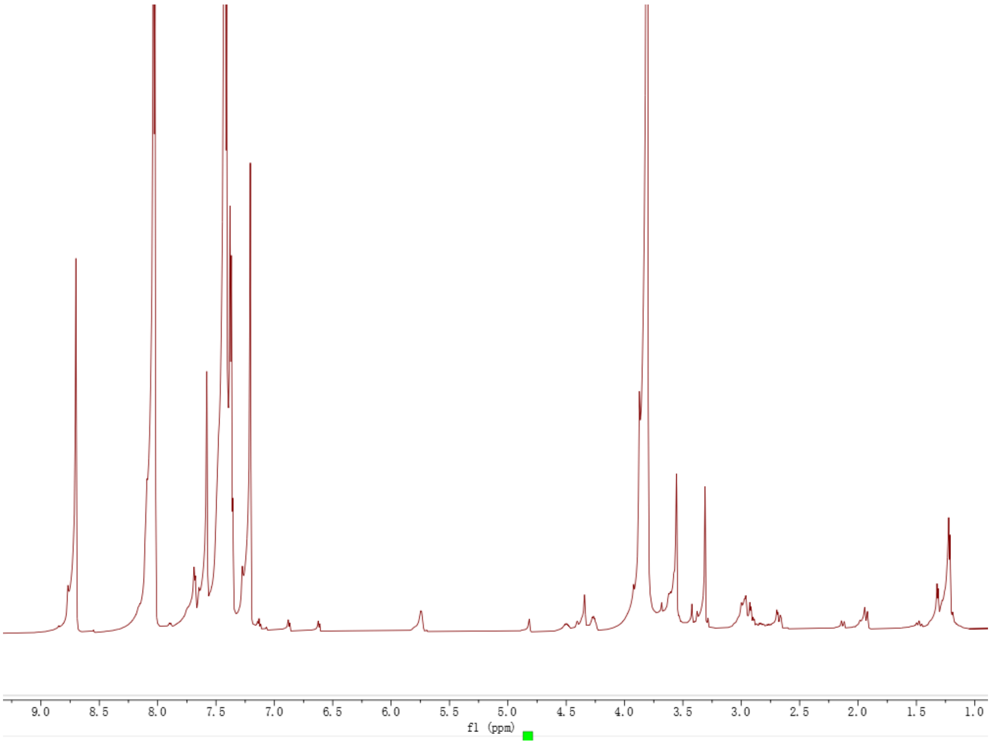

Figure S76. ^{1}H NMR spectrum of (*S*)-MTPA ester of **7**.

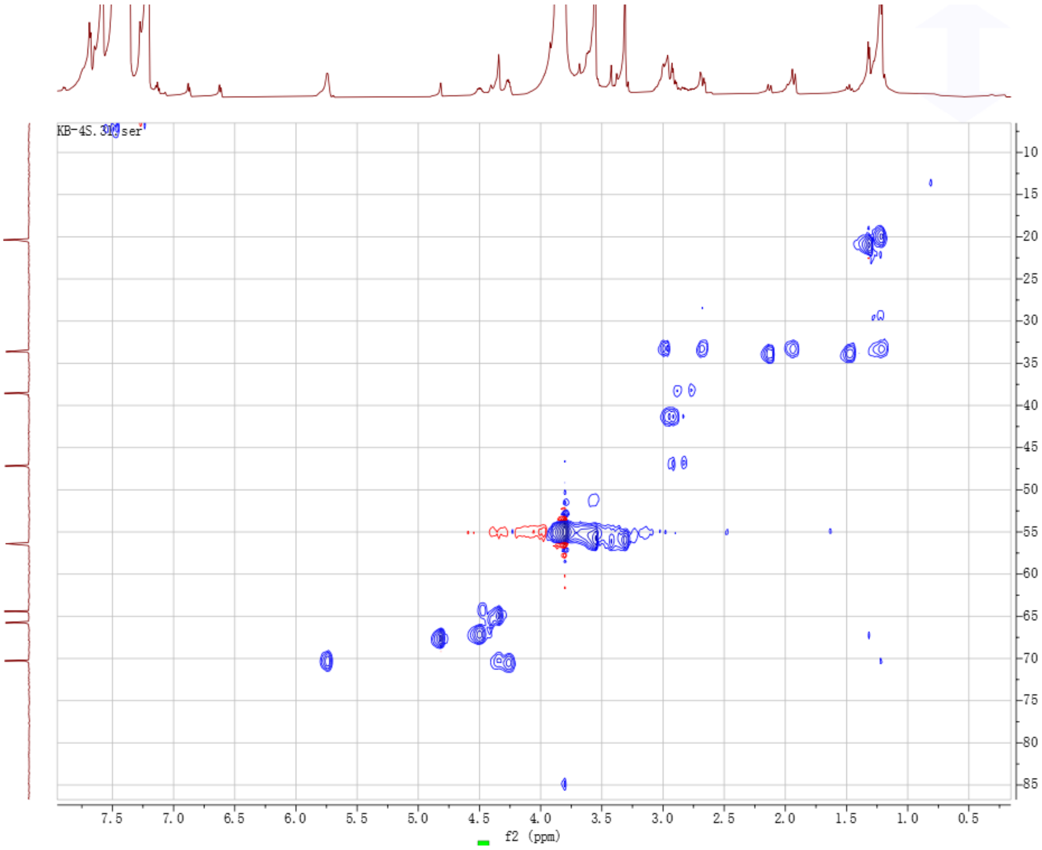

Figure S77. HSQC spectrum of (*S*)-MTPA ester of **7**.

Calculation of 3*S*, 4*R*, 7S, 8*R*, 9*R*, 16*S*, 18*R*, 21*R*

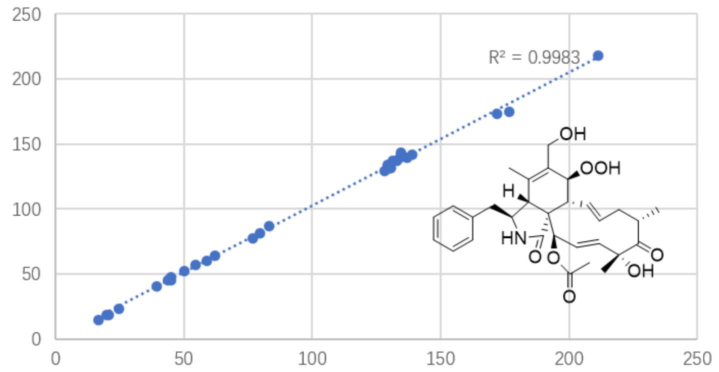

Calculation of 3*S*, 4*R*, 7S, 8*R*, 9*R*, 16*S*, 18*R*, 21*S*

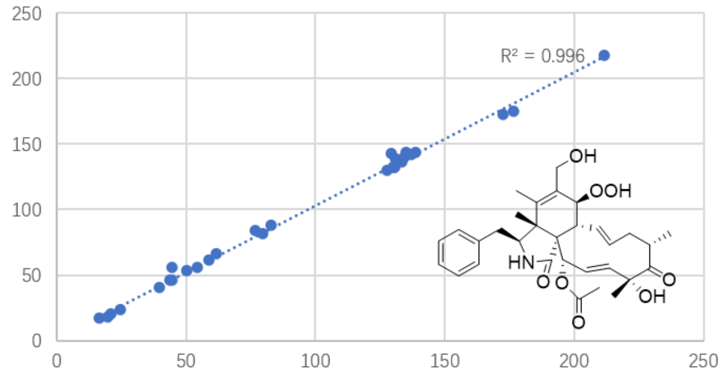

Figure S78. Comparison of the experimental ^{13}C NMR data of compound **1** and the calculated chemical shifts of (3*S*, 4*R*, 7S, 8*R*, 9*R*, 16*S*, 18*R*, 21*R*-**1**, and 3*S*, 4*R*, 7S, 8*R*, 9*R*, 16*S*, 18*R*, 21*S*-**1**).

	Isomer 1	Isomer 2
sDP4+ (H data)	100.00%	0.00%
sDP4+ (C data)	100.00%	0.00%
sDP4+ (all data)	100.00%	0.00%
uDP4+ (H data)	100.00%	0.00%
uDP4+ (C data)	91.33%	8.67%
uDP4+ (all data)	100.00%	0.00%
DP4+ (H data)	100.00%	0.00%
DP4+ (C data)	100.00%	0.00%
DP4+ (all data)	100.00%	0.00%

Figure S79. DP4+ analysis of compound **1**.

3*S*, 4*R*, 7S, 8*R*, 9*R*, 16*S*, 18*R*, 21*R*-**1** (**1**)

3*S*, 4*R*, 7S, 8*R*, 9*R*, 16*S*, 18*R*, 21*S*-**1** (**1'**)

Conformational searches for the molecules **1** and **1'** were carried out using the MMFF94s force field by the MOE software (Chemical Computing Group ULC). For molecules **1** and **1'**, totally 58 stable conformers for **1** with relative energy within a 10.0 kcal/mol energy window and 50 stable conformers for **1'** were recorded, respectively. DFT calculations were used to optimize the conformers at the B3LYP/6-31G(d) and B3LYP/6-311+G(d) levels, respectively. The C/H NMR calculations for the stable conformers were performed by Gaussian 09 (Gaussian Inc.) software. TD-DFT at the B3LYP/6-311+G(d,p) level in the gas phase was used for C/H NMR calculations with a total of 60 excited states for both **1** and **1'**. The NMR data of **1** and **1'** were compared with the experimental data by means of the DP4+ table. The outcome was that the similarity between the configuration of **1** and the experimental data was 100%, while that of **1'** was 0%.

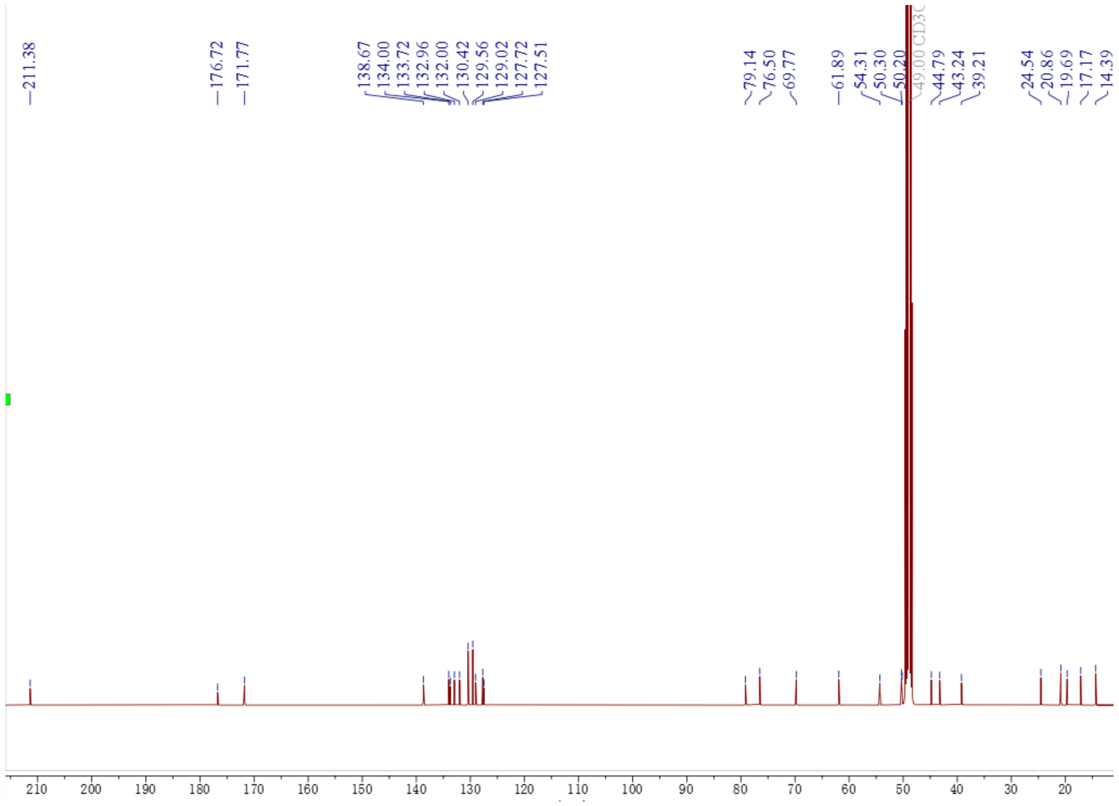

Figure S80. ^{13}C NMR spectrum of **11** in CD_3OD.

Table S1. Gibbs free energy and Boltzmann population of low energy of 3*S,* 4*R*, 7S, 8*R*, 9*R*, 16*S*, 18*R*, 21*R* **-1** in MeOH.

Conformers of 3*S,* 4*R*, 7S, 8*R*, 9*R*, 16*S*, 18*R*, 21*R* **-1**	ΔG (kcal/mol)	P
**1**a	0.81	0.201
**1**b	0.00	0.793
**1**c	2.86	0.006

Table S2. Gibbs free energy and Boltzmann population of low energy of 3*S,* 4*R*, 8*R*, 9*R*, 16*S*, 18*R*, 21*R* **-3** in MeOH.

Conformers of 3*S,* 4*R*, 8*R*, 9*R*, 16*S*, 18*R*, 21*R* **-3**	ΔG (kcal/mol)	P
**3**a	0.75	0.146

**3**b	1.10	0.080
**3**c	0.45	0.243
**3**d	0.00	0.517
**3**e	2.15	0.014

Table S3. Gibbs free energy and Boltzmann population of low energy of 3*S*, 4*R*, 8*R*, 9*R*, 16*S*, 18*R*, 21*R* -**4** in MeOH.

Conformers of 3*S*, 4*R*, 7*S*, 8*R*, 9*R*, 16*S*, 21*R* -**4**	ΔG (kcal/mol)	P
**4**a	0.99	0.158
**4**b	0.00	0.840
**4**c	5.00	0.000
**4**d	3.76	0.002

Table S4. Gibbs free energy and Boltzmann population of low energy of 9*S*, 10*S*, 12*S*-**6** in MeOH.

Conformers of 9*S*, 10*S*, 12*S*-**6**	ΔG (kcal/mol)	P
**6**a	0.32	0.361
**6**b	0.00	0.623
**6**c	2.17	0.016

Table S5. Gibbs free energy and Boltzmann population of low energy of 2*S*, 4*S*, 8*S* -**7** in MeOH.

Conformers of 2*S*, 4*S*, 8*S*-**7**	ΔG (kcal/mol)	P
**7**a	0.26	0.20
**7**b	1.03	0.04
**7**c	0.00	0.21

**7d**	1.02	0.04
**7e**	0.80	0.05
**7f**	0.11	0.17
**7g**	0.59	0.08
**7h**	1.65	0.01
**7i**	0.14	0.16
**7j**	0.86	0.05

Table S6. Gibbs free energy and Boltzmann population of low energy of 2*R*, 4*R*, 8*S* -**7** in MeOH.

Conformers of 2*R*, 4*R*, 8*S*-**7**	ΔG (kcal/mol)	P
**7a**	1.27	0.04
**7b**	1.68	0.02
**7c**	0.96	0.06
**7d**	1.32	0.03
**7e**	1.08	0.05
**7f**	0.56	0.12
**7g**	0.07	0.27
**7h**	0.00	0.31
**7i**	1.36	0.05
**7j**	1.36	0.05

Table S7. Gibbs free energy and Boltzmann population of low energy of 2*S*, 4*R*, 8*S* -**7** in MeOH.

Conformers of 2*S*, 4*R*, 8*S*-**7**	ΔG (kcal/mol)	P
**7a**	1.36	0.03
**7b**	0.86	0.06
**7c**	1.72	0.02

7d	1.24	0.03
7e	1.03	0.05
7f	1.40	0.03
7g	0.47	0.12
7h	0.05	0.25
7i	0.00	0.27
7j	1.36	0.03
7k	1.04	0.05
7l	1.32	0.03
7m	1.36	0.03
7n	1.39	0.03

Table S8. Gibbs free energy and Boltzmann population of low energy of 2*R*, 4*S*, 8*S* -**7** in MeOH.

Conformers of 2*R*, 4*S*, 8*S*-**7**	ΔG (kcal/mol)	P
7a	0.02	0.16
7b	1.29	0.02
7c	0.00	0.17
7d	1.34	0.02
7e	1.34	0.02
7f	1.28	0.02
7g	0.02	0.16
7h	0.95	0.03
7i	0.57	0.06
7j	0.05	0.15
7k	0.48	0.07
7l	0.46	0.08
7m	0.96	0.03
7n	1.91	0.01
